# Direct C–H Allylation of Unactivated Alkanes by Cooperative W/Cu Photocatalysis

**DOI:** 10.1021/acs.orglett.2c02887

**Published:** 2022-09-13

**Authors:** Pol Martínez-Balart, Balázs L. Tóth, Álvaro Velasco-Rubio, Martín Fañanás-Mastral

**Affiliations:** Centro Singular de Investigación en Química Biolóxica e Materiais Moleculares (CiQUS), Universidade de Santiago de Compostela, 15782 Santiago de Compostela, Spain

## Abstract

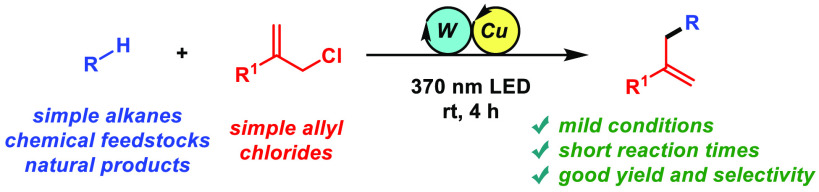

Here we report a photocatalytic methodology that enables the direct allylation of strong aliphatic C–H bonds with simple allylic chlorides. The method relies on a cooperative interaction of two metal catalysts in which the decatungstate anion acts as a hydrogen-atom abstractor generating a nucleophilic carbon-centered radical that engages in an S_H_2′ reaction with an activated allylic π-olefin–copper complex. Because of this dual catalysis, the protocol allows for the functionalization of a range of chemical feedstocks and natural products under mild conditions in short reaction times.

Allylation reactions are some of the most powerful and useful tools for the construction of C–C bonds.^1^ Installation on an allyl group provides a versatile synthetic handle for further modification. Moreover, its ubiquitous presence in bioactive compounds (or advanced intermediates) makes these transformations very valuable for the medicinal and pharmaceutical industries.^2^ Traditionally, allylation reactions have been carried out with prefunctionalized substrates,^3^ which often require prior preparation and involve the formation of byproducts. In this context, the direct allylation of C–H bonds offers a more atom-efficient and straightforward way to incorporate the allyl group into readily available substrates.^4^ Methodologies for C(sp^3^)–H allylation are mainly based on the use of activated systems such as C(sp^3^)–H bonds adjacent to carbonyl groups^1a,5^ and heteroatoms,^6^ or benzylic and allylic positions (Scheme 1a).^7^ However, the direct allylation of unactivated C–H bonds has been less explored.

**Scheme 1:**
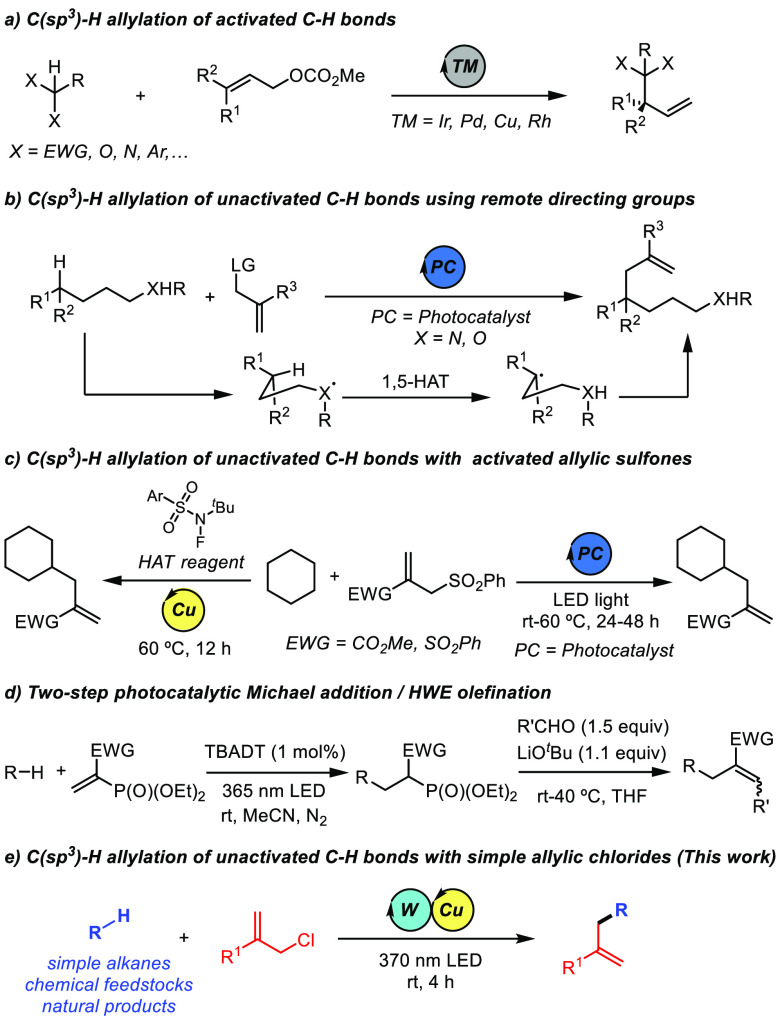
Methods for C(sp^3^)–H Allylation

Recently, radical-mediated hydrogen-atom transfer (HAT) has become a useful platform for the functionalization of aliphatic C–H bonds.^8^ In this context, remote allylation of unactivated C–H bonds has been achieved by protocols involving photoredox-promoted generation of oxygen-^9^ and nitrogen-centered^10^ radicals followed by 1,5-HAT and subsequent C–C bond formation (Scheme 1b). In contrast, the C–H allylation of unactivated alkanes lacking this type of directing group remains challenging. The groups of Kamijo,^11^ Wu,^12^ and Liang and Niu^13^ disclosed catalytic approaches for the direct allylation of C(sp^3^)–H bonds by using either organic HAT photocatalysts^11,12^ or stoichiometric HAT reagents.^13^ Despite these advances, these methods are still plagued by the requirement of long reaction times and/or high temperatures, and the use of allyl sulfones activated by Michael acceptors (Scheme 1c). Recently, Noël and co-workers reported a two-step strategy for aliphatic C–H bond allylation that involves the trapping of a C-centered radical generated by HAT photocatalysis with a vinyl phosphonate, followed by a Horner–Wadsworth–Emmons olefination of the resulting radical addition product (Scheme 1d).^14^

Given the outstanding photochemical activity of the decatungstate anion ([W_10_O_32_]^4–^, DT) and its ability to function as an efficient HAT photocatalyst in several C–H functionalization reactions,^15,16^ we questioned whether we could merge the DT photocatalysis with a copper-catalyzed allylic alkylation to develop a C–H allylation of unactivated alkanes with simple allylic chlorides. In this mechanistic scenario, the photoexcited DT anion would be responsible for the selective generation of an alkyl radical while the copper catalyst would activate the allylic substrate (Scheme 1e). This cooperative interaction would facilitate and substantially accelerate the reaction, thus minimizing substrate and/or product degradation that could arise from long irradiation times.

We began our investigation by exploring the reaction between cyclohexane **1** and allylic chloride **2** in acetonitrile using tetrabutylammonium decatungstate (TBADT) as the HAT photocatalyst under near-ultraviolet light irradiation (Kessil 43 W 370 nm LED). After screening several reaction parameters (see the Supporting Information), we found that product **3** could be isolated in 62% yield after just 4 h when using 1 mol % TBADT in combination with a co-catalyst comprising 5 mol % CuCl and 6 mol % PPh_3_ with collidine as the base (Table 1, entry 1).

**Table 1:**
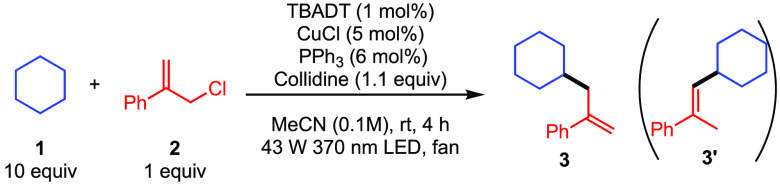
Optimization Studies

entry^a^	variation from standard conditions	conversion (%)^b^	**3** (%)^c^
1	none	>95	62
2	without the Cu complex	70	29
3	without PPh_3_	80	50
4	PCy_3_ or P(OPh)_3_	80	50
5	CuI or Cu(MeCN)_4_PF_6_	91	56
6	[Ni], [Pd], [Co], or [Zn] instead of CuCl	>95	<38
7	K_3_PO_4_	>95	49
8	DBU, 24 h	80	10
9	pyridine, 24 h	66	30
10	no base, 24 h	82	51^d^
11	5 equiv of **1**	90	51
12	without TBADT or light	–	–

a Reactions run on a 0.5 mmol scale.

b Conversion was determined by ^1^H NMR analysis using 1,3,5-trimethoxybenzene as the internal standard.

c Yield of the isolated product.

d Obtained as a 10:1 mixture of products **3′** and **3**.

The use of the copper/phosphine catalyst turned out to be crucial for the success of the reaction. Notably, when the reaction was performed in the absence of the copper complex, product **3** was obtained in low yield (entry 2). Importantly, we generally observed that prolonged irradiation times caused some product degradation. The absence of triphenylphosphine (entry 3) and the use of other types of ligands (entry 4) were also detrimental to both conversion and yield. CuCl could be replaced by other copper complexes without a significant loss of efficiency, although product **3** was obtained in slightly diminished yield in those cases (entry 5). Other co-catalysts based on different transition metals such as Ni, Pd, Co, or Zn systematically led to lower yields (entry 6). The nature of the base also played an important role in the reaction. Potassium phosphate also proved to be efficient for this transformation, although to a lesser extent than collidine (entry 7). In contrast, other nitrogen bases such as 1,8-diazabicyclo(5.4.0)undec-7-ene (DBU) and pyridine led to poor results (entries 8 and 9, respectively). A significant decrease in efficiency was observed in the absence of a base (entry 10). Interestingly, in this case, formal C–H alkenylation product **3′** was obtained likely due to HCl-promoted isomerization.^17^ The reaction can be performed with fewer equivalents of cyclohexane (5 equiv) albeit with a slightly lower conversion and yield (entry 11). Finally, control experiments showed that the reaction does not proceed in the absence of TBADT or light (entry 12). Evaluation of other leaving groups revealed that allylic bromides, sulfonates, or carbonates led to the C–H allylation product in almost negligible yield mainly due to decomposition of the allyl substrate under reaction conditions, while acetates or phosphates remained almost unreacted (see the Supporting Information for details). 1,2-Disubstituted allyl compounds (e.g., cinnamyl chloride) did not provide the C–H allylation product either.

Having established optimized conditions, we set out to explore the scope of this C–H allylation reaction (Scheme 2). The W/Cu catalytic system proved to be remarkably effective with a range of allylic chlorides. Aryl-substituted substrates were coupled with cyclohexane or cyclooctane providing the corresponding products **4–13** in good yields regardless of the electronic properties and position of the substituents on the aromatic ring. Importantly, allylic chlorides bearing aliphatic substituents such as a trimethylsilylmethyl group (**14**) or an even more challenging 3-butenyl substituent (**15**) could also be selectively coupled with cyclohexane without observing functionalization of other C–H bonds of the structure. Moreover, allylic chlorides bearing an extra chlorine atom or a chloromethyl group, which offer an extra handle for further synthetic modification, were also suitable reaction partners yielding the corresponding products **16** and **17** in moderate to good yields. In the case of the latter, the monoalkylation product could be selectively obtained largely due to the short reaction time because prolonged reaction times resulted in the formation of mixtures of mono- and dialkylation products.

**Scheme 2:**
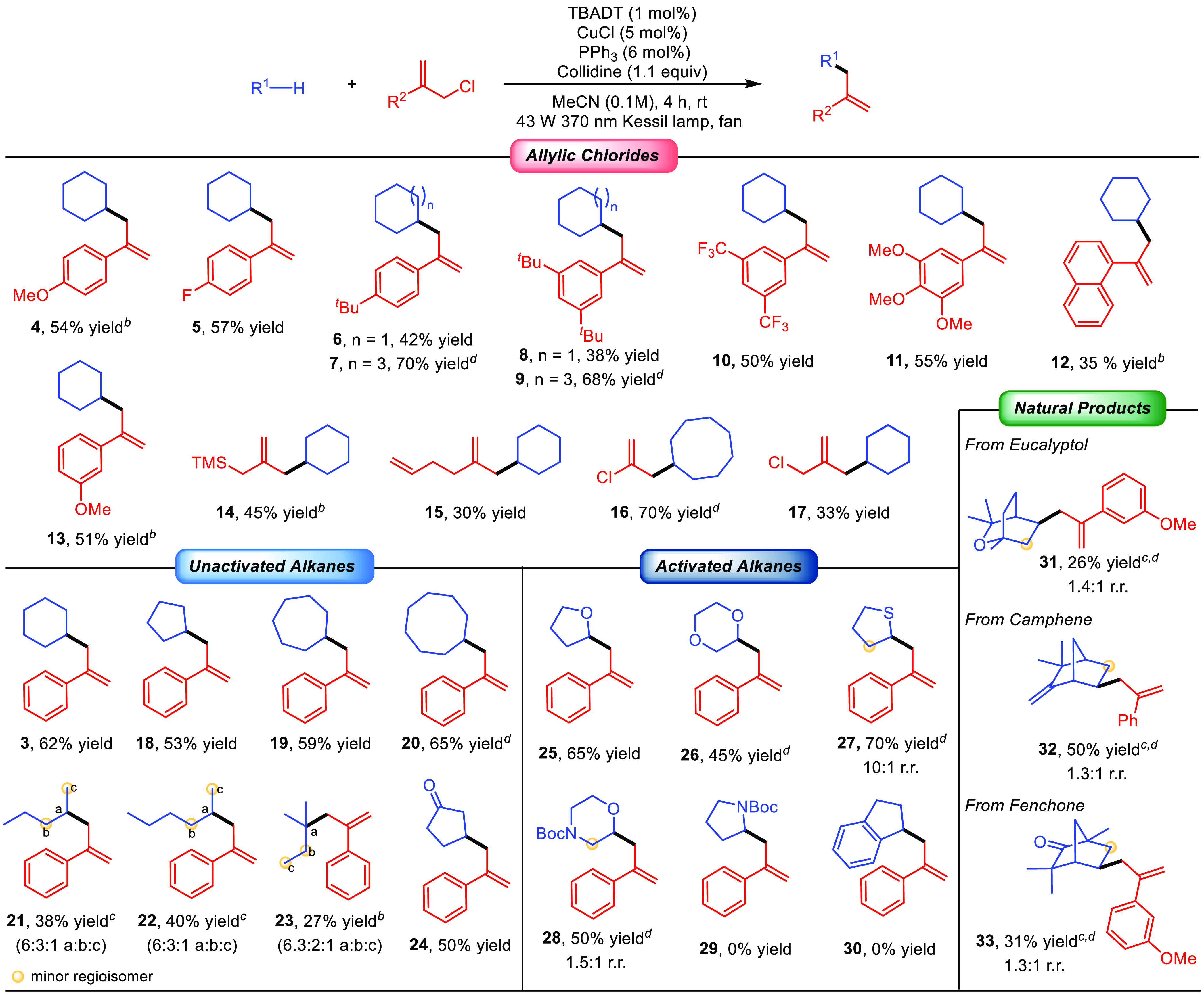
Substrate Scope Reactions performed on a 0.5 mmol scale using 10 equiv of the alkane, unless otherwise noted. Yields refer to the isolated product. Reaction run over 8 h. Reaction run over 16 h. Reaction carried out with 5 equiv of the alkane.

We then turned to evaluate the scope of the alkanes that are suitable for this C–H allylation reaction. Cycloalkanes ranging in length from five to eight carbons were allylated in good yields (**18–20**). Linear aliphatic systems were slightly less efficient, although they provided the corresponding products **21–23** with good regioselectivity (60–70%). Site-selective allylation of methylene C–H bonds over the methyl C–H bonds was observed in *n*-pentane and *n*-hexane (**21** and **22**, respectively), with a preference over the less sterically demanding methylene groups in the latter. Furthermore, methine C–H functionalization was preferred over methylene C–H functionalization as illustrated by the C–H allylation of isopentane (**23**). Full regiocontrol was observed for the C–H allylation of the β-position of cyclopentanone (**24**), according to the preference of the electrophilic excited state of decatungstate for the most hydridic C–H bonds.^18^

This C–H allylation reaction is not restricted to only unactivated systems. Cyclic ethers and thioethers such as tetrahydrofuran, 1,4-dioxane, and tetrahydrothiophene could be selectively allylated at the α-oxy position in good yield with excellent regioselectivity (**25–27**). Surprisingly, *N*-Boc morpholine underwent C–H allylation predominantly at the α-oxy C–H bond (**28**, 60% selectivity). This is in contrast with other photoinduced C–H functionalization reactions of this heterocycle in which selective functionalization occurs at the α-amino C–H bond.^16a,19^ Along this line, we observed that *N*-Boc pyrrolidine did not undergo this transformation as well as other stabilized H-donors such as Indane. These results suggest that a high level of stabilization of the photogenerated alkyl radical results in nonreactive species for allylic substitution under optimized conditions.

Finally, to illustrate the synthetic potential of this methodology, we performed the late-stage C–H allylation of aliphatic natural products such as Eucalyptol (**31**, 26% yield, 1.4:1 rr), Camphene (**32**, 50% yield, 1.3:1 rr), and Fenchone (**33**, 31% yield, 1.3:1 rr). These examples show the capability of this transformation to install a synthetically versatile allyl group onto complex aliphatic substrates without the need for directing groups.

To gain insight into this transformation, some mechanistic investigations were performed. When deuterated allylic chloride **2**-***d*** was used under optimal conditions, product **3**-***d*** was exclusively obtained (Scheme 3a). This deuterium labeling experiment supports an S_H_2′ mechanism, while it suggests that no isomerization of the allylic chloride occurs through homolytic C–Cl cleavage.^10b^ The use of a radical scavenger such as TEMPO totally suppressed the formation of product **3**. In that case, only the cyclohexyl-TEMPO adduct was detected while no formation of any allyl-TEMPO adduct was observed (Scheme 3b). Furthermore, a radical-clock experiment was performed to assess the possible formation of the free chlorine radical that might also serve as the HAT radical initiator.^20^ When the reaction was run in the presence of diallyl sulfonamide **35**, which has been described as a chlorine radical trap,^20d^ no chlorinated product arising from a chlorine radical addition was observed, somewhat ruling out this possibility (Scheme 3c).

**Scheme 3:**
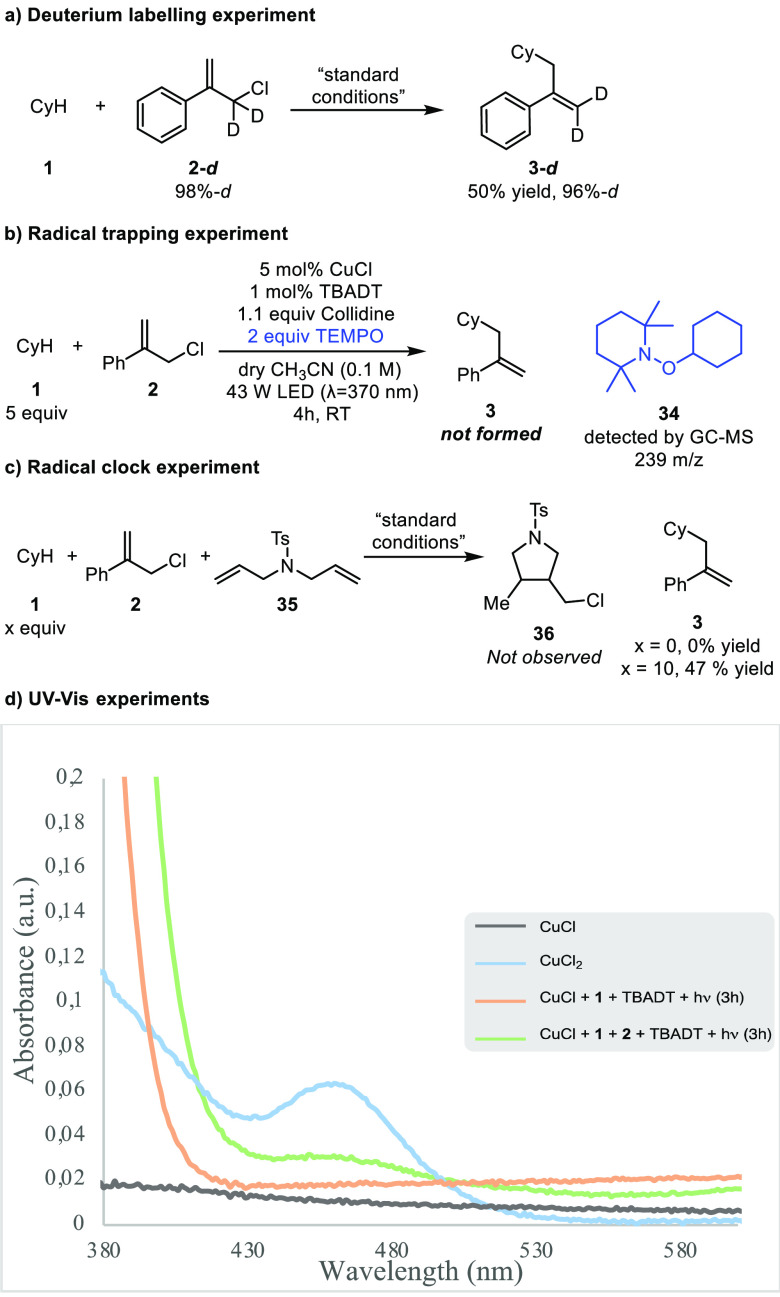
Mechanistic Investigations

We next sought to explore the role of the Cu(I) complex. UV/vis absorption spectroscopy studies (Scheme 3d) demonstrated that Cu(II) species were not formed when a mixture of TBADT (3 mol %), CuCl (3 mol %), and cyclohexane (10 equiv) in MeCN was irradiated for 3 h. In contrast, formation of Cu(II) species was observed (band at λ ≈ 465 nm)^20b,21^ when the same experiment was performed in the presence of allylic chloride **2**. These results suggest that the alkyl radical generated by TBADT-promoted HAT does not interact with the Cu(I) complex to lead to an alkyl–Cu(II) intermediate. Conversely, direct attack of the alkyl radical on an allylic π-olefin–Cu(I) complex^22^ would afford the product and an oxidized LCuCl_2_ complex. Evidence for the formation of the allyl–Cu(I) complex could be obtained by NMR studies (see section 10.5 of the Supporting Information). Along this line, the better performance observed when PPh_3_ was used as the ligand (Table 1, entries 1 and 10) may be due to a maximized molecular orbital overlap between the phosphine–copper(I) complex and the allylic chloride (i.e., interaction of the 3d_*xz*_ Cu orbital with the allylic C=C π* and C–Cl σ* orbitals).^23^

On the basis of these mechanistic experiments and previous photochemical studies of TBADT-mediated processes,^15,16^ we propose the mechanism depicted in Scheme 4. First, photoexcitation of DT anion [W_10_O_32_]^4–^ (**A**) would produce triplet excited state [W_10_O_32_]^4–^* (**B**), which abstracts a hydrogen atom from the alkane to generate a nucleophilic carbon-centered radical **D** upon formation of reduced species **C** (H[W_10_O_32_]^5–^). Addition of radical **D** to allylic π-olefin–Cu(I) complex **F** through an S_H_2′ pathway^24^ would result in the formation of product **3** and (Ph_3_P)CuCl_2_ complex **G**. A final collidine-assisted single-electron transfer between Cu species **G** and reduced decatungstate H[W_10_O_32_]^5–^ (*E*_1/2_ CuCl_2_/CuCl = 0.56 V vs SCE;^25^*E*_1/2_ [W_10_O_32_]^4–^/[W_10_O_32_]^5–^ = −0.97 V vs SCE^26^) would regenerate the Cu(I) catalyst as well as the TBADT, closing both catalytic cycles.

**Scheme 4:**
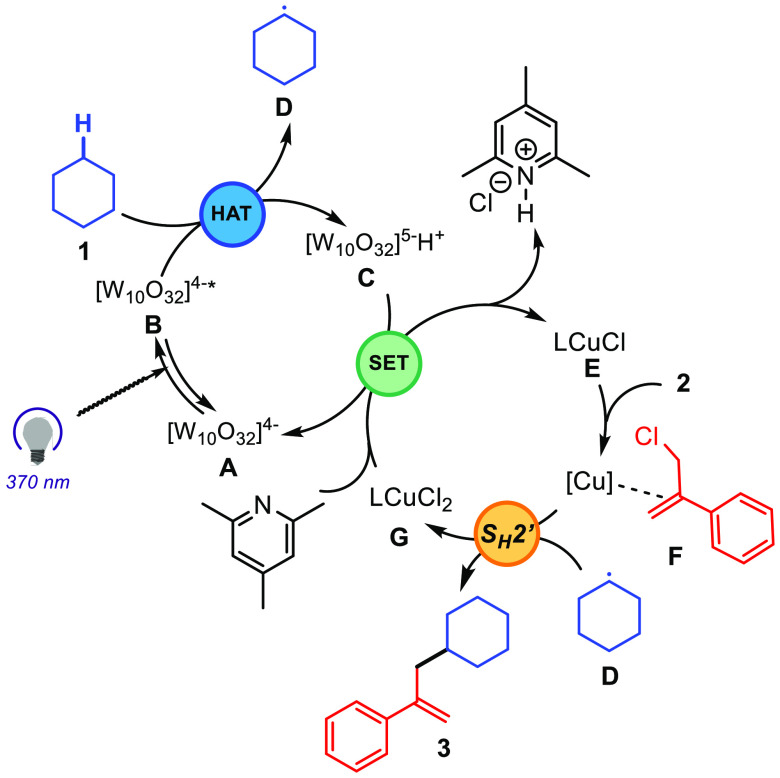
Proposed Mechanism for the W/Cu-Catalyzed C(sp^3^)–H Allylation of Alkanes

In summary, we have developed a practical methodology that allows the direct C–H allylation of strong aliphatic bonds with simple allylic chlorides. The combination of a light-driven decatungstate-mediated hydrogen-atom transfer and copper catalysis is key to enabling an efficient and fast coupling that prevents product degradation. Under this dual catalysis regime, a range of synthetically versatile C–H allylated structures can be obtained in good yields directly from chemical feedstocks and even complex natural products.

## Supporting Information Available

List of starting materials, optimization studies, experimental procedures, and compound characterization data (PDF)
