# Supplementary material for: Direct C–H Allylation of Unactivated Alkanes by Cooperative W/Cu Photocatalysis
Source: Org Lett. 2022 Sep 13;24(37):6874–9. doi: 10.1021/acs.orglett.2c02887 (PMC9513794; doi:10.1021/acs.orglett.2c02887)

Direct C−H Allylation of Unactivated Alkanes by Cooperative W/Cu Photocatalysis

Pol Martínez-Balart, Balázs L. Tóth, Álvaro Velasco-Rubio and Martín Fañanás-Mastral*

Centro Singular de Investigación en Química Biolóxica e Materiais Moleculares (CiQUS), Universidade de Santiago de Compostela, 15782, Santiago de Compostela, Spain

Supporting Information

Table of Contents

1. **General Experimental Details……………………………………………………..….….S2**
2. **List of Starting Materials……………………………………………….….……..S3**
3. **Synthesis of Starting Materials ..…………………………………….…......……...S4**
4. **General Procedure for the W/Cu catalyzed C-H allylation of alkanes………………...S7**
5. **Reaction Setup.……………………………..……………………...……..…S7**
6. **Compound characterization……………...……………………………………………S8**
7. **Procedure for the synthesis of 16 at 1.5 mmol scale …….…………………...……...S16**
8. **Optimization Studies …………………………………………………………....S17**
9. **Control Experiments ……………………………………………………........S20**
10. **Mechanistic Studies……………………………………………………...……S21**
11. **NMR Spectra……………………………………………………………………..S32**

1. General Experimental Details

All reactions were performed under argon atmosphere using oven dried glassware and using standard Schlenk techniques. Solvents were dried using an MBraun SPS 800 system.

All chemicals were purchased from Acros Organics Ltd., Aldrich Chemical Co. Ltd., Alfa Aesar, Apollo, Strem Chemicals Inc., Fluorochem Ltd. or TCI Europe N.V. chemical companies and used without further purification, unless otherwise noted.

Analytical thin layer chromatography was carried out on silica-coated aluminum plates (silica gel 60 F254 Merck) and compounds were visualized using 254 nm UV light or by oxidation treatment (solution of 1.5 g of $KMnO_4$, 10 g of $KHCO_3$ and 1.25 mL of an aqueous solution of NaOH (10 (w/w)%) in 200 mL of H_2O) and heat.

Flash column chromatography was performed on silica gel 60 (Merck, 230-400 mesh) without previous deactivation, unless otherwise stated.

UV measurements were made in a *Jasco V-630* spectrophotometer coupled to a *Jasco ETC-717* temperature controller, using a standard *Hellma* semi-micro cuvette (108.002-QS) with a light path of 10 mm. Measurements were made at 20 °C. Acquisition parameters were: 300-900 nm range, scan speed of 200 nm/min, resolution of 0.2 nm.

Light-promoted reactions: The LED used are Kessil PR 160 370 nm (43 W) and Kessil PR 160 390 nm (52 W). The reaction vessel is an 8 mL vial of borosilicate glass.

GC-MS analyses were performed in an Agilent instrument GC-8890 equipped with Chemical Ionization (CI) MS-5977B detector.

High Resolution Mass spectrometry was carried out on a Bruker microTOF spectrometer using APCI or ESI.

^{1}H-,^{13}C and ^{19}F-NMR experiments were carried out using a Bruker AVIII-500 MHz or a Varian Mercury 300 MHz or Agilent VNMRS-300 MHz NMR spectrometers. Chemical shift values are reported in ppm with the solvent resonance as the internal standard ($CHCl_3$: δ 7.26 for ^{1}H, δ 77.16 for ^{13}C). Coupling constants (J) are given in Hertz (Hz). Multiplicities are reported as follows: *bs* = broad singlet, *s* = singlet, *d* = doublet, *t* = triplet, *q* = quartet, *p* = pentet, *m* = multiplet or as a combination of them.

Tetrabutylammonium decatungstate (TBADT) was synthesized according to a literature procedure[1].

[1]Perry, I. B.; Brewer, T. F.; Sarver, P. J.; Schultz, D. M. DiRocco, D. A.; MacMillan, D. W. C. *Nature*, **2018**, *560*, 70-75.

2. List of Starting Materials

Unactivated alkanes

Alkanes **1**, **40-42** were purchased from Aldrich Chemical Co. Ltd., **38** and **43** were purchased from TCI Europe N.V., **37** was purchased from Apollo and **39** was purchased from Alfa Aesar.

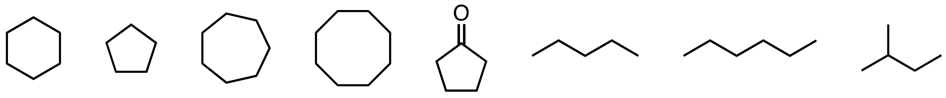

1 **37** **38** **39** **40** **41** **42** **43**

Activated alkanes

Compounds **44** and **47** were purchased from Across Organics Ltd., **45** was purchased from Fluorochem Ltd., **48** was purchased from Aldrich Chemical Co. Ltd. and **46** was synthesized according to literature procedure.[2]

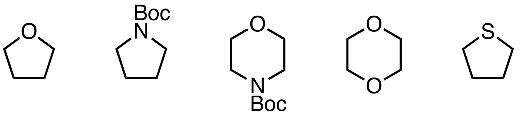

44 **45** **46** **47** **48**

Allyl chlorides

Allylic chlorides **58** and **60** were purchased from Aldrich Chemical Co. Ltd., **57** was purchased from TCI Europe N.V., and allylic chlorides **2**,[3] **50**,[3] and **59**[3] were prepared as reported in the literature.

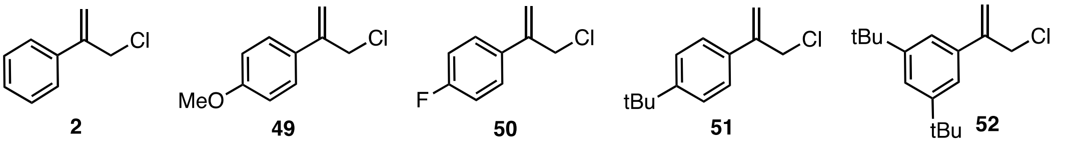

2 **49** **50** **51** **52**

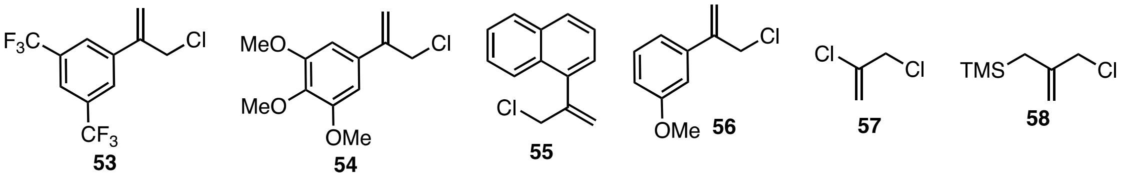

53 **54** **55** **56** **57** **58**

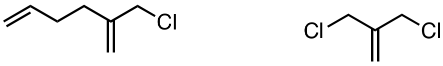

59 **60**

Natural products

Natural products **61** and **63** were purchased from TCI Europe N.V., and **62** was purchased from Aldrich Chemical Co. Ltd.

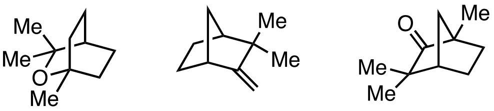

61 *Eucalyptol* **62** *Camphene* **63** *Fenchone*

---

[2] Mojtahedi, M. M.; Niknejad, N.; Veisi, H. *Lett. Org. Chem.* **2013**, *10*, 121-125.
[3] B. Xu, U. Tambar, *ACS Catal.* **2019**, *9*, 4627–4631.

3. Synthesis of allylic chlorides 49, 51-56

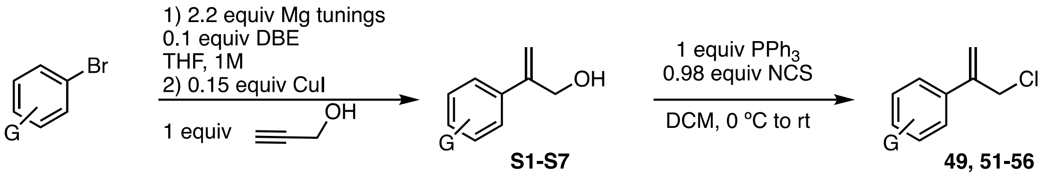

The corresponding Grignard reagent (2 equiv, 20 mmol) was made according to a literature procedure.[4] The resulting solution was cooled at 0 ºC and CuI (15 mol%) was added. The mixture was stirred for 30 min at that temperature and a solution of the propargyl alcohol (1 equiv, 10 mmol) in THF (3 M) was added dropwise. Then the reaction was heated to 50 ºC for 16 h. After that time, the reaction was quenched with an aqueous saturated solution of NH_4Cl and extracted with AcOEt (2 x 50 mL). The combined organic layers were dried over $MgSO_4$ and concentrated. The crude product was purified by flash column chromatography using as eluent a mixture of Hexanes/AcOEt to provide the desired alcohol (**S1-S7**).

To a solution of the corresponding alcohol (1 equiv, 3 mmol) in DCM (0.5 M) cooled at 0 ºC, PPh_3 (1 equiv, 3 mmol) was added, and the mixture was stirred for 5 min. Then, NCS (0.98 equiv, 2.94 mmol) was added dropwise for 10 min. The mixture was stirred at 0 ºC for 30 min, and then 30 min at rt. The reaction was quenched with water (5 mL) and extracted with DCM (3 x 15 mL). The combined organic layers were dried over $MgSO_4$ and concentrated. The crude product was purified by flash column chromatography using as eluent a mixture of Hexanes/AcOEt to provide the desired allylic chloride **49, 51-56**.

Alcohols **S1**, **S2**, **S5-S7** have been previously characterized and the spectroscopic signals were in accordance to reported literature.[5-8]

2-(3,5-Di-*tert*-butylphenyl)prop-2-en-1-ol (S3)

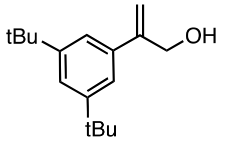

Obtained as a pale-yellow solid in 69% yield (1.7 g) after flash column chromatography (Hexanes/AcOEt 4:1).

^{1}H NMR (300 MHz, $CDCl_3$) δ 7.42 (t, *J* = 1.7 Hz, 1H), 7.30 (d, *J* = 1.8 Hz, 2H), 5.45 (s, 1H), 5.37 (s, 1H), 4.58 (bs, 2H), 1.75 (bs, 1H), 1.37 (d, *J* = 1.2 Hz, 18H). **^{13}C NMR** (75 MHz, $CDCl_3$) δ 151.0 (C), 148.7 (C), 138.1 (C), 122.3 (CH), 120.6 (CH), 112.2 (CH_2), 65.4 (CH_2), 35.0 (C), 31.6 (6xCH_3).

2-(3,4,5-Trimethoxyphenyl)prop-2-en-1-ol (S4)

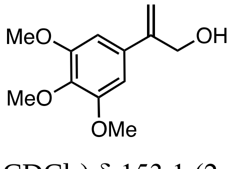

Obtained as white solid in 27% yield (610 mg) after flash column chromatography (Hexanes/AcOEt 3:2 to 1:1).

^{1}H NMR (300 MHz, $CDCl_3$) δ 6.63 (s, 2H), 5.36 (s, 1H), 5.29 (s, 1H), 4.45 (bs, 2H), 3.83 (s, 6H), 3.81 (s, 3H), 2.32 (s, 1H). **^{13}C NMR** (75 MHz, $CDCl_3$) δ 153.1 (2xC), 147.4 (C), 138.0 (C), 134.6 (C), 112.5 (CH_2), 103.6 (2xCH), 65.0 (CH_2), 60.9 (CH_3), 56.1 (2xCH_3).

[4] Velasco-Rubio, A.; Alexy, E. J.; Yoritate, M.; Wright, A. C.; Stoltz, B. M. *Org. Lett.* **2019**, *21*, 8962−8965.

1-(3-Chloroprop-1-en-2-yl)-4-methoxybenzene (49)[5]

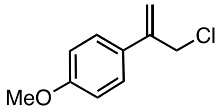

Obtained from 2-(4-methoxyphenyl)prop-2-en-1-ol (**S1**)[6] as pale-yellow oil in 75% yield (410 mg) after flash column chromatography (Hexanes/AcOEt 95:5). **^{1}H NMR** (300 MHz, $CDCl_3$) δ 7.42 (m, 2H), 6.89 (m, 2H), 5.48 (s, 1H), 5.35 (s, 1H), 4.44 (s, 2H), 3.81 (s, 3H).

1-(*tert*-Butyl)-4-(3-chloroprop-1-en-2-yl)benzene (51)

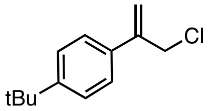

Obtained from 2-(4-(*tert*-butyl)phenyl)prop-2-en-1-ol (**S2**)[6] as a yellow oil in 80 % yield (500 mg) after flash column chromatography (Hexanes/AcOEt 20:1).
^{1}H NMR (300 MHz, $CDCl_3$) δ 7.48 – 7.36 (m, 4H), 5.56 (d, J = 0.9 Hz, 1H), 5.42 (d, J = 0.9 Hz, 1H), 4.47 (bs, 2H), 1.33 (s, 9H). **^{13}C NMR** (75 MHz, $CDCl_3$) δ 151.5 (C), 143.8 (C), 134.8 (C), 125.9 (CH), 125.6 (CH), 116.2 (CH_2), 46.7 (CH_2), 34.7 (C), 31.4 (3xCH_3). **HRMS** (APCI, m/z): calculated for $C_{13}H_{18}Cl$ [M^+ + H]: 209.1092; found: 209.1090.

1,3-Di-*tert*-butyl-5-(3-chloroprop-1-en-2-yl)benzene (52)

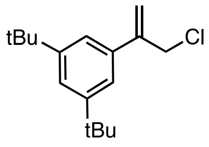

Obtained as a pale-yellow oil in 80% yield (640 mg) after flash column chromatography (Hexanes/AcOEt 20:1).
^{1}H NMR (300 MHz, $CDCl_3$) δ 7.41 (s, 1H), 7.32 (d, J = 1.8 Hz, 2H), 5.55 (s, 1H), 5.46 (s, 1H), 4.49 (bs, 2H), 1.35 (s, 18H). **^{13}C NMR** (75 MHz, $CDCl_3$) δ 150.9 (C), 145.0 (C), 137.3 (C), 122.6 (CH), 120.6 (CH), 116.4 (CH_2), 47.0 (CH_2), 35.1 (C), 31.6 (6xCH_3). **HRMS** (APCI, m/z): calculated for $C_{17}H_{26}Cl$ [M^+ + H]: 265.1718; found: 265.1722.

1-(3-Chloroprop-1-en-2-yl)-3,5-bis(trifluoromethyl)benzene (53)

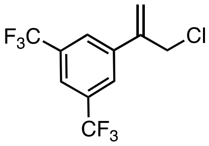

Obtained from 2-(3,5-bis(trifluoromethyl)phenyl)prop-2-en-1-ol (**S4**)[7] as yellow oil in 62% yield (530 mg) after flash column chromatography (Hexanes/AcOEt 9:1).
^{1}H NMR (300 MHz, $CDCl_3$) δ 7.91 (s, 2H), 7.85 (s, 1H), 5.69 (s, 1H), 5.66 (s, 1H), 4.49 (bs, 2H). **^{13}C NMR** (75 MHz, $CDCl_3$) δ 142.1 (C), 140.0 (C), 132.2 (q, J = 33.4 Hz), 126.6 (CH), 123.4 (q, J = 271.7 Hz) (C), 122.1 (CH), 120.2 (CH_2), 45.8 (CH_2). **^{19}F NMR** (282 MHz, $CDCl_3$) δ -63.0. **HRMS** (APCI, m/z): calculated for $C_{10}H_8ClF_3$ [M^+ - CF_3 + H]: 219.0188; found: 219.0185.

5-(3-Chloroprop-1-en-2-yl)-1,2,3-trimethoxybenzene (54)

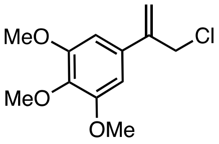

Obtained as Colorless oil in 47% yield (340 mg) after flash column chromatography (Hexanes/AcOEt 4:1).
^{1}H NMR (300 MHz, $CDCl_3$) δ 6.69 (s, 2H), 5.50 (s, 1H), 5.43 (s, 1H), 4.43 (s, 2H), 3.88 (s, 6H), 3.86 (s, 3H). **^{13}C NMR** (75 MHz, $CDCl_3$) δ 153.3 (C), 144.1 (C), 138.5 (C), 133.7 (C), 116.7 (CH), 103.9 (CH_2), 61.0 (CH_3), 56.3 (2xCH_3), 46.8 (CH_2). **HRMS** (APCI, m/z): calculated for $C_{12}H_{16}ClO_3$ [M^+ + H]: 243.0782; found: 243.0780.

---

[5] Matyus, P; Chai, C. L. L. *et al. J. Med. Chem.* **2015**, *58*, 1400-1419.
[6] Hollingworth, C.; Hazari, A.; Hopkinson, M. N.; Tredwell, M.M Benedetto, E.; Huiban, M.; Gee, A. D.; Brown, J. M.M Gouverneur, V. *Angew. Chem. Int. Ed.* **2011**, *50*, 2613-2617.
[7] Sun, X.; Frimpong, K.; Tan, K. L. *J. Am. Chem. Soc.* **2010**, *132*, 11841-11843.

1-(3-Chloroprop-1-en-2-yl)naphthalene (55)

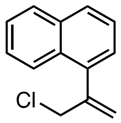

Obtained from 2-(naphthalen-1-yl)prop-2-en-1-ol (**S6**)[8] as Colorless oil in 50% yield (300 mg) after flash column chromatography (Hexanes/AcOEt 20:1). **^{1}H NMR** (300 MHz, $CDCl_3$) δ 8.01 – 7.93 (m, 1H), 7.92 – 7.80 (m, 2H), 7.55 – 7.44 (m, 3H), 7.38 (d, J = 7.0 Hz, 1H), 5.85 (s, 1H), 5.37 (s, 1H), 4.45 (s, 2H). **^{13}C NMR** (75 MHz, CDCl3) δ 144.0 (C), 137.6 (C), 133.9 (C), 131.5 (C), 128.9 (CH), 128.6 (CH), 128.3 (CH), 126.4 (CH), 126.1 (CH), 126.0 (CH), 125.3 (CH), 119.8 (CH_2), 48.7 (CH_2). **HRMS** (APCI, m/z): calculated for $C_{13}H_{11}Cl$ [M^+ + H]: 203.0549; found: 203.0545.

1-(3-Chloroprop-1-en-2-yl)-3-methoxybenzene (56)[5]

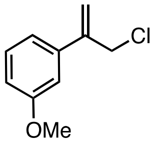

Obtained from 2-(3-methoxyphenyl)prop-2-en-1-ol (**S7**)[9] as colorless oil in 50% yield (270 g) after flash column chromatography (Hexanes/AcOEt 95:5). **^{1}H NMR** (300 MHz, $CDCl_3$) δ 7.33 – 6.83 (m, 4H), 5.49 (s, 1H), 5.26 (s, 1H), 4.50 (m, 2H), 3.83 (s, 3H).

---

[8] Piel, I.; Steinmetz, M.; Hirano, K.; Fröhlich, R.; Grimme, S.; Glorius, F.; *Angew. Chem. Int. Ed.* **2011**, *50*, 4983-4987.
[9] Zhang, Y.; Xing, H.; Xie, W.; Wan, X.; Lai, Y.; Ma, D. *Adv. Synth. Catal.* **2013**, *355*, 68-72.

4. General procedure for the W/Cu catalyzed C-H allylation of alkanes with allylic chlorides

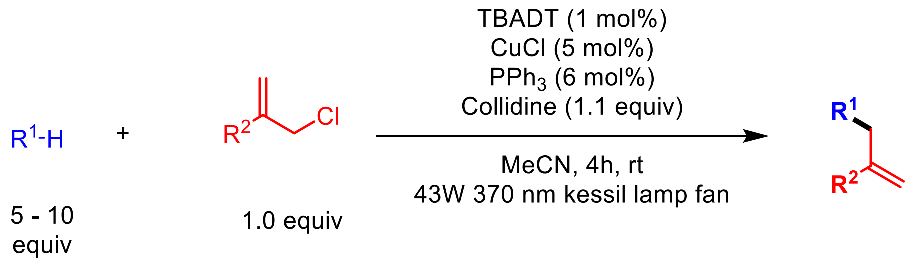

An oven-dried 8 mL vial (vial A) equipped with stirring magnetic bar was charged with TBADT (0.01 equiv, 0.005 mmol), collidine (1.1 equiv, 0.55 mmol), allylic chloride (1 equiv, 0.5 mmol), alkane (5 or 10 equiv, 2.5 or 5 mmol) and 2 mL of dry MeCN, and the vial was capped and wrapped with aluminum foil.
An oven-dried 4 mL vial (vial B) equipped with stirring magnetic bar was charged with CuCl (0.05 equiv, 0.025 mmol), triphenylphosphine (0.06 equiv, 0.03 mmol) and 1 mL of dry MeCN, and it was stirred for 15 min at RT, until a white suspension appeared.
The solution of the preformed copper catalyst was added to vial A which was previously cooled-down at 0 ºC (ice-water bath). Vial B was rinsed with 2 mL of MeCN and the solution was added to vial A. The final mixture (MeCN, 0.1 M) was bubbled with Ar for 10 min. Then the cap of the vial was sealed with parafilm, the aluminum foil removed, and the vial was placed to the direct flux of LED on the vial holder at 2 – 3 cm from the light source. In order to keep the temperature below 30 ºC, a fan was placed in front of the vials (T Max.= 30 ˚C) (See Figure S1). After 4 h, the solution was filtered through a silica plug and the residue was washed with Et_2O (20 mL). Then the crude was purified by flash column chromatography on silica gel.

5. Reaction Setup

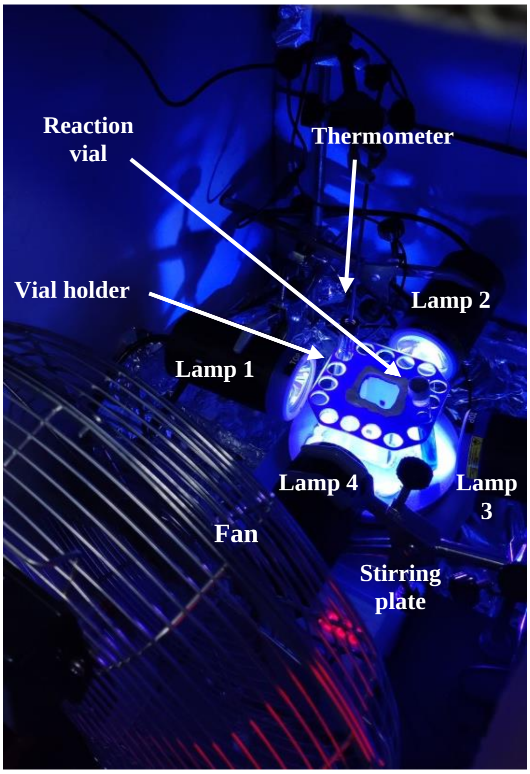

Each lamp (*43W Kessil PR160 – 370 nm*) could irradiate two reaction vials, from 2 - 3 cm distance. Temperature was monitored using the probe of the stirring plate submerged in water into a vial next to the reaction vial. A fan was employed to maintain the temperature below 30°C.

Figure S1. Reaction Setup

6. Compound characterization

(3-Cyclohexylprop-1-en-2-yl)benzene (3)

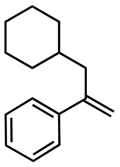

Synthesized from cyclohexane **1** and allylic chloride **2** following general procedure (4 h). Colorless oil obtained in 62% yield (62.0 mg) after flash column chromatography (hexanes).

^{1}H NMR (300 MHz, $CDCl_3$) δ 7.52 – 7.27 (m, 5H), 5.31 (d, *J* = 1.8 Hz, 1H), 5.08 – 5.03 (d, *J* = 1.8 Hz, 1H), 2.44 (dd, *J* = 7.1, 1.2 Hz, 2H), 1.82 – 1.61 (m, 5H), 1.38 (m, 1H), 1.23 – 1.12 (m, 3H), 1.02 – 0.89 (m, 2H). ^{13}C NMR (75 MHz,$CDCl_3$) δ 147.4 (C), 141.7 (C), 128.3 (2xCH), 127.3 (CH), 126.4 (2xCH), 113.5 (CH_2), 43.8 (CH_2), 35.9 (CH), 33.4 (2xCH_2), 26.7 (CH_2), 26.4 (2xCH_2). The spectroscopic signals are in accordance to reported literature.[10]

1-(3-Cyclohexylprop-1-en-2-yl)-4-methoxybenzene (4)

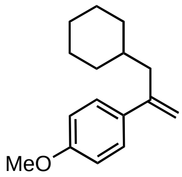

Synthesized from cyclohexane **1** and allylic chloride **49** following general procedure (8 h). Colorless oil obtained in 54% yield (62.1 mg) after flash column chromatography (hexanes/AcOEt 95:5).

^{1}H NMR (500 MHz, $CDCl_3$)) δ 7.26 (d, *J* = 8.8 Hz, 2H), 6.78 (d, *J* = 8.8 Hz, 2H), 5.11 (d, *J* = 1.8 Hz, 1H), 4.84 (d, *J* = 1.8 Hz, 1H), 3.73 (s, 3H), 2.28 (dd, *J* = 7.1, 1.1 Hz, 2H), 1.66 – 1.55 (m, 5H), 1.25 (m, 1H), 1.09 – 1.02 (m, 3H), 0.87 – 0.76 (m, 2H). ^{13}C NMR (126 MHz, $CDCl_3$) δ 159.1 (C), 146.6 (C), 134.0 (C), 127.4 (2xCH), 113.7 (2xCH), 112.0, (CH_2) 55.4 (CH_3), 43.9 (CH_2), 35.9 (CH), 33.4 (2xCH_2), 26.7 (CH_2), 26.4 (2xCH_2). The spectroscopic signals are in accordance to reported literature. [11]

1-(3-Cyclohexylprop-1-en-2-yl)-4-fluorobenzene (5)

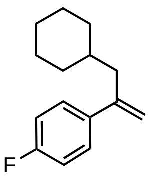

Synthesized from cyclohexane **1** and allylic chloride **50** following general procedure (4 h). Colorless oil obtained in 57% yield (61.8 mg) after flash column chromatography (hexanes).

^{1}H NMR (500 MHz, $CDCl_3$) δ 7.38 – 7.31 (m, 2H), 7.03 – 6.97 (m, 2H), 5.21 (d, *J* = 1.7 Hz, 1H), 4.99 (d, *J* = 1.7 Hz, 1H), 2.36 (dd, *J* = 7.1, 1.1 Hz, 2H), 1.74 – 1.55 (m, 5H), 1.31 (dtd, *J* = 11.0, 7.6, 3.9 Hz, 1H), 1.18 – 1.08 (m, 3H), 0.96 – 0.82 (m, 2H). **^{13}C NMR** (126 MHz, $CDCl_3$) δ 162.3 (d, *J* = 245.8 Hz) (C), 146.4 (C), 137.7 (d, *J* = 3.4 Hz) (C), 127.9 (d, *J* = 7.8 Hz) (2xCH), 115.2 (d, *J* = 21.3 Hz) (2xCH), 113.5 (CH_2), 43.9 (CH_2), 35.9 (CH), 33.4 (2xCH_2), 26.7 (CH_2), 26.3 (2xCH_2). **^{19}F NMR** (471 MHz, $CDCl_3$) δ -115.7. **HRMS** (ESI, m/z): calculated for $C_{15}H_{19}F$ [M^+]: 218.1544; found: 218.1548.

1-(*tert*-Butyl)-4-(3-cyclohexylprop-1-en-2-yl)benzene (6)

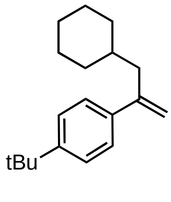

Synthesized from cyclohexane **1** and allylic chloride **51** following general procedure (4 h). Colorless oil obtained in 42% yield (53.7 mg) after flash column chromatography (hexanes).

^{1}H NMR (500 MHz, $CDCl_3$) δ 7.46 (d, *J* = 1.3 Hz, 4H), 5.39 (s, 1H), 5.08 (s, 1H), 2.50 (d, *J* = 7.1 Hz, 2H), 1.89 – 1.67 (m, 5H), 1.50 (ddt, *J* = 10.9, 7.2, 3.3 Hz, 1H), 1.45 (d, *J* = 1.4 Hz, 9H), 1.26 (dp, *J* = 12.2, 4.1 Hz, 3H), 1.02 (qd, *J* = 11.8, 3.4 Hz, 2H). **^{13}C NMR** (126 MHz, $CDCl_3$) δ 150.3 (C), 146.8 (C), 138.5 (C), 125.9 (2xCH), 125.2 (2xCH), 112.8 (CH_2), 43.7 (CH_2), 35.9 (CH), 34.6 (2xCH_2), 33.5 (3xCH_3), 31.5 (CH_2), 26.8 (CH_2), 26.4

---

[10] Corce, V.; Chamoreau, L.-M.; Derat, E.; Goddard, J.-P.; Ollivier, C.; Fensterbank, L. *Angew. Chem. Int. Ed.* **2015**, *54*, 11414-11418.

[11] Miller, Z. D.; Montgomery, J. *Org. Lett.* **2014**, *16*, 5486-5489.

(CH_2). **HRMS** (APCI, m/z): calculated for $C_{19}H_{29}$ [M^+ + H]: 257.2264; found: 257.2256.

(2-(4-(*tert*-Butyl)phenyl)allyl)cyclooctane (7)

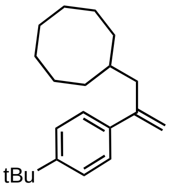

Synthesized from cyclooctane **39** (5 equiv) and allylic chloride **51** following general procedure (4 h). Colorless oil obtained in 70% yield (99.4 mg) after flash column chromatography (hexanes).

^{1}H NMR (500 MHz, $CDCl_3$) δ 7.34 (dt, *J* = 4.8, 1.3 Hz, 4H), 5.27 (s, 1H), 4.98 (s, 1H), 2.42 - 2.36 (m, 2H), 1.69 – 1.60 (m, 5H), 1.59 – 1.50 (m, 3H), 1.48 – 1.42 (m, 3H), 1.41 – 1.36 (m, 2H), 1.33 (s, 9H), 1.30 – 1.24 (m, 2H). **^{13}C NMR** (126 MHz, $CDCl_3$) δ 150.1 (C), 147.4 (C), 138.5 (C), 125.8 (2xCH), 125.1 (2xCH), 112.8 (CH_2), 43.7 (CH_2), 35.4 (CH), 34.5 (C), 31.7 (2xCH_2), 31.4 (3xCH_3), 27.5 (2xCH_2), 26.1 (CH_2), 25.2 (2xCH_2). **HRMS** (APCI, m/z): calculated for $C_{21}H_{38}$ [M^+ + H]: 285.2577; found: 285.2566.

1,3-Di-*tert*-butyl-5-(3-cyclohexylprop-1-en-2-yl)benzene (8)

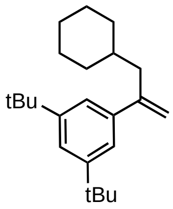

Synthesized from cyclohexane **1** and allylic chloride **52** following general procedure (4 h). Colorless oil obtained in 38% yield (59.3 mg) after flash column chromatography (hexanes).

^{1}H NMR (500 MHz, $CDCl_3$) δ 7.47 (t, *J* = 1.8 Hz, 1H), 7.37 (s, 2H), 5.40 (s, 1H), 5.12 (s, 1H), 2.52 (d, *J* = 6.9 Hz, 2H), 1.92 – 1.67 (m, 4H), 1.47 (s, 18H), 1.30 (tdd, *J* = 18.6, 13.1, 7.1 Hz, 5H), 1.12 – 0.93 (m, 3H). **^{13}C NMR** (126 MHz, $CDCl_3$) δ 150.5 (C), 148.2 (C), 140.9 (C), 121.4 (CH), 120.6 (2xCH), 112.8 (CH_2), 44.1 (CH_2), 36.1 (CH), 35.0 (C), 33.6 (2xCH_2), 31.7 (6xCH_3), 26.8 (CH_2), 26.4 (2xCH_2). **HRMS** (APCI, m/z): calculated for $C_{23}H_{27}$ [M^+ + H]: 313.2890; found: 313.2879.

(2-(3,5-Di-*tert*-butylphenyl)allyl)cyclooctane (9)

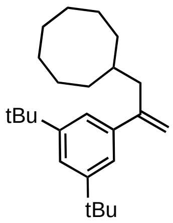

Synthesized from cyclooctane **39** (5 equiv) and allylic chloride **52** following general procedure (4 h). Colorless oil obtained in 68% yield (115.6 mg) after flash column chromatography (hexanes).

^{1}H NMR (500 MHz, $CDCl_3$) δ 7.34 (bs, 1H), 7.23 (bs, 2H), 5.27 (s, 1H), 5.01 (s, 1H), 2.46 – 2.38 (m, 2H), 1.69 – 1.59 (m, 6H), 1.58 – 1.37 (m, 7H), 1.37 – 1.32 (m, 20H). **^{13}C NMR** (126 MHz, $CDCl_3$) δ 150.4 (2C), 149.0 (C), 140.9 (C), 121.3 (CH), 120.8 (2xCH), 112.9 (CH_2), 44.6 (CH_2), 35.3 (CH_2), 35.0 (CH_2), 32.2 (CH_2), 31.7 (CH_2), 27.4 (CH_2), 26.5 (CH_2), 25.5 (CH_2). **HRMS** (APCI, m/z): calculated for $C_{25}H_{41}$ [M^+ + H]: 341.3203; found: 341.2307.

1-(3-Cyclohexylprop-1-en-2-yl)-3,5-bis(trifluoromethyl)benzene (10)

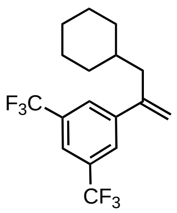

Synthesized from cyclohexane **1** and allylic chloride **53** following general procedure (4 h). Colorless oil obtained in 50% yield (84.0 mg) after flash column chromatography (hexanes).

^{1}H NMR (500 MHz, $CDCl_3$) δ 7.80 (s, 2H), 7.77 (s, 1H), 5.39 (s, 1H), 5.21 (s, 1H), 2.43 (d, *J* = 7.1, 2H), 1.74 – 1.65 (m, 4H), 1.64 – 1.60 (m, 1H), 1.34 – 1.27 (m, 1H), 1.26 - 1.23 (m, 1H), 1.21 – 1.10 (m, 2H), 0.91 (dtd, *J* = 13.7, 11.2, 3.8 Hz, 2H). **^{13}C NMR** (126 MHz, $CDCl_3$) δ 145.1 (C), 145.1 (C), 144.0 (C), 131.8 (q, *J* = 33.0 Hz) (2xC), 126.4 (d, *J* = 4.2 Hz) (2xCH), 123.6 (q, *J* = 272.7 Hz) (2xC), 121.1 (dt, *J* = 7.7, 3.8 Hz) (CH), 116.8 (CH_2), 43.3 (CH_2), 36.0 (CH), 33.3 (2xCH_2), 29.9 (CH_2), 26.6 (CH_2), 26.3 (CH_2). **^{19}F NMR** (282 MHz, $CDCl_3$) δ -62.9. **HRMS** (APCI, m/z): calculated for $C_{17}H_{19}F_6$ [M^+ + H]: 337.1385; found: 337.1390.

5-(3-Cyclohexylprop-1-en-2-yl)-1,2,3-trimethoxybenzene (11)

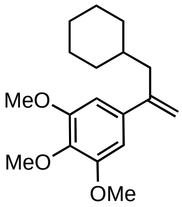

Synthesized from cyclohexane **1** and allylic chloride **54** following general procedure (4 h). Colorless oil obtained in 55% yield (79.7 mg) after flash column chromatography (hexanes/AcOEt 4:1).

^{1}H NMR (500 MHz, $CDCl_3$) δ 6.60 (s, 2H), 5.22 (d, *J* = 1.7 Hz, 1H), 4.98 (dd, *J* = 1.7 Hz, 1H), 3.87 (s, 6H), 3.86 (s, 3H), 2.34 (dd, *J* = 7.0, 1.1 Hz, 2H), 1.75 – 1.58 (m, 5H), 1.36 (ddd, *J* = 11.0, 7.3, 3.6 Hz, 1H), 1.22 – 1.09 (m, 3H), 0.90 (qd, *J* = 11.5, 2.7 Hz, 2H). **^{13}C NMR** (126 MHz, $CDCl_3$) δ 153.0 (3xC), 147.4 (C), 137.6 (C), 113.2 (CH_2), 103.8 (2xCH), 61.0 (CH_3), 56.3 (2xCH_3), 43.9 (CH_2), 36.1 (CH), 33.4 (2xCH_2), 26.7 (CH_2), 26.3 (2xCH_2). **HRMS** (APCI, m/z): calculated for $C_{18}H_{27}O_3$ [M^+ + H]: 291.1955; found: 291.1960.

1-(3-Cyclohexylprop-1-en-2-yl)naphthalene (12)

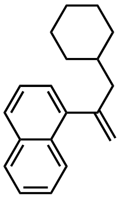

Synthesized from cyclohexane **1** and allylic chloride **55** following general procedure (8 h). Colorless oil obtained in 35% yield (43.8 mg) after flash column chromatography (hexanes).

^{1}H NMR (500 MHz, $CDCl_3$) δ 8.10 – 8.04 (m, 1H), 7.84 (dt, *J* = 6.9, 3.6 Hz, 1H), 7.76 (d, *J* = 8.2 Hz, 1H), 7.50 – 7.39 (m, 3H), 7.29 – 7.24 (m, 1H), 5.35 (s, 1H), 5.11 (s, 1H), 2.43 (d, *J* = 6.9 Hz, 2H), 1.82 – 1.74 (m, 2H), 1.71 – 1.57 (m, 3H), 1.28 (ddq, *J* = 11.0, 7.4, 3.7 Hz, 1H), 1.18 – 1.07 (m, 3H), 0.92 (qd, *J* = 11.4, 3.7 Hz, 2H). **^{13}C NMR** (126 MHz, $CDCl_3$) δ 147.5 (C), 141.7 (C), 133.9 (C), 131.4 (C), 128.4 (CH), 127.2 (CH), 126.1 (CH), 125.8 (CH), 125.7 (CH), 125.4 (CH), 125.2 (CH), 116.7 (CH_2), 46.9 (CH_2), 35.8 (CH), 33.5 (2xCH_2), 26.7 (CH_2), 26.3 (2xCH_2). **HRMS** (APCI, m/z): calculated for $C_{19}H_{23}$ [M^+ + H]: 251.1794; found: 251.1786.

1-(3-Cyclohexylprop-1-en-2-yl)-3-methoxybenzene (13)

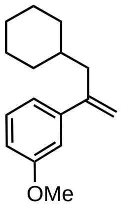

Synthesized from cyclohexane **1** and allylic chloride **56** following general procedure (8 h). Colorless oil obtained in 51% yield (58.7 mg) after flash column chromatography (hexanes/AcOEt 95:5).

^{1}H NMR (500 MHz, $CDCl_3$) δ 7.27 – 7.22 (m, 1H), 6.99 (ddd, *J* = 7.7, 1.7, 0.9 Hz, 1H), 6.94 (t, *J* = 2.1 Hz, 1H), 6.82 (ddd, *J* = 8.3, 2.6, 0.9 Hz, 1H), 5.27 (d, *J* = 1.8 Hz, 1H), 5.01 (d, *J* = 1.8, 1H), 3.83 (s, 3H), 2.38 (d, *J* = 7.1 Hz, 2H), 1.74 – 1.57 (m, 5H), 1.35 (dtq, *J* = 14.5, 7.1, 3.5 Hz, 1H), 1.20 – 1.05 (m, 3H), 0.89 (qd, *J* = 11.6, 3.5 Hz, 2H). **^{13}C NMR** (126 MHz, $CDCl_3$) δ 159.6 (C), 147.3 (C), 143.3 (C), 129.3 (CH), 119.0 (CH), 113.8 (CH_2), 112.5 (CH), 112.4 (CH), 55.4 (CH_3), 43.9 (CH_2), 35.9 (CH), 33.4 (2xCH_2), 26.7 (CH_2), 26.3 (2xCH_2). **HRMS** (APCI, m/z): calculated for $C_{16}H_{23}O$ [M^+ + H]: 231.1743; found: 231.1748.

(2-(Cyclohexylmethyl)allyl)trimethylsilane (14)

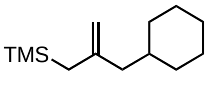

Synthesized from cyclohexane **1** and allylic chloride **58** following general procedure (8 h). Colorless oil obtained in 45% yield (44.2 mg) after flash column chromatography (hexanes). **^{1}H NMR** (500 MHz, $CDCl_3$) δ 4.51 (m, 2H), 1.82 (dd, *J* = 7.1, 1.2 Hz, 2H), 1.70 – 1.65 (m, 2H), 1.48 (d, *J* = 1.0 Hz, 2H), 1.45 – 1.35 (m, 1H), 1.29 – 1.06 (m, 5H), 0.89 – 0.77 (m, 3H), 0.00 (s, 9H). ^{13}C NMR (126 MHz, $CDCl_3$) δ 146.3, 108.3 (CH_2), 46.7 (CH_2), 35.8 (CH), 33.5 (2xCH_2), 26.8 (CH_2), 26.6 (CH_2), 26.5 (2xCH_2), -1.1 (3xCH_3). The spectroscopic signals are in accordance to reported literature.[12]

[12] Uenishi, J.; Iwamoto, T.; Ohmi, M. *Tetrahedron Lett.* **2007**, *48*, 1237-1240.

(2-Methylenehex-5-en-1-yl)cyclohexane (15)

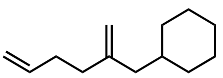

Synthesized from cyclohexane **1** and allylic chloride **59** following general procedure (8 h). Colorless oil obtained in 30% yield (26.7 mg) after flash column chromatography (hexanes).

^{1}H NMR (500 MHz, $CDCl_3$) δ 5.83 (ddt, *J* = 16.9, 10.3, 6.5 Hz, 1H), 5.09 – 4.86 (m, 2H), 4.80 – 4.58 (m, 2H), 2.17 (dq, *J* = 21.1, 7.3 Hz, 4H), 2.07 (dd, *J* = 9.2, 6.1 Hz, 2H), 1.91 (d, *J* = 7.1 Hz, 1H), 1.71 – 1.64 (m, 4H), 1.46 – 1.36 (m, 1H), 1.25 – 1.12 (m, 3H), 0.85 (tt, *J* = 15.2, 5.1 Hz, 2H). **^{13}C NMR** (126 MHz, $CDCl_3$) δ 147.7 (C), 138.8 (CH), 114.5 (CH_2), 110.4 (CH_2), 44.7 (CH_2), 35.7 (CH), 35.3 (CH_2), 33.5 (CH_2), 32.2 (CH_2), 29.9 (CH_2), 26.8 (CH_2), 26.5 (2xCH_2). **HRMS** (ESI, m/z): calculated for $C_{13}H_{23}$ [M^+ + H]: 179.1794; found: 179.1799.

(2-Chloroallyl)cyclooctane (16)

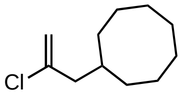

Synthesized from cyclooctane **39** (5 equiv) and allylic chloride **57** following general procedure (4 h). Colorless oil obtained in 70% yield (52.5 mg) after flash column chromatography (hexanes).

^{1}H NMR (500 MHz, $CDCl_3$) δ 5.16 (d, *J* = 1.0 Hz, 1H), 5.09 (q, *J* = 1.0 Hz, 1H), 2.21 (dd, *J* = 7.2, 1.0 Hz, 2H), 1.93 (dddt, *J* = 8.8, 7.3, 3.5, 2.0 Hz, 1H), 1.65 – 1.49 (m, 10H), 1.30 – 1.22 (m, 4H). **^{13}C NMR** (126 MHz, $CDCl_3$) δ 142.6 (C), 113.2 (CH_2), 47.6 (CH_2), 34.7 (CH), 31.7 (2xCH_2), 27.3 (2xCH_2), 26.5 (CH_2), 25.5 (2xCH_2). **HRMS** (APCI m/z): calculated for $C_{11}H_{20}Cl$ [M^+ + H - Cl]: 151.1487 ; found: 151.1482.

(2-(Chloromethyl)allyl)cyclohexane (17)

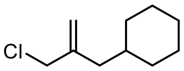

Synthesized from cyclohexane **1** and allylic chloride **60** following general procedure (4 h). Colorless oil obtained **17** in 30% yield (25.7 mg) after flash column chromatography (hexanes). **^{1}H NMR** (500 MHz, $CDCl_3$) δ 5.08 (d, *J* = 1.3 Hz, 1H), 4.85 (d, *J* = 1.3 Hz, 1H), 3.95 (d, *J* = 1.0 Hz, 2H), 2.00 (d, *J* = 7.2 Hz, 2H), 1.68 – 1.53 (m, 5H), 1.42 – 1.31 (m, 1H), 1.21 – 1.03 (m, 3H), 0.87 – 0.76 (m, 2H). **^{13}C NMR** (126 MHz, $CDCl_3$) δ 143.8 (C), 115.3 (CH_2), 48.3 (CH_2), 41.2 (CH_2), 35.4 (CH), 33.3 (2xCH_2), 26.5 (CH_2), 26.2 (2xCH_2). **HRMS** (ESI, m/z): calculated for $C_{10}H_{17}Cl$ **17** [M^+]: 172.1019, found: 172.1015.

(3-Cyclopentylprop-1-en-2-yl)benzene (18)

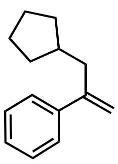

Synthesized from cyclopentane **37** and allylic chloride **2** following general procedure (4 h). Colorless oil obtained in 53% yield (49.3 mg) after flash column chromatography (hexanes). **^{1}H NMR** (500 MHz, $CDCl_3$) δ 7.36 – 7.32 (m, 2H), 7.26 (m, 2H), 7.22 – 7.18 (m, 1H), 5.17 (s, 1H), 4.98 (s, 1H), 2.44 (d, *J* = 7.4 Hz, 2H), 1.85 (m, 1H), 1.65 – 1.58 (m, 2H), 1.57 – 1.50 (m, 2H), 1.43 – 1.37 (m, 2H), 1.10 (m, 2H). **^{13}C NMR** (126 MHz, $CDCl_3$) δ 148.6 (C), 141.8 (C), 128.3 (2xCH), 127.3 (CH), 126.4 (2xCH_2), 112.9 (CH_2), 42.2 (CH_2), 38.4 (CH), 32.6 (2xCH_2), 25.2 (2xCH_2). The spectroscopic signals are in accordance to reported literature.[13]

[13] Coppola, G.; Oliva, M.; Van der Eycken, E. V.; Sharma, U. K. *ACS Catal.* **2021**, *11*, 10862-10870.

(2-Phenylallyl)cycloheptane (19)

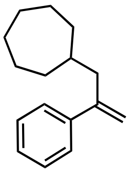

Synthesized from cycloheptane **38** and allylic chloride **2** following general procedure (4 h). Colorless oil obtained in 59% yield (63.1 mg) after flash column chromatography (hexanes). **^{1}H NMR (**500 MHz, $CDCl_3$) δ 7.37 – 7.30 (m, 2H), 7.29 – 7.23 (m, 2H), 7.22 – 7.17 (m, 1H), 5.20 (s, 1H), 4.95 (s, 1H), 2.35 (dd, *J* = 7.2, 1.2 Hz, 2H), 1.64 (m, 2H), 1.59 – 1.35 (m, 7H), 1.32 – 1.22 (m, 2H), 1.16 – 1.07 (m, 2H). **^{13}C NMR** (126 MHz, $CDCl_3$) δ 148.0 (C), 141.7 (C), 128.3 (2xCH), 127.3 (CH), 126.4 (2xCH), 113.7 (CH_2), 44.2 (CH_2), 37.3 (CH), 34.5 (2xCH_2), 28.7 (2xCH_2), 26.4 (2xCH_2). The spectroscopic signals are in accordance to reported literature.[14]

(2-Phenylallyl)cyclooctane (20)

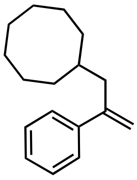

Synthesized from cyclooctane **39** (5 equiv) and allylic chloride **2** following general procedure (4 h). Colorless oil obtained in 65% yield (74.2 mg) after flash column chromatography (hexanes). **^{1}H NMR** (500 MHz, $CDCl_3$) δ 7.35 – 7.31 (m, 2H), 7.29 – 7.24 (m, 2H), 7.23 – 7.18 (m, 1H), 5.21 (s, 1H), 4.97 (s, 1H), 2.36 (dd, *J* = 7.0, 1.3 Hz, 2H), 1.61 – 1.53 (m, 5H), 1.51 – 1.17 (m, 10H). **^{13}C NMR** (126 MHz, $CDCl_3$) δ 148.1 (C), 141.7 (C), 128.3 (2xCH), 127.3 (CH), 126.5 (2xCH), 113.7 (CH_2), 44.1 (CH_2), 35.4 (CH), 31.9 (2xCH_2), 27.5 (2xCH_2), 26.4 (CH_2), 25.4 (2xCH_2). The spectroscopic signals are in accordance to reported literature.[14]

(4-Methylhept-1-en-2-yl)benzene (21a) and (4-ethylhex-1-en-2-yl)benzene (21b) and oct-1-en-2-ylbenzene (21c)

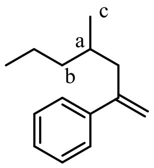

Synthesized from *n*-pentane **41** and allylic chloride **2** following general procedure (16 h). Colorless oil obtained in 38% yield (35.7 mg) as a 6:3:1 mixture of regioisomers (**a**:**b**:**c** = 6:3:1; 60% selectivity) after flash column chromatography (hexanes). **^{1}H NMR** (500 MHz, $CDCl_3$) δ 7.33 – 7.30 (m, 2H **a** + 2H **b** + 2H **c**), 7.26 – 7.21 (m, 2H **a** + 2H **b** + 2H **c**), 7.20 – 7.15 (m, 1H **a** + 1H **b** + 1H **c**), 5.18 (d, *J* = 1.5 Hz, 1H **c** + bs, 1H **a** + bs, 1H **b**), 4.98 (d, *J* = 1.5 Hz, 1H **c**), 4.96 (d, *J* = 1.5 Hz, 1H **b**), 4.95 (d, *J* = 1.5 Hz, 1H **a**), 2.51 (ddd, *J* = 14.0, 7, 1.0 Hz, 1H **a**), 2.42 (t, *J* = 7.6, 1.0 Hz, 2H **c**), 2.36 (m, 2H **b**), 2.14 (ddd, *J* = 14.0, 7, 1.0 Hz, 1H **a**), 1.48 – 1.34 (m, 1H **a** + 1H **b**), 1.30 – 1.12 (m, 4H **a** + 4H **b**), 1.07 – 1.0 (m, 6H **c**), 0.82 – 0.79 (m, 3H **c**), 0.79 – 0.71 (m, 6H **a** + 6H **b**). **^{13}C NMR** (126 MHz, $CDCl_3$) δ 147.6 (C **c**), 146.7 (C **b**), 146.6 (C **a**), 140.3 (C **c** + C **b**), 140.2 (C **a**), 127.0 (3xCH **a**), 126.9 (3xCH **b**), 125.9 (2xCH **c** + 2xCH **a**), 125.1 (2xCH **b**), 124.9 (3xCH **c**), 112.4 (CH_2 **b**), 112.3 (CH_2 **a**), 110.7 (CH_2 **c**), 42.1 (CH_2 **a**), 38.4 (CH_2 **b**), 37.9 (CH_2 **a**), 37.0 (CH **b**), 34.1 (CH_2 **c**), 30.4 (CH_2 **c**), 29.5 (CH **a**), 27.8 (CH_2 **b**), 27.0 (CH_2 **b**), 23.7 (CH_2 **a**), 21.4 (CH_2 **c**), 18.9 (2xCH_2 **c**), 18.1 (CH_3 **a**), 13.0 (CH_3 **a**), 12.9 (CH_3, **c**), 9.3 (2xCH_3 **b**). **HRMS** (APCI, m/z): calculated for $C_{14}H_{21}$ [M^+ + H]: 189.1638; found: 189.1635.

(4-Methyloct-1-en-2-yl)benzene (22a) and (4-ethylhept-1-en-2-yl)benzene (22b) and non-1-en-2-ylbenzene (22c)

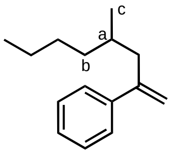

Synthesized from *n*-hexane **42** and allylic chloride **2** following general procedure (16 h). Colorless oil obtained in 40% yield (40.4 mg) as a 6:3:1 mixture of regioisomers (**a**:**b**:**c** = 6:3:1; 60% selectivity) after flash column chromatography (hexanes). **^{1}H NMR** (500 MHz, $CDCl_3$) δ 7.34 – 7.28 (m, 2H **a** + 2H **b** +2H **c**),

[14] Liang, L.; Guo, G.; Li, C.; Wang, S.-L.; Wang, Y.-H.; Guo, H.-M.; Niu, H.-Y. *Org. Lett.* **2021**, *23*, 8575-8579.

7.26 – 7.21 (m, 2H **a** + 2H **b** + 2H **c**), 7.20 – 7.14 (m, 1H **a** + 1H **b** + 1H **c**), 5.18 (d, J = 1.9 Hz, 1H **a** + 1H **b** +1H **c**), 4.97 (m, 1H **c**), 4.95 (m, 1H **a** + 1H **b**), 2.51 (ddd, *J* = 14.0, 6.0, 1.4 Hz, 1H **b**), 2.41 (t, *J* = 7.6 Hz, 2H **c**), 2.36 (d, J = 7.1 Hz, 2H **a**), 2.14 (ddd, *J* = 14.0, 6.0, 1.4 Hz, 1H **b**), 1.44 – 1.33 (m, 1H **a** + 1H **b**), 1.32 – 1.09 (m, 6H **a** + 6H **b**), 1.07 – 1.04 (m, 10H **c**), 0.81 – 0.72 (m, 3H **c** + 6H **a** + 6H **b**). **^{13}C NMR** (126 MHz, $CDCl_3$) δ 147.6 (C **c**), 146.8 (C **b**), 146.6 (C **a**), 140.3 (C **b**), 140.2 (C **c** + C **a**), 126.9 (2xCH **c** + 2xCH **a** + 2xCH **b**), 125.9 (CH **c** + CH **a** + CH **b**), 125.1 (2xCH **b**), 125.0 (2xCH **a**), 124.8 (2xCH **c**), 112.4 (CH_2 **b**), 112.3 (CH_2 **a**), 110.7 (CH_2 **c**), 42.2 (CH_2 **a**), 38.8 (CH_2 **b**), 35.4 (CH **b**), 35.3 (CH_2 **a**), 34.2 (CH_2 **c**), 33.9 (CH_2 **b**), 30.6 (CH_2 **c**), 29.7 (CH **a**), 28.1 (CH_2 **c**), 27.9 (CH_2 **a**), 27.8 (CH_2 **c**), 27.1 (CH_2 **c**), 24.3 (CH_2 **b**), 21.7 (CH_2 **a**), 21.4 (CH_2 **c**), 18.7 (CH_3 **a**), 18.3 (CH_2 **b**), 13.3 (CH_3 **c**), 12.8 (CH_3 **a**), 9.3 (2xCH_3 **b**). **HRMS** (APCI, m/z): calculated for $C_{15}H_{23}$ [M^+ + H]: 203.1794; found: 203.1787.

(4,4-Dimethylhex-1-en-2-yl)benzene (23a) and (4,5-dimethylhex-1-en-2-yl)benzene (23b)

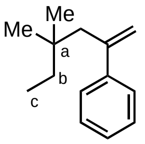

Synthesized from isopentane **43** and allylic chloride **2** following general procedure (8 h). Colorless oil obtained in 27% yield (25.4 mg) as a 6.3:2:1mixture of regioisomers (**a**:**b**:**c** = 6.3:2:1; 70% selectivity) after flash column chromatography (hexanes). NMR data is only given for major isomers **a** and **b**.

^{1}H NMR (500 MHz, $CDCl_3$) δ 7.45 – 7.21 (m, 5H **a** + 5H **b**), 5.27 (d, *J* =1.7 Hz, 1H **b**), 5.24 (d, *J* = 2.1 Hz, 1H **a**), 5.04 (d, *J* = 1.4 Hz, 1H **b**), 5.03 (m, 1H **a**), 2.69 (ddd, *J* = 13.9, 5.0, 1.4 Hz, 1H **b**), 2.47 (s, 2H **a**), 2.17 (ddd, *J* = 14.0, 9.5, 0.9 Hz, 1H **b**), 1.61 (m, 1H **b**),), 1.51 – 1.38 (m, 1H **b**), 1.20 (q, *J* = 7.5 Hz, 2H **a**), 0.89 – 0.84 (m, 6H **b**), 0.79 (t, *J* = 7.5 Hz, 3H **a**), 0.77 (d, *J* =6.8 Hz, 3H **b**), 0.73 (s, 6H **a**). **^{13}C NMR** (126 MHz, $CDCl_3$) δ 148.2 (C **b**), 147.7 (C **a**), 144.2 (C **a**), 141.6 (C **b**), 128.4 (2xCH **b**), 128.2 (2xCH **a**), 127.3 (CH **b**), 127.0 (CH **a**), 126.7 (2xCH **a**), 126.5 (2xCH **2b**), 116.6 (CH_2 **a**), 113.7 (CH_2 **b**), 46.9 (CH_2 **a**), 40.5 (CH_2 **b**), 36.6 (CH **b**), 35.0 (CH_2 **a**), 34.4 (C, **a**), 32.0 (CH **b**), 27.1 (2xCH_3 **a**), 20.3 (CH_3 **b**), 18.0 (CH_3 **b**), 15.0 (CH_3 **b**), 8.6 (CH_3 **a**). **HRMS** (APCI, m/z): calculated for $C_{14}H_{21}$ [M^+ + H]: 189.1638; found: 189.1635.

3-(2-Phenylallyl)cyclopentan-1-one (24)

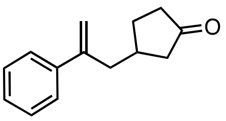

Synthesized from cyclopentanone **40** and allylic chloride **2** following general procedure (4 h). Colorless oil obtained in 50% yield (50.0 mg) after flash column chromatography (hexanes).

^{1}H NMR (500 MHz, $CDCl_3$) δ 7.41 – 7.37 (m, 2H), 7.36 – 7.31 (m, 2H), 7.31 – 7.26 (m, 1H), 5.29 (d, *J* = 1.3 Hz, 1H), 5.08 (q, *J* = 1.3 Hz, 1H), 2.70 – 2.59 (m, 2H), 2.34 – 2.23 (m, 3H), 2.15 – 2.04 (m, 2H), 1.91 – 1.81 (m, 1H), 1.64 – 1.53 (m, 1H). **^{13}C NMR** (126 MHz, $CDCl_3$) δ 219.4 (C),146.9 (C), 140.9 (C), 128.5 (2xCH), 127.7 (CH), 126.3 (2xCH), 114.0 (CH_2), 45.0 (CH_2), 41.7 (CH_2), 38.4 (CH_2), 35.5 (CH), 29.3 (CH_2). **HRMS** (APCI, m/z): calculated for $C_{14}H_{17}O$ [M^+ + H]: 201.1274; found: 201.1274.

2-(2-Phenylallyl)tetrahydrofuran (25)

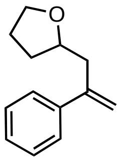

Synthesized from tetrahydrofuran **44** and allylic chloride **2** following general procedure (4 h). Colorless oil obtained in 65% yield (61.3 mg) after flash column chromatography (hexanes/AcOEt 4:1). **^{1}H NMR** (300 MHz, $CDCl_3$) δ 7.58 – 7.19 (m, 5H), 5.36 (s, 1H), 5.18 (s, 1H), 4.01 – 3.85 (m, 2H), 3.71 (q, *J* = 7.9, 5.7 Hz, 1H), 2.91 (dd, *J* = 14.2, 6.5 Hz, 1H), 2.62 (dd, *J* = 14.2, 6.9 Hz, 1H), 2.01 – 1.73 (m, 3H), 1.61 – 1.47 (m, 1H). The spectroscopic signals are in accordance to reported literature.[15]

[15] Zhang, J.; Li, Y.; Xu, R.; Chen, Y. *Angew. Chem. Int. Ed.* **2017**, *56*, 12619-12623.

2-(2-Phenylallyl)-1,4-dioxane (26)

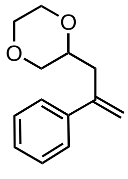

Synthesized from dioxane **47** (5 equiv) and allylic chloride **2** following general procedure (4 h). Colorless oil obtained in 45% yield (45.9 mg) after flash column chromatography (hexanes/AcOEt 4:1). **^{1}H NMR** (300 MHz, $CDCl_3$) 7.50 – 7.15 (m, 4 H), 5.36 (d, *J* = 1.4 Hz, 1H), 5.15 (q, *J* = 1.2 Hz, 1 H), 3.86 – 3.46 (m, 5 H), 3.29 (dd, *J* = 11.4 Hz, 9.8 Hz, 1 H), 2.76 (ddd, *J* = 14.4 Hz, 6.5 Hz, 1.2 Hz, 1 H), 2.52 (ddd, *J* = 14.5 Hz, 7.0 Hz, 1.0 Hz, 1 H). The spectroscopic signals are in accordance to reported literature.[16]

2-(2-Phenylallyl)tetrahydrothiophene (27)

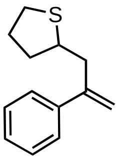

Synthesized from tetrahydrotiophene **48** (5 equiv) and allylic chloride **2** following general procedure (4 h). Colorless oil obtained in 70% yield (71.4 mg) as a 10:1 mixture of regioisomers (91% selectivity) after flash column chromatography (hexanes to 5% AcOEt). NMR data is only given for the major regioisomer.

^{1}H NMR (500 MHz, $CDCl_3$) δ 7.42 – 7.37 (m, 2H), 7.33 (ddd, *J* = 7.8, 6.5, 1.4 Hz, 2H), 7.28 (dd, *J* = 6.9, 1.6 Hz, 1H), 5.30 (d, *J* = 1.4 Hz, 1H), 5.13 (t, *J* = 1.4 Hz, 1H), 3.50 – 3.37 (m, 1H), 2.91 (ddd, *J* = 9.9, 8.0, 6.3 Hz, 1H), 2.85 – 2.78 (m, 3H), 2.05 (ddq, *J* = 32.7, 17.7, 5.5 Hz, 2H), 1.90 – 1.80 (m, 1H), 1.61 (tdd, *J* = 8.9, 7.3, 5.1 Hz, 1H). **^{13}C NMR** (126 MHz, $CDCl_3$) δ 147.2 (C), 140.9 (C), 128.5 (2xCH), 127.7 (CH), 126.4 (2xCH), 114.1 (CH_2), 47.1 (CH), 43.7 (CH_2), 36.9 (CH_2), 32.5 (CH_2), 30.3 (CH_2). **HRMS** (APCI, m/z): calculated for $C_{13}H_{17}S$ [M^+ + H]: 205.1045; found: 205.1038.

***tert*-Butyl (*S*)-2-(2-phenylallyl)morpholine-4-carboxylate (28a) and *tert*-butyl 3-(2-phenylallyl)morpholine-4-carboxylate (28b)**

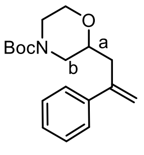

Synthesized from *N*-Boc morpholine **46** (5 equiv) and allylic chloride **2** following general procedure (4 h). Colorless oil obtained in 50% yield (75.8 mg) as a 1.5:1 mixture of regioisomers (60% selectivity) after flash column chromatography (gradient: hexanes/AcOEt 100% to 80%). **^{1}H NMR** (500 MHz, $CDCl_3$) δ 7.54 – 7.44 (m, 2H **a**), 7.42 – 7.38 (m, 2H **b**), 7.38 – 7.31 (m, 2H **a** + 2H **b**), 7.31 – 7.24 (m, 1H **a** + 1H **b**), 5.43 (d, *J* = 1.3 Hz, 1H **a**), 5.35 (d, *J* = 1.4 Hz, 1H **b**), 5.18 (s, 1H **a**), 5.18 – 5.14 (m, 1H **b**), 3.98 (s, 1H **b**), 3.93 – 3.72 (m, 3H **a** + 2H **b**), 3.48 – 3.36 (m, 3H **a**), 3.20 (td, *J* = 13.0, 3.8 Hz, 2H **b**), 3.00 (dd, *J* = 13.7, 9.7 Hz, 2H **b**), 2.92 (d, *J* = 13.2 Hz, 1H **a**), 2.81 (dd, *J* = 13.6, 5.9 Hz, 2H **a**), 2.59 (dt, *J* = 15.6, 7.7 Hz, 2H **b**), 1.42 (s, 9H **b**) , 1.40 (s, 9H **a**). **^{13}C NMR** (126 MHz, $CDCl_3$) δ 154.6 (C **b**), 154.6 (C **a**), 144.5 (C **a**), 144.0 (C **b**), 140.6 (C **b**), 140.0 (C **a**), 128.4 (2xCH **a**), 128.4 (2xCH **b**), 127.7 (CH **a**), 127.6 (CH **b**), 126.2 (2xCH **b**), 126.1 (2xCH **a**), 115.6 (CH_2 **b**), 115.3 (CH_2 **a**), 80.0 (C **b**), 79.9 (C **a**), 73.8 (CH **a**), 67.5 (CH_2 **b**), 66.9 (CH_2 **a**), 66.5 (CH_2 **a**), 50.1 (CH **b**), 39.5 (CH_2 **a**), 39.0 (CH_2 **b**), 34.2 (2xCH_2 **b**), 29.7 (CH_2 **a**) 28.4 (3xCH_3 **b**), 28.3 (3xCH_3 **a**). **HRMS** (APCI, m/z): calculated for $C_{14}H_{18}NO_3$ [M^+ + H -C_4H_9(*t*Bu)]: 248.1281; found: 248.1272.

[16] Patil, S. V.; Tanko, J. M. *Tetrahedron* **2016**, *72*, 7849-7858.

(1*R,4*S**,5*S**)-5-(2-(3-Methoxyphenyl)allyl)-1,3,3-trimethyl-2-oxabicyclo[2.2.2]octane (31a) and (1*R**,4*S**,6*S**)-6-(2-(3-methoxyphenyl)allyl)-1,3,3-trimethyl-2-oxabicyclo[2.2.2]octane (31b)**

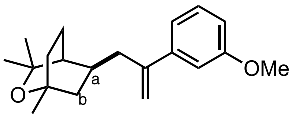

Synthesized from *Eucalyptol* **61** (5 equiv) and allylic chloride **56** following general procedure (16 h). Colorless oil obtained in 26% yield (39 mg) as an inseparable 1.4:1 mixture of regioisomers (58% selectivity) after flash column chromatography (gradient up to 4%: hexanes/AcOEt). **^{1}H NMR** (500 MHz, $CDCl_3$) δ 7.24 (t, *J* = 7.9 Hz, 1H **a** + 1H **b**), 6.96 – 6.81 (m, 3H **a** + 3H **b**), 5.31 (s, 1H **a**), 5.28 (s, 1H **a**), 5.09 (s, 1H **b**), 5.08 (s, 1H **b**), 3.81 (s, 3H **a** + 3H **b**), 2.79 (d, *J* = 6.7 Hz, 2H **b**), 2.56 – 2.46 (m, 3H **a**), 2.33 (m, 1H **b**), 2.08 – 1.95 (m, 4H **b**), 1.90 – 1.68 (m, 7H **a**), 1.66 – 1.59 (m, 3H **b**), 1.45 – 1.40 (m, 3H **b**), 1.28 – 1.21 (m, 6H **b**), 1.11 (s, 3H **a**), 1.06 – 1.01 (m, 6H **a**). **^{13}C NMR** (126 MHz, $CDCl_3$) δ 159.5 (C **a**), 159.4 (C **b**), 147.1 (C **a**), 146.6 (C **b**), 142.8 (C **a**), 142.7 (C **b**), 129.1 (CH **a** + CH **b**), 118.7 (CH **a**), 118.6 (CH **b**), 113.8 (CH_2 **b**), 113.4 (CH_2 **a**), 112.4 (CH **b**), 112.3 (CH **a**), 112.2 (CH **a**), 112.1 (CH **b**), 74.2 (C **a**), 73.0 (C **b**), 72.9 (C **b**), 70.4 (C **a**), 55.1 (CH_3 **a**), 55.0 (CH_3 **b**), 40.9 (CH_2 **a**), (CH_3 **b**), 39.8 (CH_2 **b**), 38.9 (CH_2 **a**), 38.4 (CH **b**), 36.5 (CH **a** + CH_3 **b**), 33.6 (CH **a**), 31.5 (CH_2 **a**), 29.8 (CH_2 **b**), 28.9 (CH_3 **a**), 28.8 (CH_3 **b**), 28.5 (CH **b**), 28.2 (CH_3 **b**), 27.4 (CH_3 **a**), (CH_3 **b**), 27.3 (CH_2 **b**), 25.4 (CH_3 **a**), 22.6 (CH_2 **b**), 15.9 (CH_2 **a**). **HRMS** (APCI, m/z): calculated for $C_{20}H_{29}O_2$ [M^+ + H]: 301.2162; found: 301.2169.

(1*S,4*S**,5*R**)-2,2-Dimethyl-3-methylene-5-(2-phenylallyl)bicyclo[2.2.1]heptane (32a) and (1*R**,4*R**,6*R**)-2,2-Dimethyl-3-methylene-6-(2-phenylallyl)bicyclo[2.2.1]heptane (32b)**

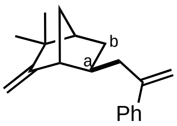

Synthesized from *Camphene* **62** (5 equiv) and allylic chloride **2** following general procedure (16 h). Colorless oil obtained in 50% yield (63 mg) as a 1.3:1 mixture of regioisomers (55% selectivity) after flash column chromatography (hexanes).

^{1}H NMR (500 MHz, $CDCl_3$) δ 7.38 (ddq, *J* = 6.6, 3.3, 1.9 Hz, 2H **a** + 2H **b**), 7.32 (t, *J* = 7.7 Hz, 2H **a** + 2H **b**), 7.28 – 7.24 (m, 1H **a** + 1H **b**), 5.28 (d, *J* = 1.4 Hz, 1H **a**), 5.26 (d, *J* = 1.4 Hz, 1H **b**), 5.05 (d, *J* = 1.4 Hz, 1H **b**), 5.04 (d, *J* = 1.4 Hz, 1H **a**), 4.71 (s, 1H **b**), 4.67 (s, 1H **a**), 4.49 (s, 1H **b**), 4.46 (s, 1H **a**), 2.68 – 2.64 (m, 1H **b**), 2.56 – 2.48 (m, 2H **a**), 2.45 (td, *J* = 7.7, 1.3 Hz, 2H **b**), 2.36 (ddd, *J* = 14.8, 7.3, 1.2 Hz, 1H **a**), 2.13 (qd, *J* = 7.9, 2.7 Hz, 1H **a**), 1.92 – 1.83 (m, 1H **a** + 2H **b**), 1.74 – 1.67 (m, 2H **b**), 1.62 (tdd, *J* = 8.6, 3.3, 1.6 Hz, 1H **a**), 1.45 – 1.37 (m, 1H **a** + 1H **b**), 1.32 – 1.21 (m, 2H **a +** 1H **b**), 1.03 (s, 3H **a**), 1.02 (s, 3H **b**), 0.99 (s, 3H **b**), 0.91 (s, 3H **a**). **^{13}C NMR** (126 MHz, $CDCl_3$) δ 166.0 (C **a**), 165.8 (C **b**), 148.0 (C **b**), 147.7 (C **a**), 142.0 (C **a**), 141.9 (C **b**), 128.4 (2xCH **a**), 128.3 (2xCH **b**), 127.4 (CH **a** + CH **b**), 126.4 (2xCH **b**), 126.4 (2xCH **a**), 113.3 (CH_2 **a**), 113.1 (CH_2 **b**), 99.8 (CH_2 **b**), 99.4 (CH_2 **a**), 52.5 (CH **b**), 51.6 (CH **a**), 48.7 (CH **a**), 47.4 (CH **b**), 42.5 (CH_2 **b**), 42.3 (C **b**), 42.1 (CH_2 **a**), 41.6 (C **a**), 39.2 (CH **b**), 37.1 (CH_2 **a**), 34.4 (CH_2 **a**), 34.2 (CH_2 **b**), 33.1 (CH **a**), 29.9 (CH_2 **b**), 29.8 (CH_3 **b**), 29.5 (CH_3 **a**), 25.8 (CH_3 **b**), 25.2 (CH_3 **a**). **HRMS** (APCI, m/z): calculated for $C_{19}H_{25}$ [M^+ + H]: 253.1951; found: 253.1952.

(1*R,4*R**,5*R**)-5-(2-(3-Methoxyphenyl)allyl)-1,3,3-trimethylbicyclo[2.2.1]heptan-2-one (33)**

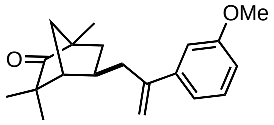

Synthesized from *Fenchone* **63** (5 equiv) and allylic chloride **56** following general procedure (16 h). Colorless oil obtained in 31% yield (46.2 mg) as a mixture of isomers 1.3:1 (56% selectivity). The major isomer was isolated pure after flash column chromatography (gradient up to 3%: hexanes/AcOEt).

^{1}H NMR (500 MHz, $CDCl_3$) δ 7.22 (t, *J* = 7.9 Hz, 1H), 6.93 (ddd, *J* = 7.6, 1.7, 0.9 Hz, 1H), 6.88 (dd, *J* = 2.6, 1.6 Hz, 1H), 6.79 (ddd, *J* = 8.3, 2.6, 0.9 Hz, 1H), 5.27 (d, *J* = 1.8 Hz, 1H), 5.04 (dd, *J* = 1.8, 0.9 Hz, 1H), 3.81 (s, 3H), 2.67 (dd, *J* = 13.7, 0.9 Hz, 1H), 2.57 (dd, *J* = 13.7, 0.8 Hz, 1H), 2.18 – 2.10 (m, 2H), 1.87 (d, *J* = 1.5 Hz, 2H), 1.60 – 1.54 (m, 2H), 1.53 – 1.47 (m, 1H), 1.03 (s, 3H), 0.99 (s, 3H), 0.85 (s, 3H). **^{13}C NMR** (126 MHz, $CDCl_3$) δ 159.6 (C), 146.3 (C), 144.8 (C), 129.3 (CH), 119.1 (CH), 117.6 (CH_2), 112.5 (CH), 112.5 (CH), 60.2 (CH), 55.2 (CH_3), 47.5 (CH_2), 47.1 (C), 46.9 (CH), 40.3 (C), 37.8 (CH_2), 34.0 (CH_2), 25.2 (CH_3), 24.4 (CH_3), 20.8 (CH_3). **HRMS** (APCI, m/z): calculated for $C_{20}H_{27}O_2$ [M^+ + H]: 299.2006; found: 299.2014.

7. Procedure for the synthesis of 16 at 1.5 mmol scale

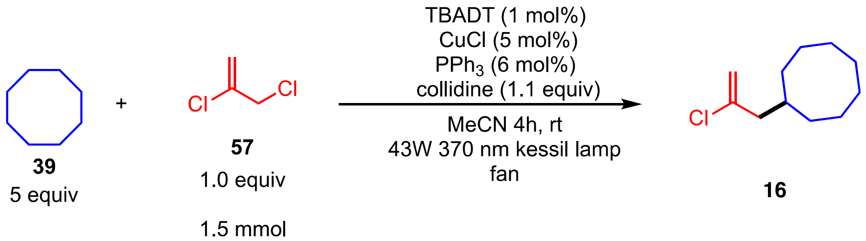

An oven-dried 20 mL vial (vial A) equipped with stirring magnetic bar was charged with TBADT (0.01 equiv, 0.015 mmol), collidine (1.1 equiv, 1.65 mmol), allylic chloride **57** (1 equiv, 1.5 mmol), cyclooctane **39** (5 equiv, 7.5 mmol) and 6 mL of dry MeCN, and the vial was capped and wrapped with aluminum foil.

An oven-dried 4 mL vial (vial B) equipped with stirring magnetic bar was charged with CuCl (0.05 equiv, 0.033 mmol), triphenylphosphine (0.06 equiv, 0.09 mmol) and 3 mL of dry MeCN, and it was stirred for 15 min at RT, until a white suspension appeared.

The solution of the preformed copper catalyst was added to vial A which was previously cooled-down at 0 ºC (ice-water bath). Vial B was rinsed with 6 mL of MeCN and the solution was added to vial A. The final mixture (MeCN, 0.1 M) was bubbled with Ar for 10 min. Then the cap of the vial was sealed with parafilm, the aluminum foil removed, and the vial was placed to the direct flux of LED on the vial holder at 2 – 3 cm from the light source. In order to keep the temperature below 30 ºC, a fan was placed in front of the vials (T Max.= 30 ˚C) (See Figure S1). After 4 h, the solution was filtered through a silica plug and the residue was washed with Et_2O (60 mL). Then the crude was purified by flash column chromatography on silica gel using hexanes to afford **16** as colorless oil (124 mg, 55% yield).

8. Optimization Studies

8.1 Evaluation of different transition metal co-catalysts

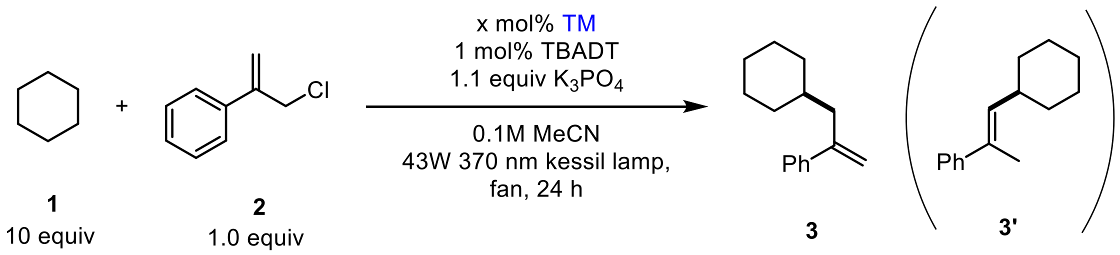

Entry[a]	TM (x mol%)	Conversion (%)[b]	3 (%)[b]
1	-	30	10
2	$Ni(dtbpy)Br_2$ (5)	100	48
3	$Ni(dtbpy)Cl_2$ (5)	82	35
4	$Ni(PyrOx)Br_2$ (5)	100	17
5	$Ni(BisOx)Br_2$ (5)	100	23
6	$Ni(COD)_2$ (5)	58	22
7	$PdCl_2$ (5)	100	15
8	$ZnBr_2$ (20)	47	9[c]
9	$FeCl_2$ (20)	43	29[c]
10	$CoCl_2$ (20)	50	35
11	CuI (5)	69	32
12	$Cu(MeCN)_4PF_6$ (5)	60	36
13	$Cu(OTf)_2$ (5)	58	35
14	$CuCl_2$ (5)	73	32
15	CuCl (5)	62	37

Table S1. a) Conditions: 0.5 mmol of **2**, 5 mL of MeCN. b) Yield and conversion were determined by ^{1}H-NMR analysis using 1,3,5-trimethoxy benzene as internal standard. c) Obtained as a 4:1 mixture of **3**:**3'**. TM = Transition Metal. dtbpy = 4,4'-di-tert-butyl-2,2'-bipyridine. dtbpy = 4,4'-di-tert-butyl-2,2'-bipyridine. PyrOx = 2-(pyridin-2-yl)-4,5-dihydrooxazole. BisOx = 4,4',5,5'-tetrahydro-2,2'-bioxazole. COD = (1*Z*,5*Z*)-cycloocta-1,5-diene.

8.2. Evaluation of the leaving group

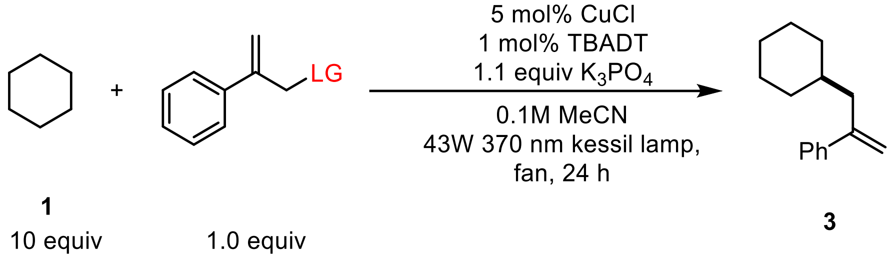

Entry[a]	Leaving Group	Conversion (%)[b]	3 (%)[b]
1	Cl	62	37
2	Br	61	19
3	OTs	40	5
4	OBoc	88	-
5	OTf	45	-
6	OP(O)(OEt)$_2$	11	-
7	OAc	13	-

Table S2. a) Conditions: 0.5 mmol of **2**, 5 mL of MeCN. b) Yield and conversion were determined by ^{1}H-NMR using 1,3,5-trimethoxy benzene as internal standard.

8.3. Evaluation of different bases

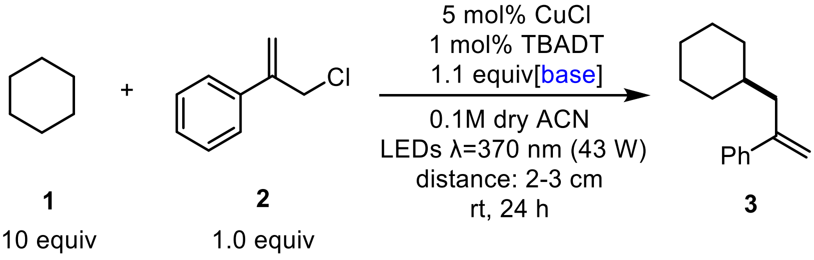

Entry[a]	Base	Conversion (%)[b,c]	3 (%)[b,c]
1	K_3PO_4	62 (>95)	37 (49)
2	KOAc	65	30
3	Na_3PO_4	62	43
4	TBA BNP	100	25
5	DIPEA	76	35
6	DBU	(80)	(10)
7	Pyridine	(66)	(30)
8	DMAP	(77)	(12)
9	Lutidine	62 (>92)	38 (30)
10	2,6-dtbpy	57	39
11	Collidine	82 (>95)	55 (43)

Table S3. a) Conditions: 0.5 mmol of **2**, 5 mLof MeCN. b) Yield and conversion were determined by ^{1}H-NMR using 1,3,5-trimethoxy benzene as internal standard. c) Values in brackets using 6 mol% of PPh_3 as ligand. DMAP = 4-(Dimethylamino)pyridine DIPEA = *N*, *N*-Diisopropylethylamine. Lutidine = 2,6-Dimethylpyridine. 2,6-dtbpy = 2,6-Di-*tert*-butylpyridine. Collidine = 2,4,6-Trimethylpyridine. TBA BNP= Tetrabutylammonium 1,1'-binaphtyl-2,2'-diyl hydrogenphosphate

8.4. Evaluation of different ligands on copper

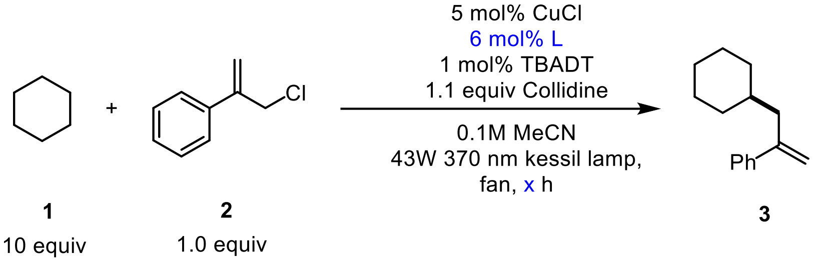

Entry[a]	Ligand	time (h)	Conversion (%)[b]	3 (%)[b]
1	-	4	80	50
		24	82	55
2	PPh_3	1	70	44
		2	85	52
		4	>95	62
		8	>95	58
		24	>95	43
3	PCy_3	4	80	50
		8	>95	55
		24	>95	55
4	$P(OPh)_3$	4	80	51
		8	>95	50
		24	>95	50
5	1,10-phen	24	50	25
6	PyrOx	24	61	26

Table S4. a) Conditions: 0.5 mmol of **2**, 5 mL of MeCN. b) Yield and conversion were determined by ^{1}H-NMR using 1,3,5-trimethoxy benzene as internal standard. PPh_3 = Triphenylphosphine. PCy_3 = Tricyclohexylphosphine. 1,10-phen =1,10-Phenanthroline. PyrOx = 2-(pyridin-2-yl)-4,5-dihydrooxazole.

8.5. Evaluation of different Cu complexes under optimized conditions

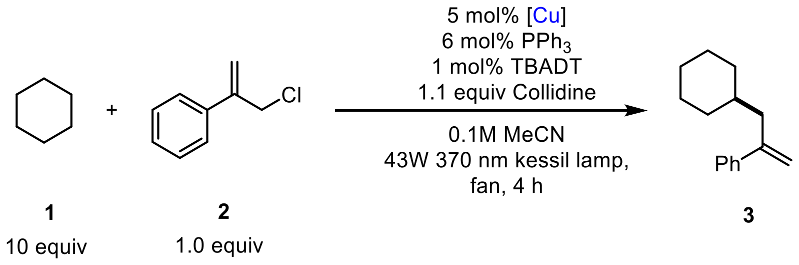

Entry[a]	Cu Catalyst	Conversion (%)[b]	Yield (%)[b]
1	CuCl	>95	62
2	CuI	91	56
3	$Cu[MeCN]_4PF_6$	91	56
4	$Cu(OTf)_2$	90	54
5	$CuCl_2$	>95	61

Table S5. a) Conditions: 0.5 mmol of **2**, 5 mL of MeCN. b) Yield and conversion were determined by ^{1}H-NMR using 1,3,5-trimethoxy benzene as internal standard. PF_6 = Hexafluorophosphate.

9. Control Experiments

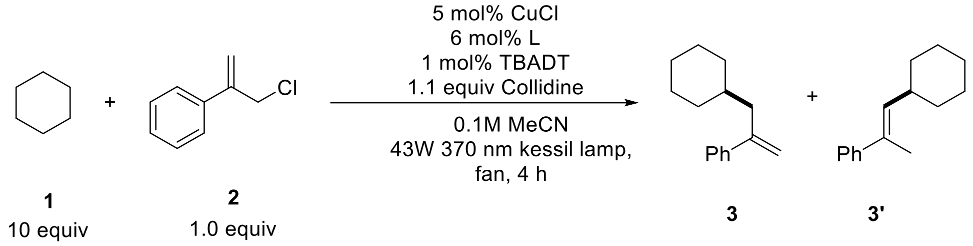

Entry[a]	Deviations	Conversion (%)[b,c]	Yield (%)[b,c]
1	-	>95 (>95)	62 (43)
2	No Base	70 (82)	43[d,e] (51)[d,f]
3	No Cu	70 (85)	29 (50)
4	No Light	-	-
5	No light, 80˚C	-	-
6	No TBADT	-	-
7	5 eq CyH	90 (92)	51 (55)
8[g]	0.05 M	86	57
9	1 h light, 3 h dark	70	44
10	1 h	70	44

Table S6. a) Conditions: 0.5 mmol of **2**, 5 mL of MeCN. b) Yield and conversion were determined by ^{1}H-NMR using 1,3,5-trimethoxy benzene as internal standard. c) Results in brackets correspond to 24 h instead of 4 h. d) Combined yield of **3** and **3'**. e) **3**:**3'** = 1.4:1. f) **3**:**3'** = 1:10). g) Conditions: 0.25 mmol of **2**, 5 mL of MeCN. TBADT = Tetrabutylammonium decatungstate. CyH = Cyclohexane.

10. Mechanistic experiments

10.1. Deuterium labelling experiments

2-*d* (3-chloroprop-1-en-2-yl-3,3-d_2)benzene was synthesized according to a literature procedure.[3] The product was obtained with a 98% of deuterium in the allylic position (Figure S2).

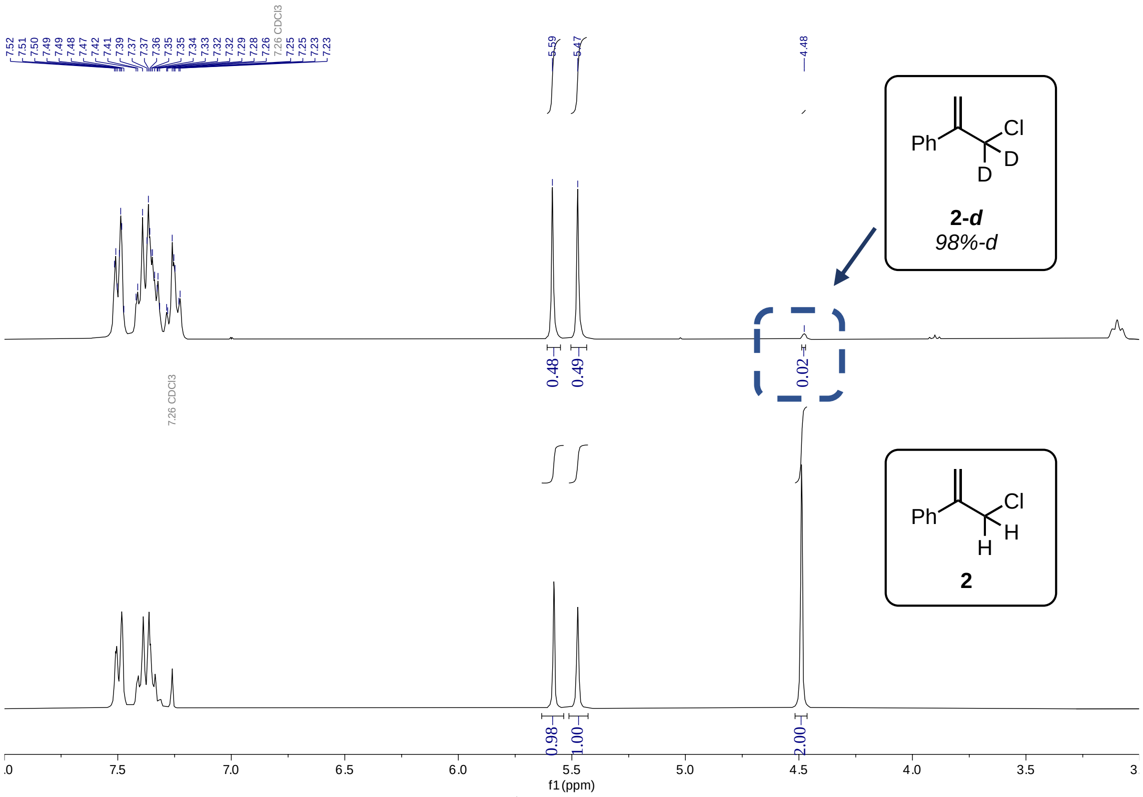

Figure S2. ^{1}H-NMR of **2** and **2-*d***

10.1.1. C-H allylation of cyclohexane using deuterated allylic chloride 2-*d*

The reaction was carried out following the general procedure for the direct allylation of alkanes (section 4).

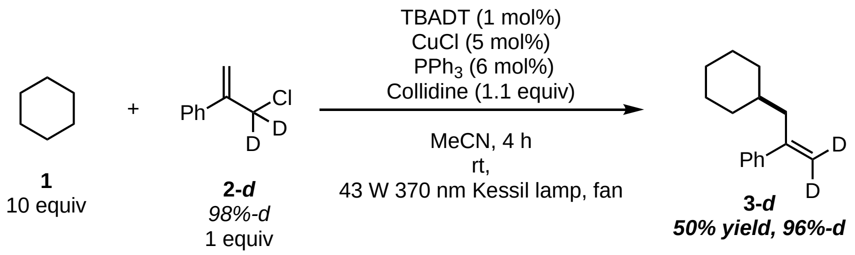

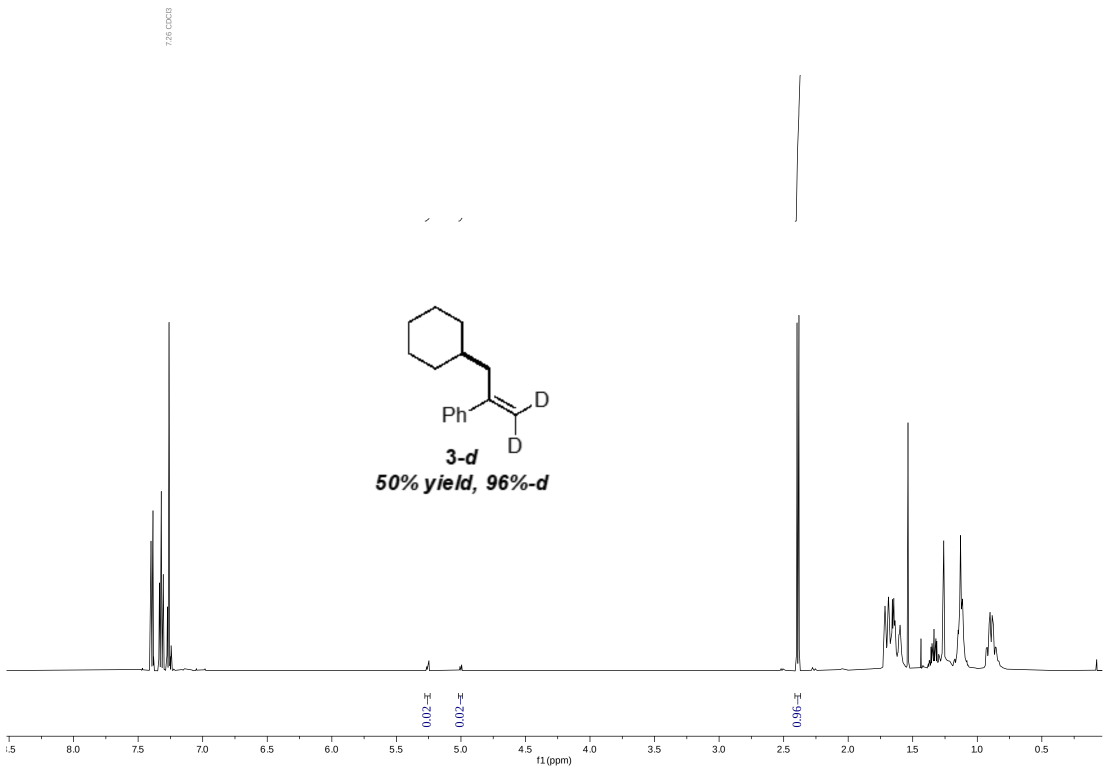

Figure S3. ^{1}H-NMR spectra of **3-*d***

10.2. Radical clock experiment

<u>Reaction in the absence of cyclohexane:</u>

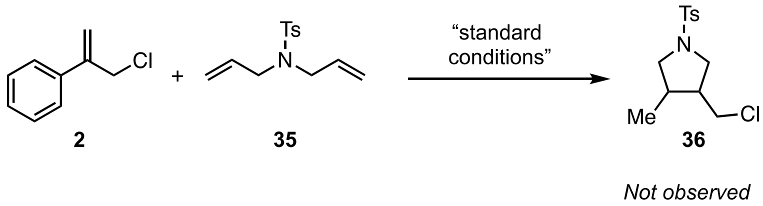

The experiment was done following the general procedure, using **35** (5 mmol) instead of cyclohexane.

<u>Observation:</u> We did not observe **36** neither by ^{1}H-NMR or by GC-MS analysis. This suggests that free chlorine radical is not generated by homolytic C-Cl cleavage under the standard conditions. Therefore, it seems unlikely that Cl· acts as HAT species in this transformation[17]:

[17] a) Treacy, S. M.; Rovis, T. *J. Am. Chem. Soc*. **2021**,*143*, 2729-2735. b) Shields, B. J.; Doyle, A. G. *J. Am. Chem. Soc*. **2016** *138*, 12719-12722. c) Yang, Q.; Wang, Y.; Qiao, Y.; Gau, M.; Carroll, P.; Walsh, P.; Schelter, E. *Science* **2021**, *372*, 847-852. d) Yuan, R.; Fan, S.; Zhou, H.; Ding, Z.; Lin, S.; Li, Z.; Zhang, Z.; Xu, C.; Wu, L.; Wang, X.; Fu, X. *Angew. Chem. Int. Ed*. **2012**, *125*, 1069-1073. e) Nielsen, M. K. ; Shields, B. J.; Liu, J.; Williams, M. J.; Zacuto, M. J. ; Doyle, A. G. *Angew. Chem. Int. Ed*. **2017**, *56*, 7191-7194.

Reaction in the presence of cyclohexane:

The experiment was done following the general procedure, adding **35** (5 mmol) to the mixture.

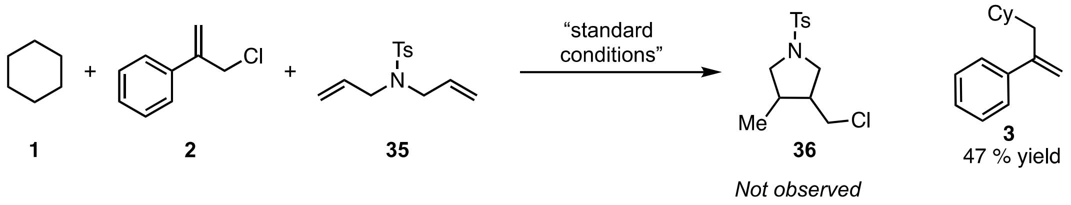

Observation: We did not observe **36** neither by ^{1}H-NMR or GC-MS analysis. Product **3** was obtained in 47% yield. This suggests that free chlorine radical is not generated after C-C bond formation either. This experiment further supports that Cl· does not act as HAT initiator in this transformation, and that the S_H2' reaction generates an oxidized $LCuCl_2$ complex.

Results in Table S6 (section 8), entries 9 and 10 further support that Cl· does not act as HAT initiator since conversion does not increase once the light is turned off.

10.3. Radical trapping experiment

The radical trapping experiment was performed using TEMPO as radical scavenger. The experiment was performed following the general procedure, adding 2 equiv of TEMPO.

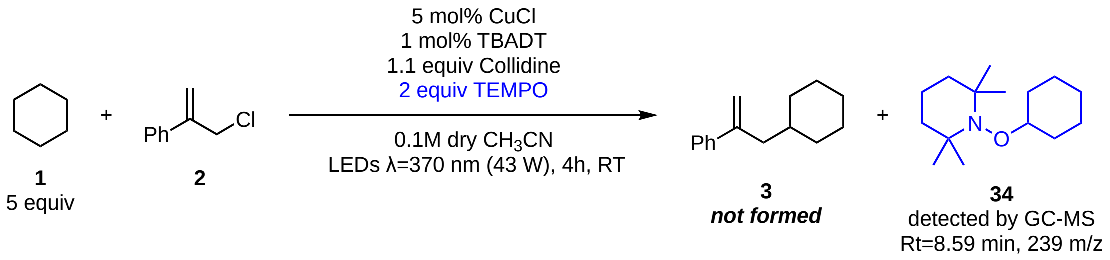

Observation: ^{1}H-NMR and GC-MS analysis of the reaction crude revealed the formation of the TEMPO-cyclohexyl adduct **34** was obtained (rt: 8.59 min, 239 m/z). Conversely, product **3** was not formed. The formation of allyl-TEMPO adduct was neither observed, thus suggesting that an allylic radical which might be generated by homolytic cleavage of the C−Cl bond is not formed under the reaction conditions.

10.4. UV-Vis experiments

UV-Vis experiments were conducted to gain further information about the mechanism of the reaction. Indeed, Cu(II) species shows a characteristic band in the UV-vis around λ=465 nm.[18] Thus, we have carried out a variety of experiments to evaluate the potential formation of different Cu(II)-species under reaction conditions.

[18] (a) Ishiguro, S.-I.; Jeliazkova, B. G.; Ohtaki, H. *Bull. Chem, Soc. Jpn.* **1985**, *85*, 1749-1754. (b) Mereshchenko, A. S.; Olshin, P. K.; Karimov, A. M.; Skripkin, M. Y.; Burkov, K. A.; Tveryanovich, Y. S.; Tarnovsky, A. N. *Chem. Phys. Lett.* **2014**, *615*, 105-110. (c) Treacy, S. M.; Rovis, T. *J. Am. Chem. Soc.* **2021**, *143*, 2729-2735.

10.4.1. Reaction blanks

A variety of studies were carried out to discard any band of other substrate that can disturb the characteristic band of Cu(II) in our studies.

10.4.1.1. TBADT

UV-Vis spectra of TBADT was obtained at different concentrations. 5mM (blue), 2.5 mM (orange), 1.25 mM (green) and 0.625 mM (yellow). It was observed in our spectra window a strong absorption band around 323 nm according to previously reported.[19]

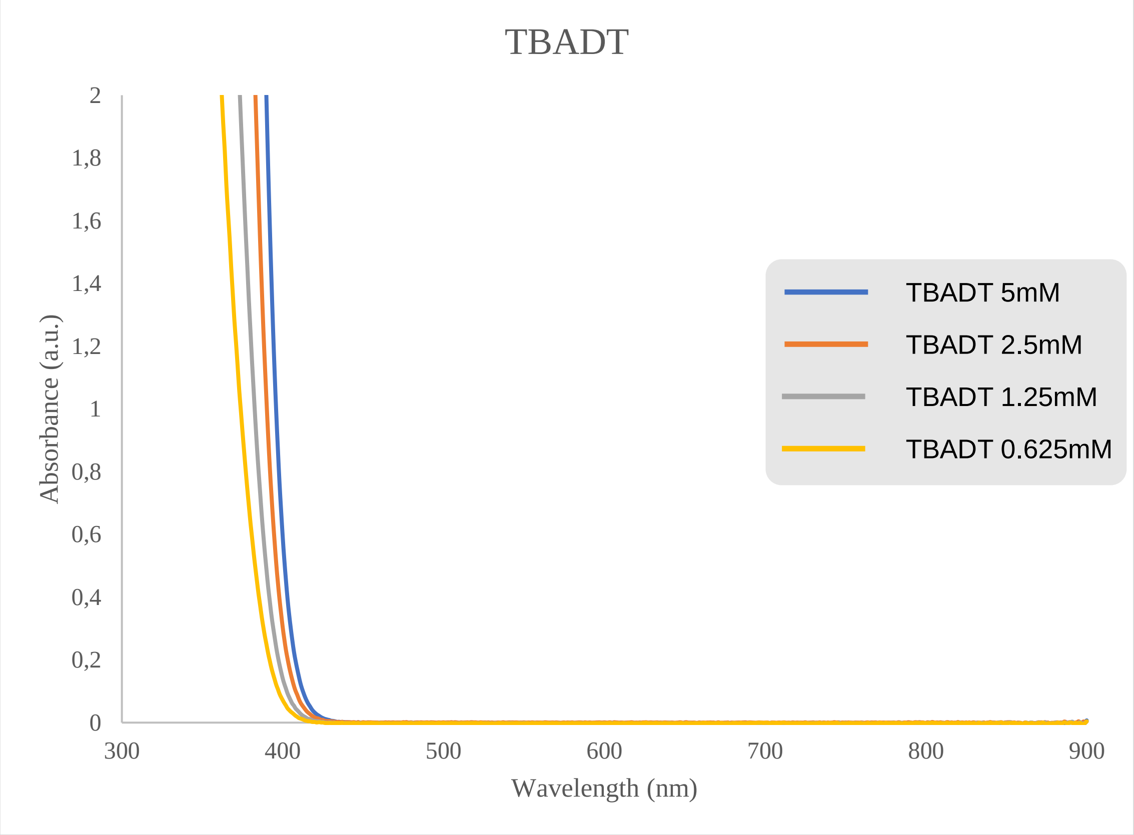

Figure S4. UV-vis plot of the TBADT at different concentrations

10.4.1.2. No UV-Vis active species

Cyclohexane, allylic chloride **2**, collidine and the product **3** were found to be no UV active at 5 mM between 350 and 900 nm.

[19] Yamase, T., Takabayashi, N.; Kaji, M. *J. Chem. Soc., Dalton Trans*. **1984**, 793-799.

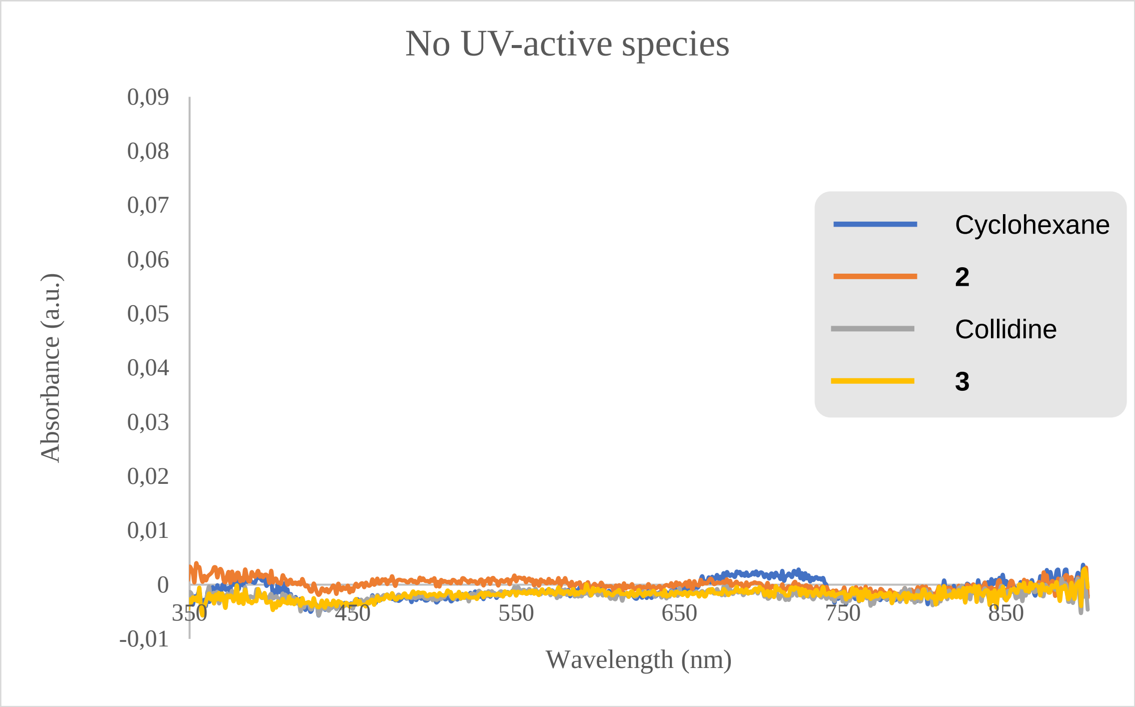

Figure S5. UV-vis plot of the non-UV active species

10.4.2. Cu (I) species

CuCl and the complex CuCl·**2** were not active between 300 and 600 nm.

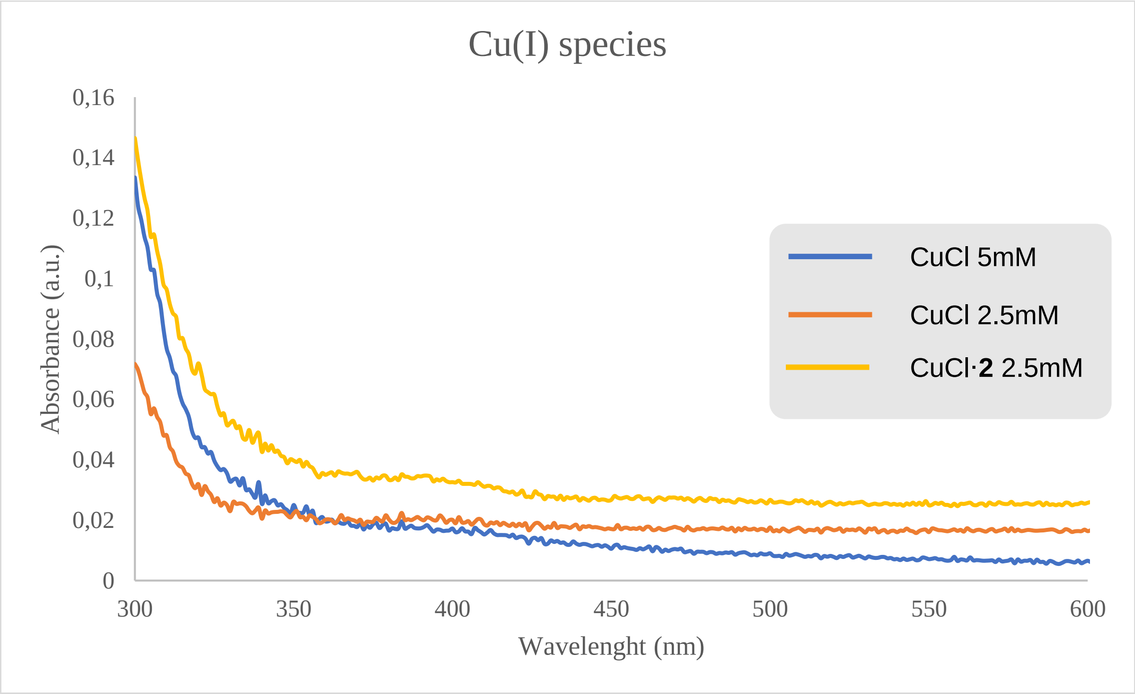

Figure S6. UV-vis plot of the Cu(I) species at different concentrations

10.4.3. Cu(II) species

$CuCl_2$ and the complex $CuCl_2$·**2** showed a characteristic band at 465nm at different concentrations.

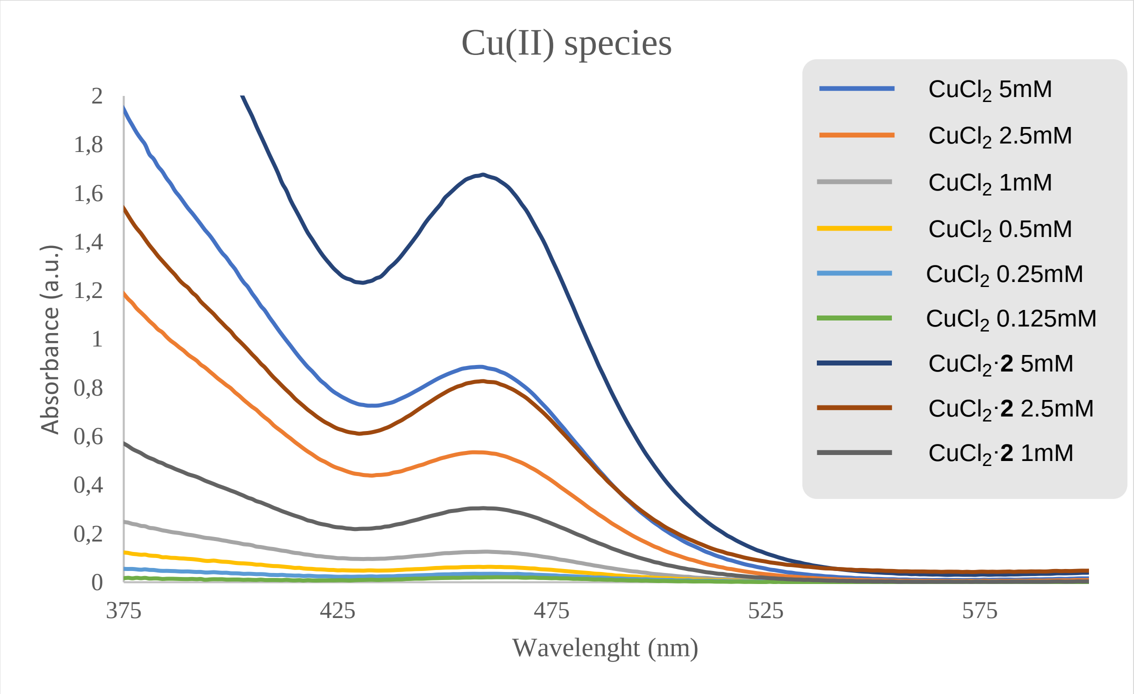

Figure S7. UV-vis plot of Cu(II) species at different concentrations

10.4.4. Evaluation of the potential formation of alkyl-Cu(II) species

The following experiment was carried out to assess the possibility of the formation of an alkyl-Cu(II) species ed by combination of the cyclohexyl radical with CuCl.

In an oven-dried 8 mL vial A solution of cyclohexane **1** (10 equiv), CuCl (3 mol%) and TBADT (3 mol%) in 3 mL of MeCN was prepared. The solution was cooled down to 0 ºC and bubbled with argon for 5 min. Then the cap of the vial was sealed with parafilm, and the vial was placed to the direct flux of LED on the vial holder at 2 – 3 cm from the light source for 3 h. In order to keep the temperature below 30 ºC, a fan was placed in front of the vials (T Max.= 30 ˚C) (See figure S1). Afterwards, the vial was open to air and UV-vis experiments were recorded at different concentrations 3.3mM (blue), 1.65mM (orange), 0.82 mM (grey) and 0.41mM (yellow).

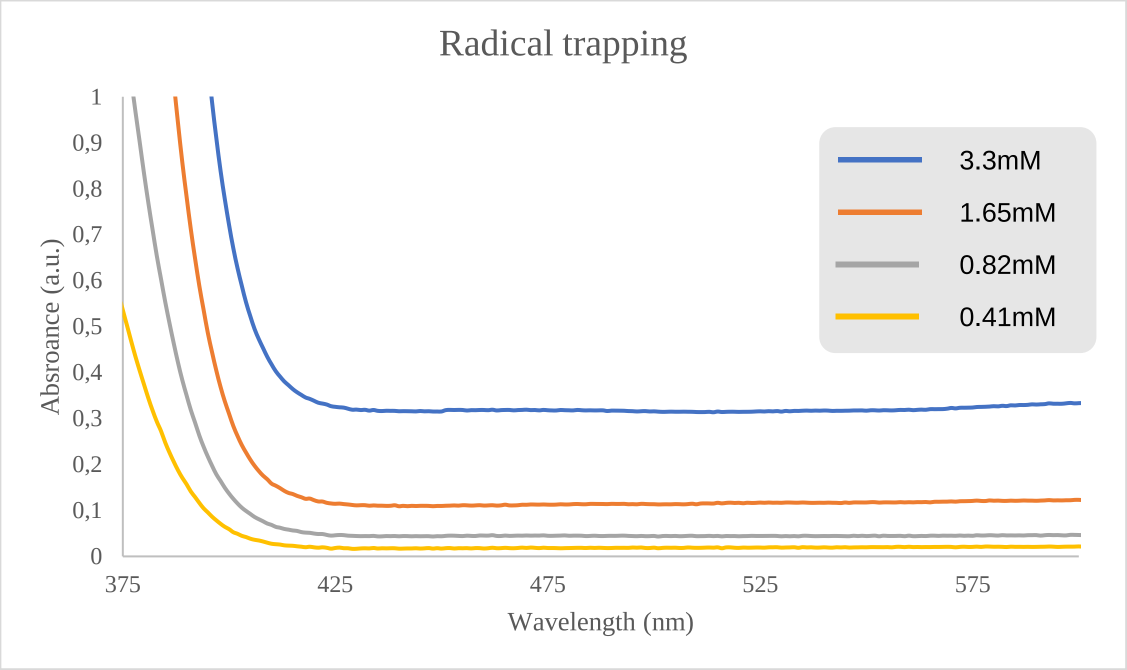

Figure S8. UV-vis plot of the radical trapping experiment at different concentrations

Observation: The characteristic band of Cu(II) λ=465nm was not observed. This suggests that alkyl-Cu(II) species are not formed by combination of the cyclohexyl radical with CuCl.

10.4.5. Evaluation of the potential formation of Cu(II) after the C-C bond forming event

The experiment was performed following the general procedure, albeit in the absence of collidine and PPh_3 to simplify the UV-Vis spectra, by avoiding the characteristic charge transfer bands associated to the possible phosphine and/or collidine-Cu complexes.[20]
An oven-dried 8 mL vial equipped with stirring magnetic bar was charged with the TBADT (1 mol%), **2** (0.5 mmol), cyclohexane (5 mmol), CuCl (5 mol%) and 5 mL of dry MeCN, and the vial was wrapped with aluminum foil. The vial was cooled-down to 0ºC and bubbled with argon for 10 min.Then the cap of the vial was sealed with parafilm, and the vial was placed to the direct flux of LED on the vial holder at 2 – 3 cm from the light source. In order to keep the temperature below 30 ºC, a fan was placed in front of the vials (T Max.= 30 ˚C) (See figure X). After 3 h (no full conversion of the allyl chloride), the vial was open to air and UV-Vis experiments were recorded at different concentrations 5mM (light blue), 2.5mM (yellow) and 1.25mM (grey).

[20] Małecki, J. G.; Maroń, A.; Palion, J.; Nycz, J. E.; Szala, M. *Trans. Met. Chem.* **2014**, *39*, 755-762.

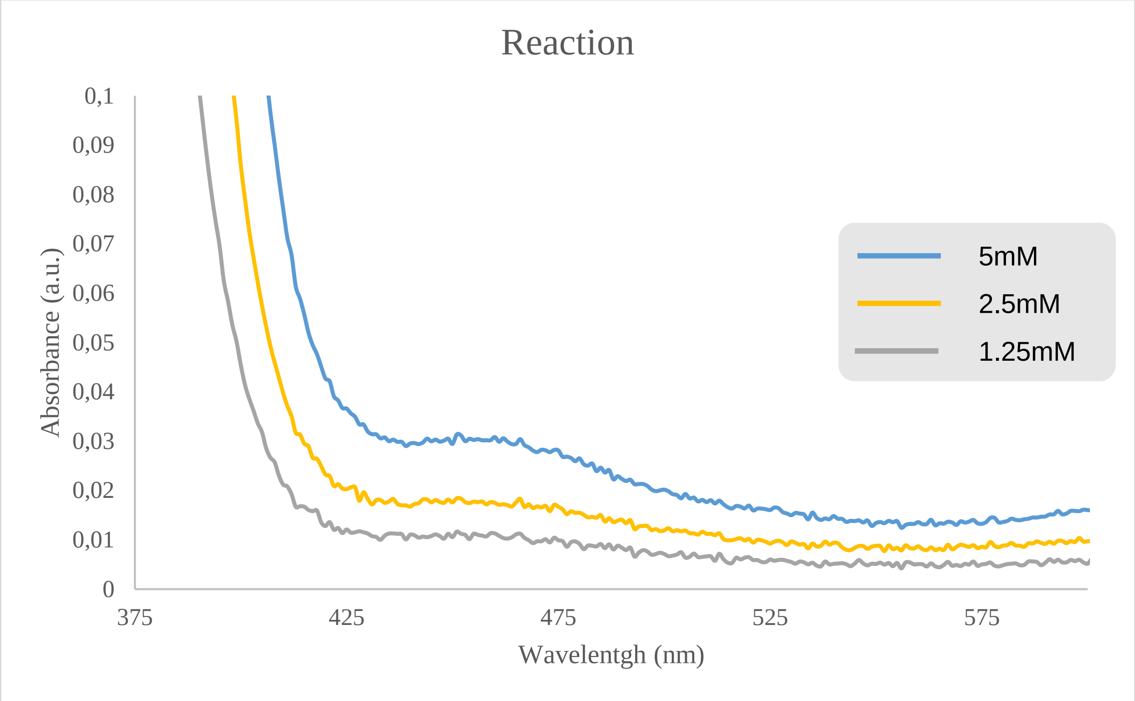

Figure S9. UV-vis plot of the reaction at different concentrations

Observation: The characteristic band of $CuCl_2$ λ=465nm was observed during the experiment. This shows that $CuCl_2$ is formed when both the alkane and the allyl chloride are present in the reaction media, and suggests that the Cu(II) intermediate is formed as a result of the S_H2' reaction.

10.5. Evaluation of allyl-Cu(I) complexes formation by NMR experiments

NOTE: ^1H NMR and ^{13}C NMR spectra were recorded in a Varian 300 MHz spectrometer. Chemical shifts and integrals of ^1H NMR signals are reported in ppm relative to CD_3CN.

A flame-dried NMR tube was charged with CuCl (20 mg, 0.2 mmol) and PPh_3 (52.6 mg, 0.2 mmol) in the glove box. The NMR tube was taken out from the glove box, and a solution of the allylic chloride **2** (31 mg, 0.2 mmol) in CD_3CN (0.4 mL) under argon was added to the NMR tube and it was sonicated for 30 min at 40 ºC.

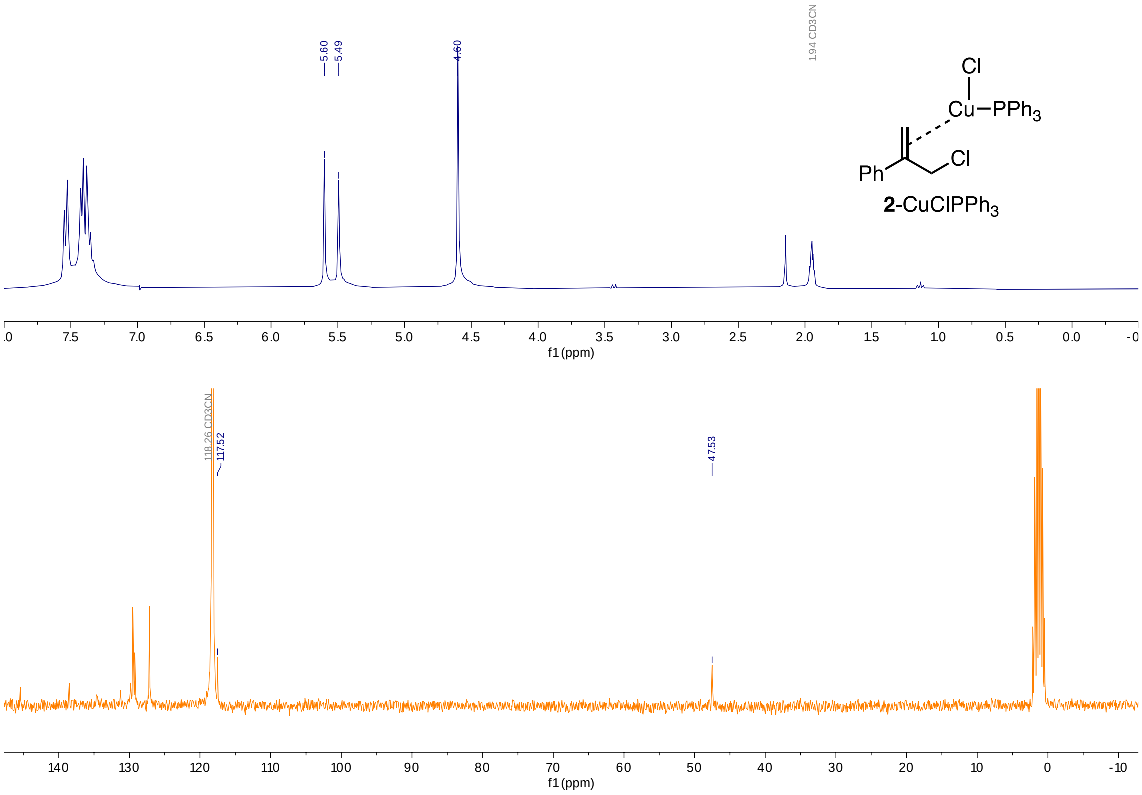

Figure S10. ^{1}H (up, blue) and ^{13}C (down, orange) NMR spectra of **2**·$CuClPPh_3$ complex

<u>Comparison between 2 and **2**·$CuClPPh_3$ spectra</u>

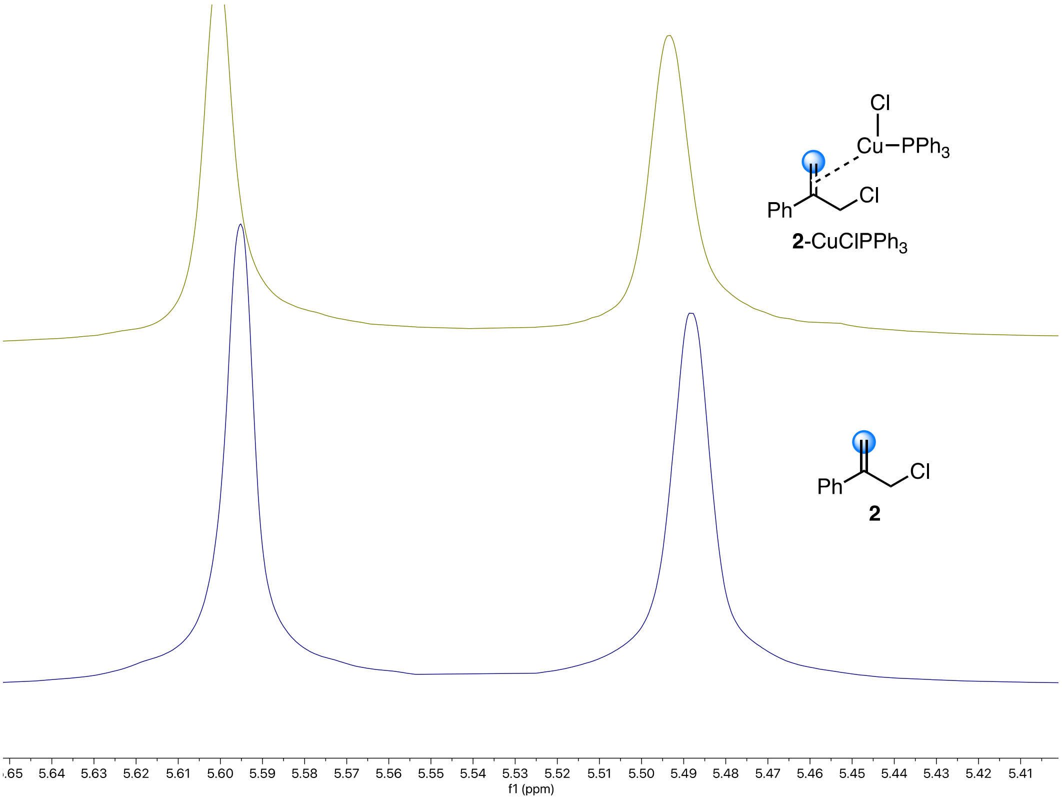

Figure S11. ^{1}H NMR spectra of the olefinic protons (highlighted in blue) **2**·$CuClPPh_3$ complex (light brown) and **2** (blue)

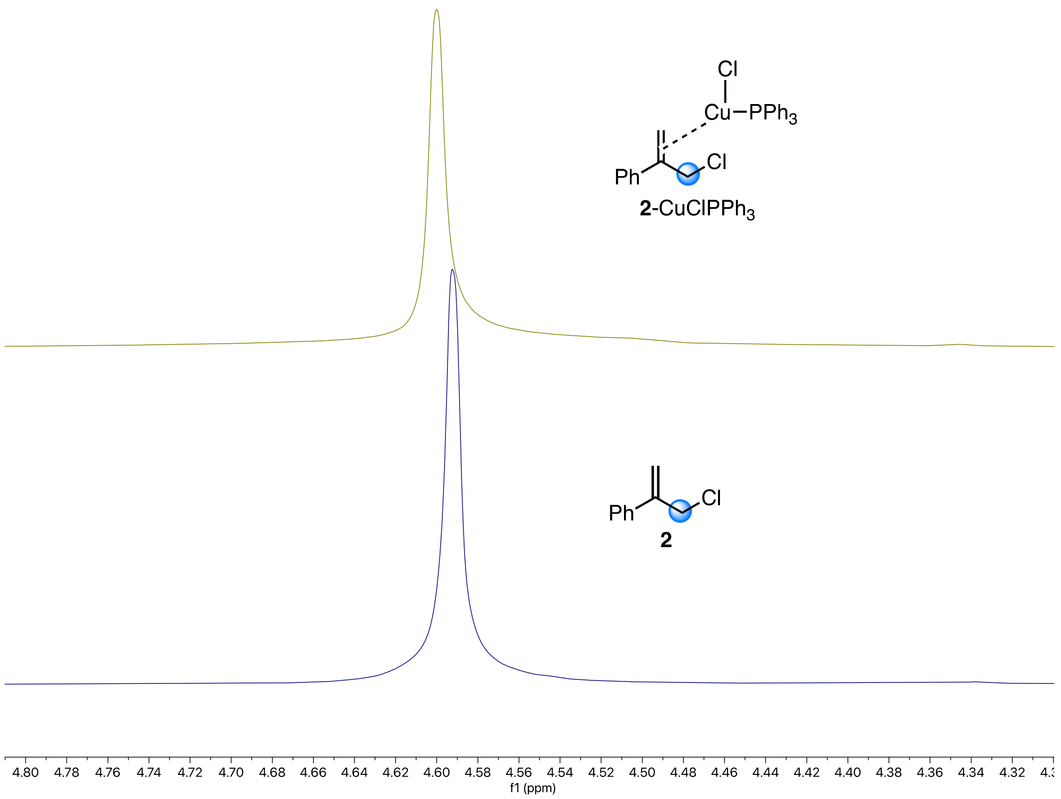

Figure S12. ^{1}H NMR spectra of the allylic protons (highlighted in blue) **2**·CuCl **2**·$CuClPPh_3$ complex (*light brown)* and **2** (*blue)*

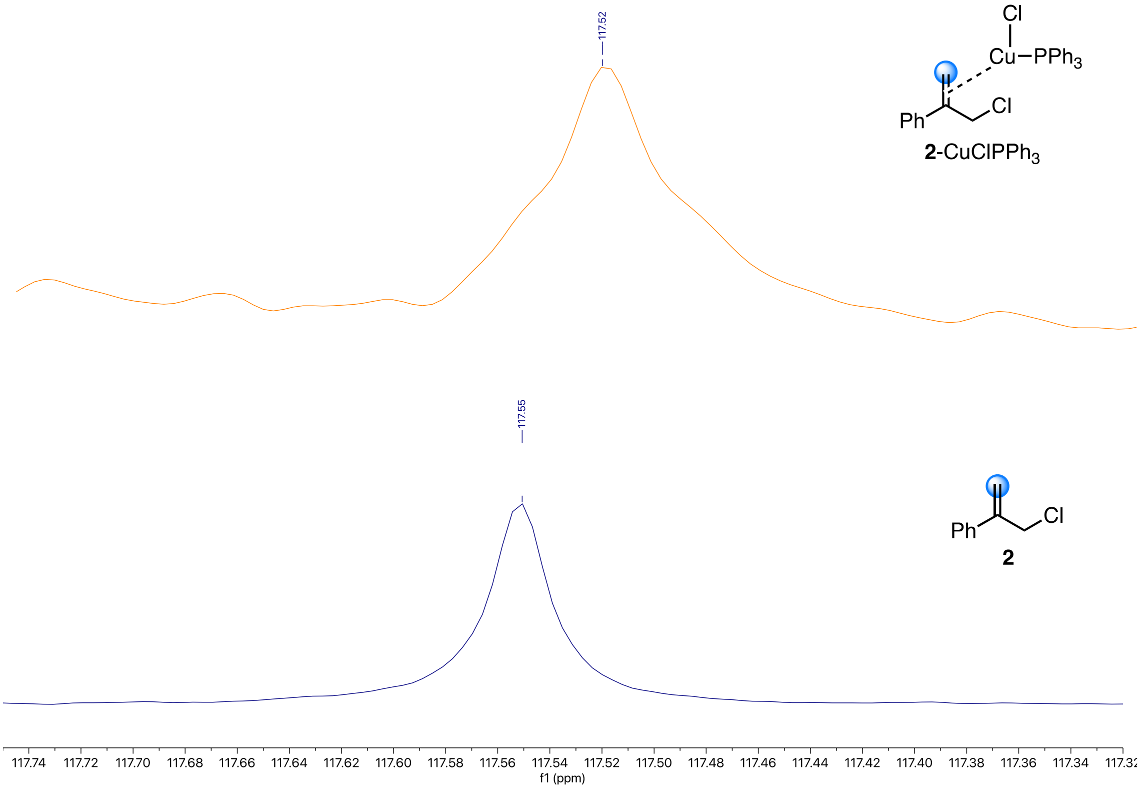

Figure S13. ^{13}C NMR spectra of the olefinic carbon (highlighted in blue) **2**·$CuClPPh_3$ complex (orange) and **2** (blue)

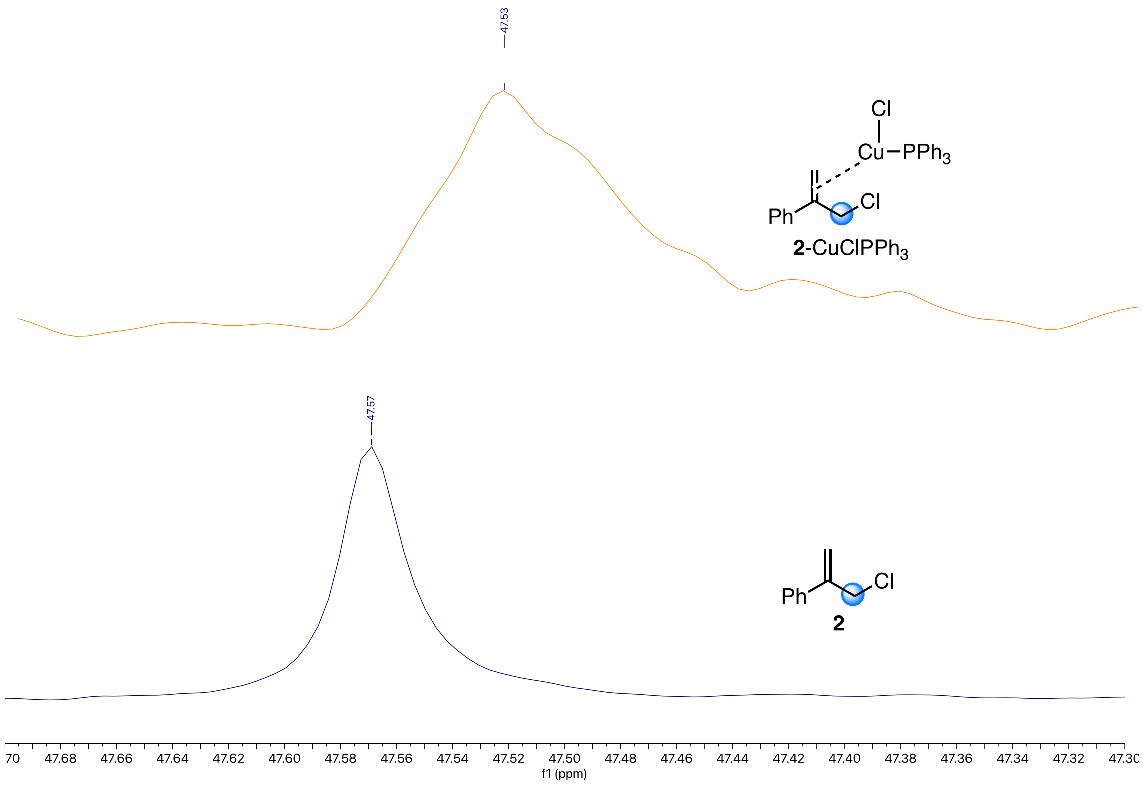

Figure S14. ^{13}C NMR spectra of the allylic carbon (highlighted in blue) **2**·CuClPPh$_3$ complex (orange) and **2** (blue)

Observation: A new set of signals was observed either by ^{1}H and ^{13}C NMR spectra when CuClPPh$_3$ was mixed with allylic chloride **2**. This shows that the allylic chloride is coordinated to the copper complex, and therefore activated.

10.6. Isomerization experiments

10.6.1. Reaction without base in the presence of product 3

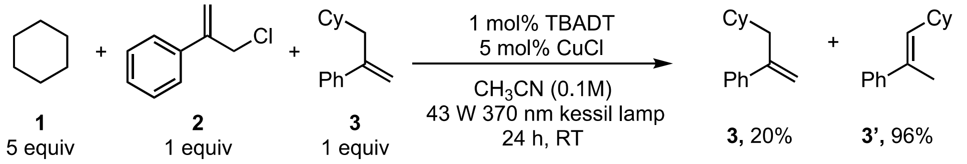

When the reaction was performed without base, regioisomer **3’** was observed in 96% yield. This result could be explained due to the presence of HCl that is generated during the reaction and promotes the isomerization of **3** to **3’**.

10.6.2. Isomerization of 3 to 3’ in acid media

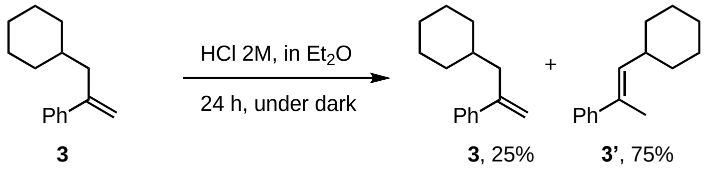

3 was stirred in the presence of HCl (2M, in Et$_2$O) under dark. A 1:3 mixture of **3** and **3’** was obtained. This result supports that product isomerization is due to the presence of free HCl.

11. NMR spectra

^{1}H NMR (500 MHz, $CDCl_3$)

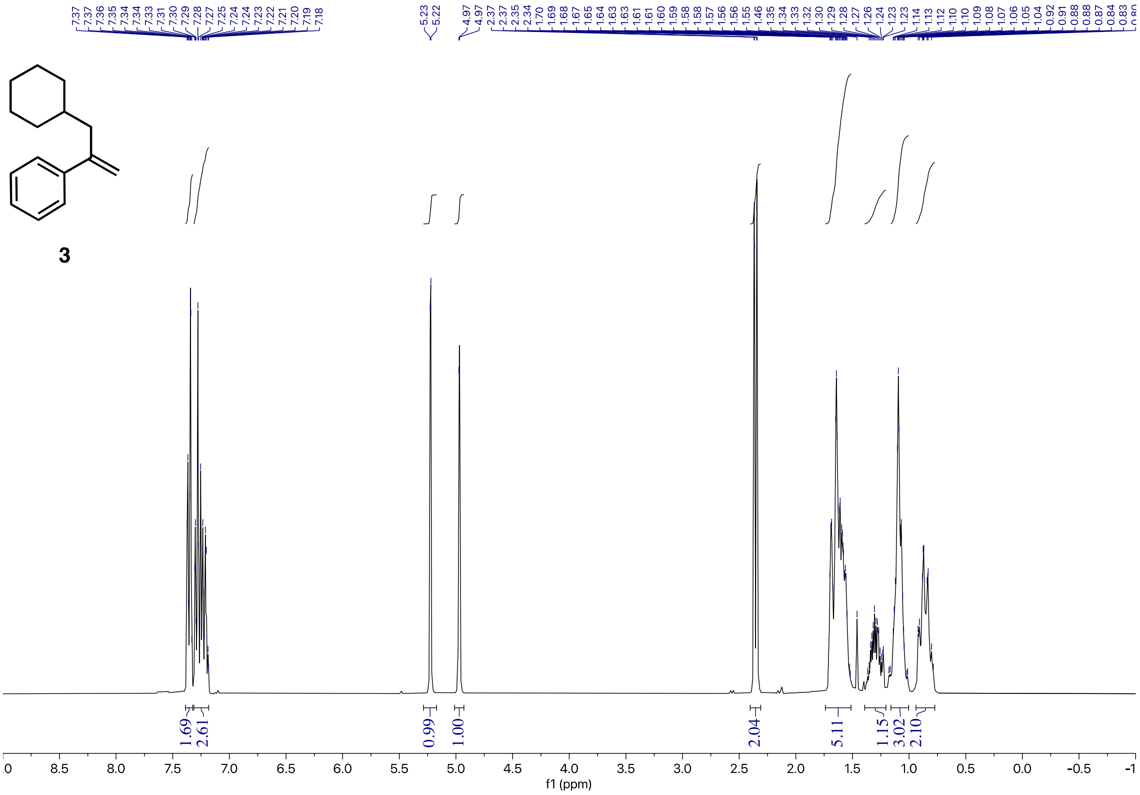

^{13}C NMR (126 MHz, $CDCl_3$)

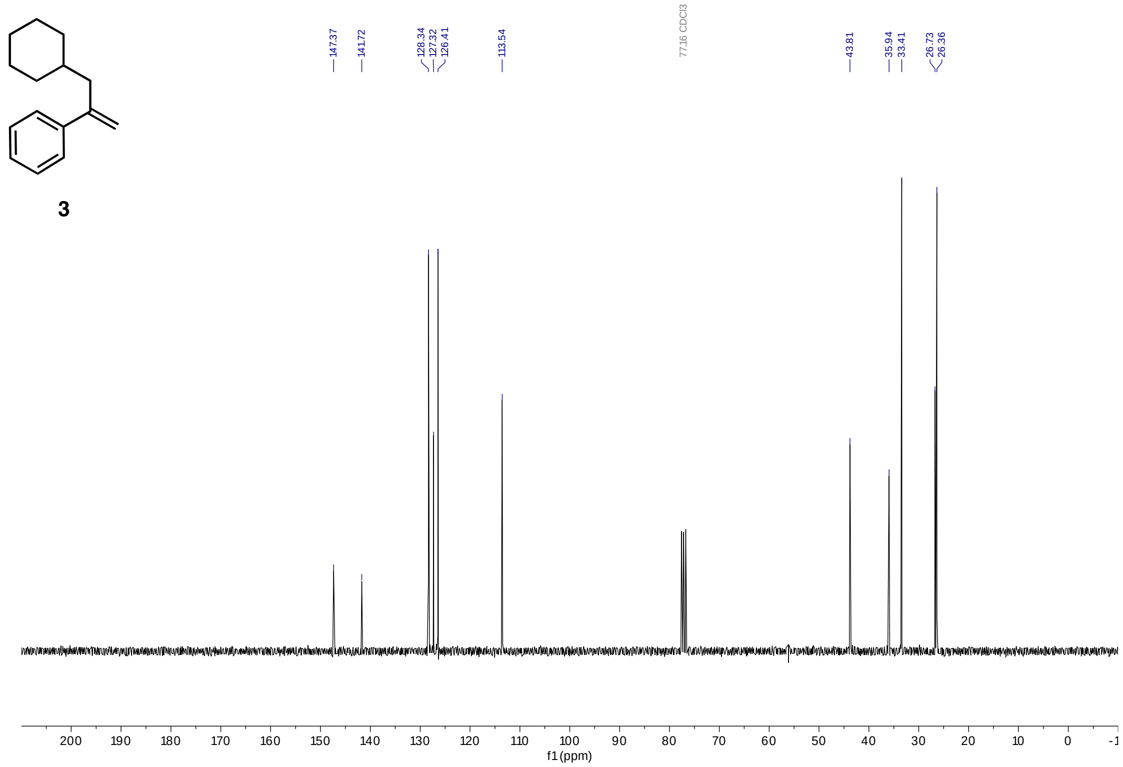

^{1}H NMR (500 MHz, $CDCl_3$)

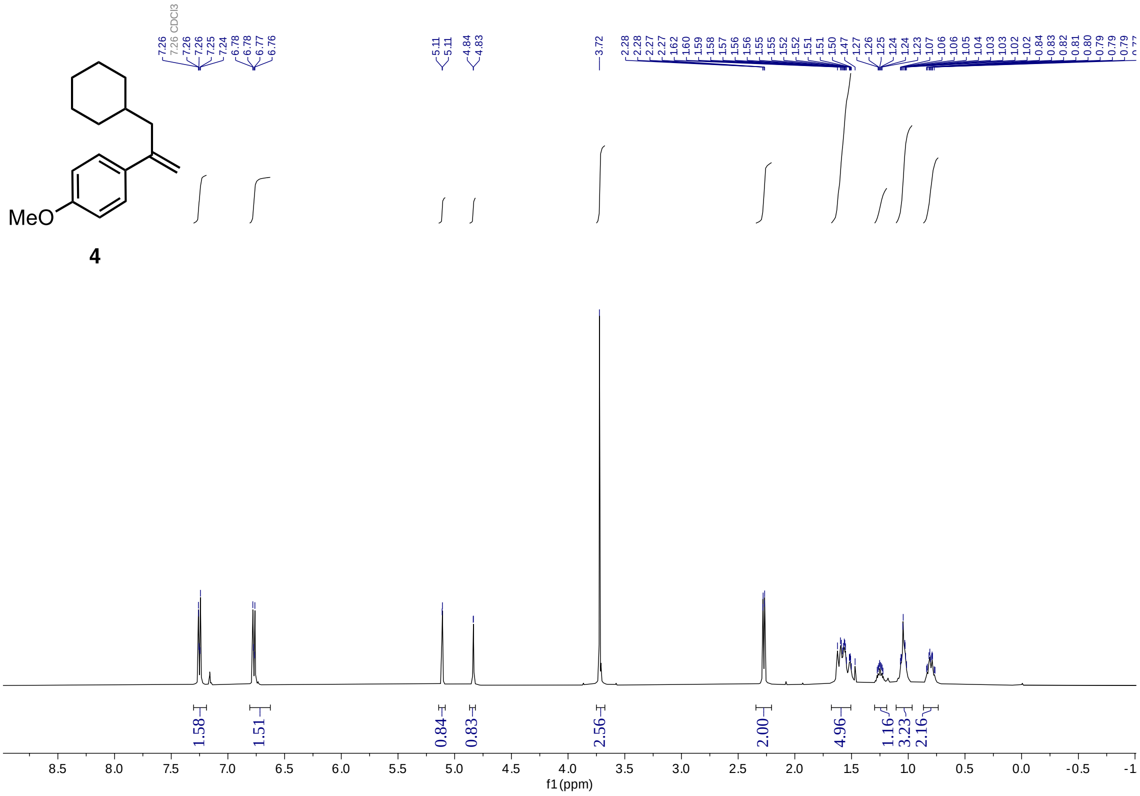

4

^{13}C NMR (126 MHz, $CDCl_3$)

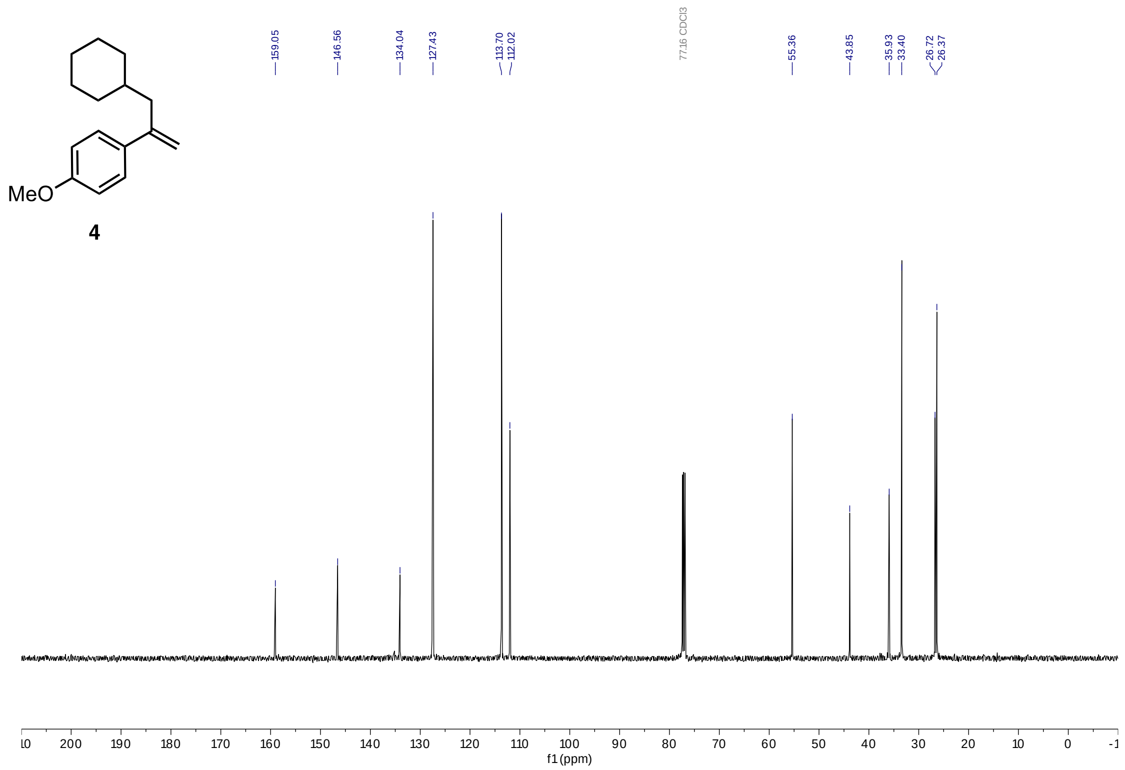

4

^{1}H NMR (500 MHz, $CDCl_3$)

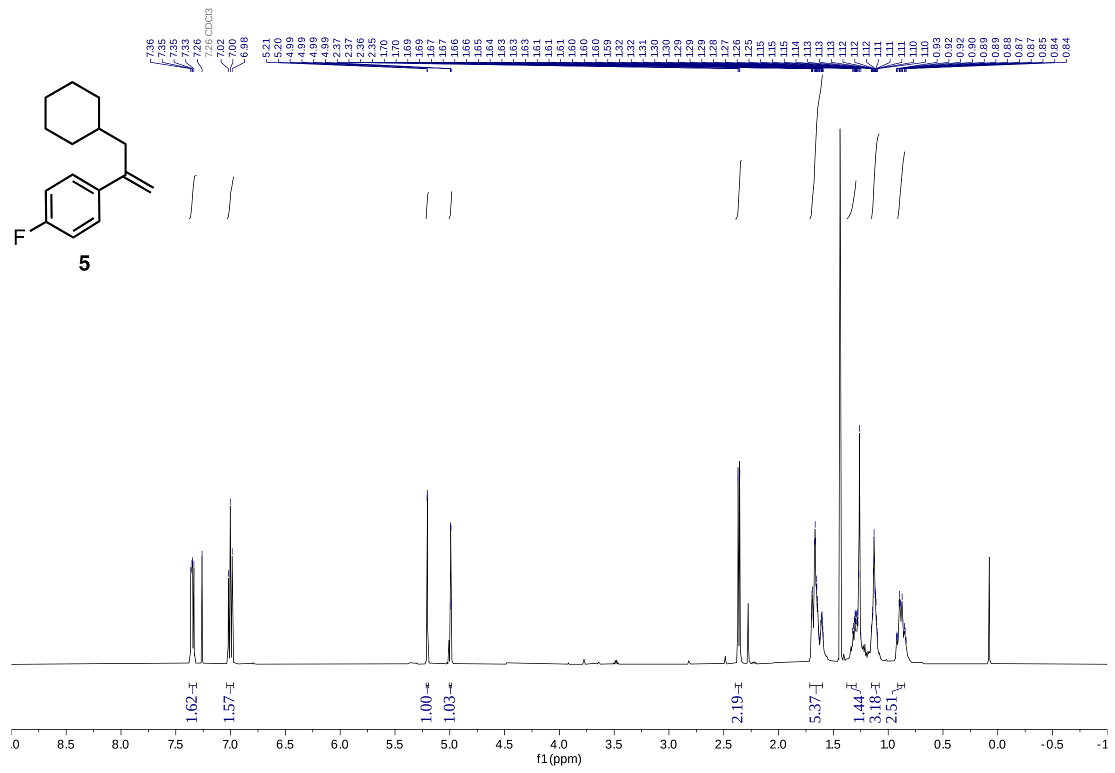

^{13}C NMR (126 MHz, $CDCl_3$)

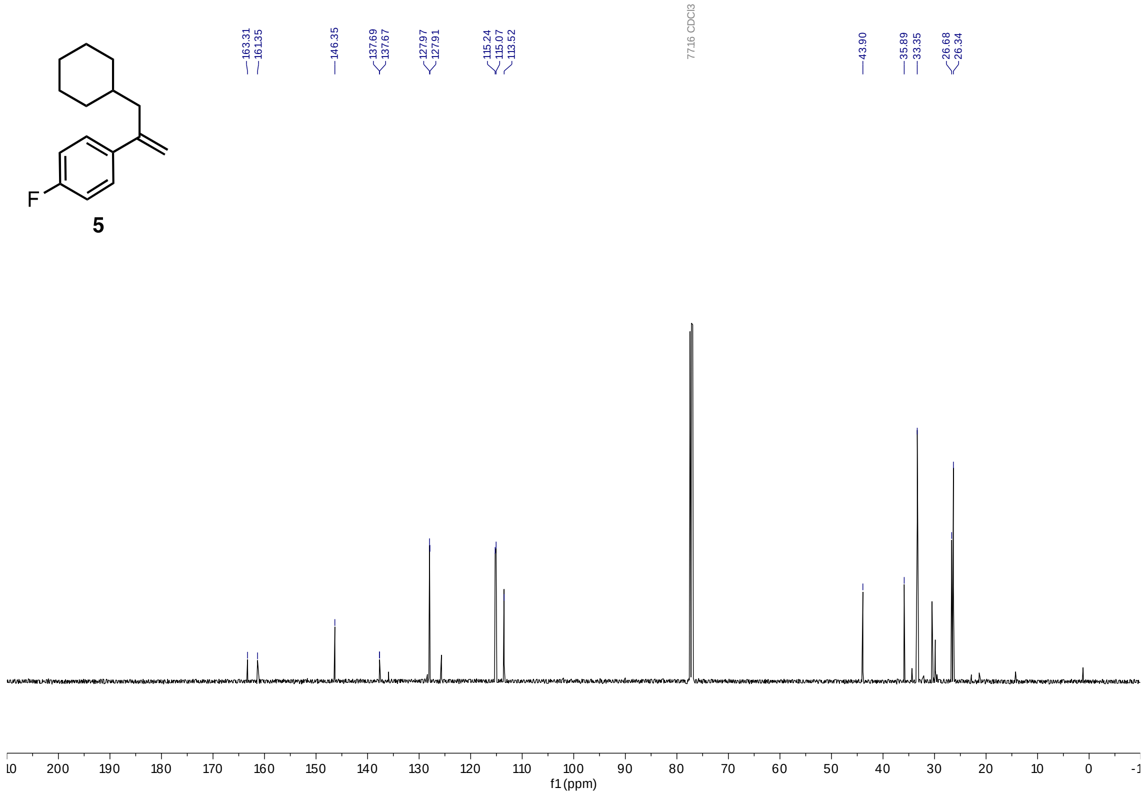

^{19}F NMR (471 MHz,$CDCl_3$)

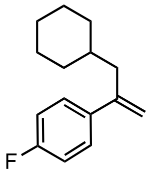

5

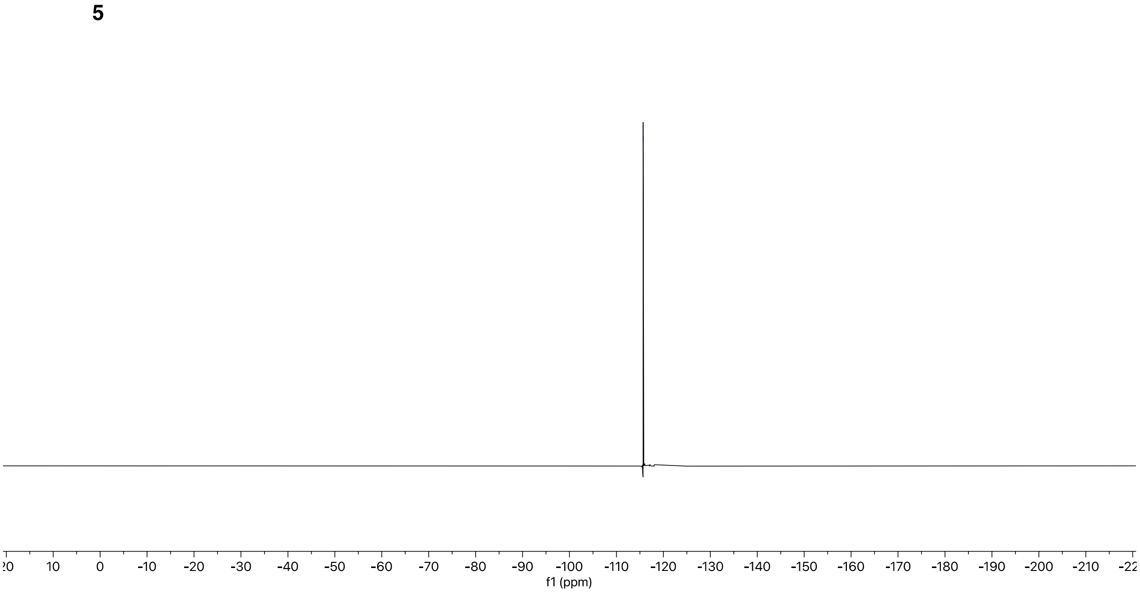

^{1}H NMR (500 MHz, $CDCl_3$)

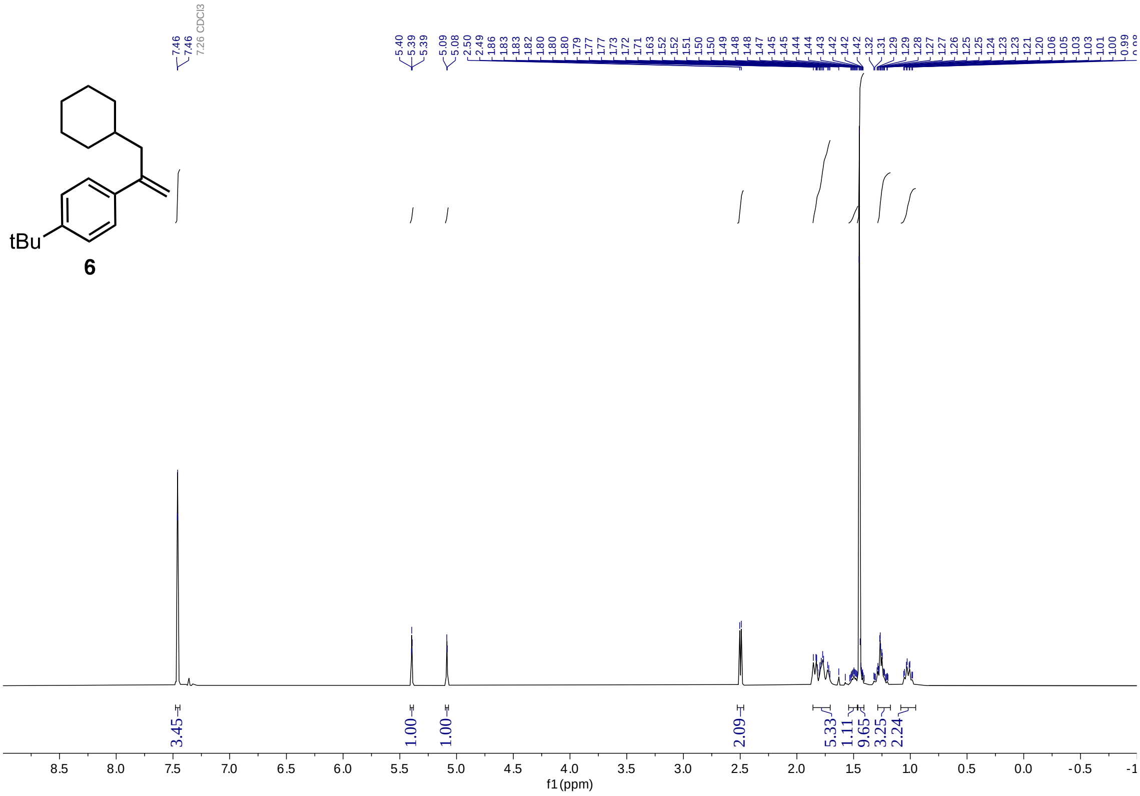

^{13}C NMR (126 MHz, $CDCl_3$)

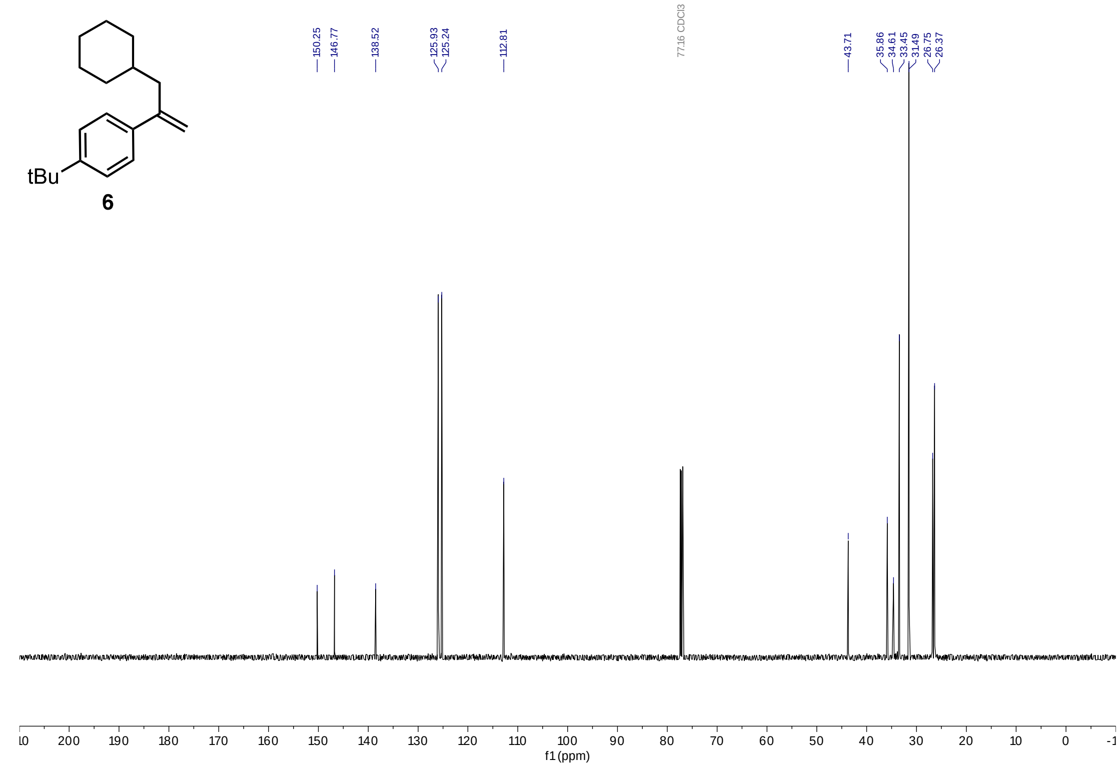

^{1}H NMR (500 MHz, $CDCl_3$)

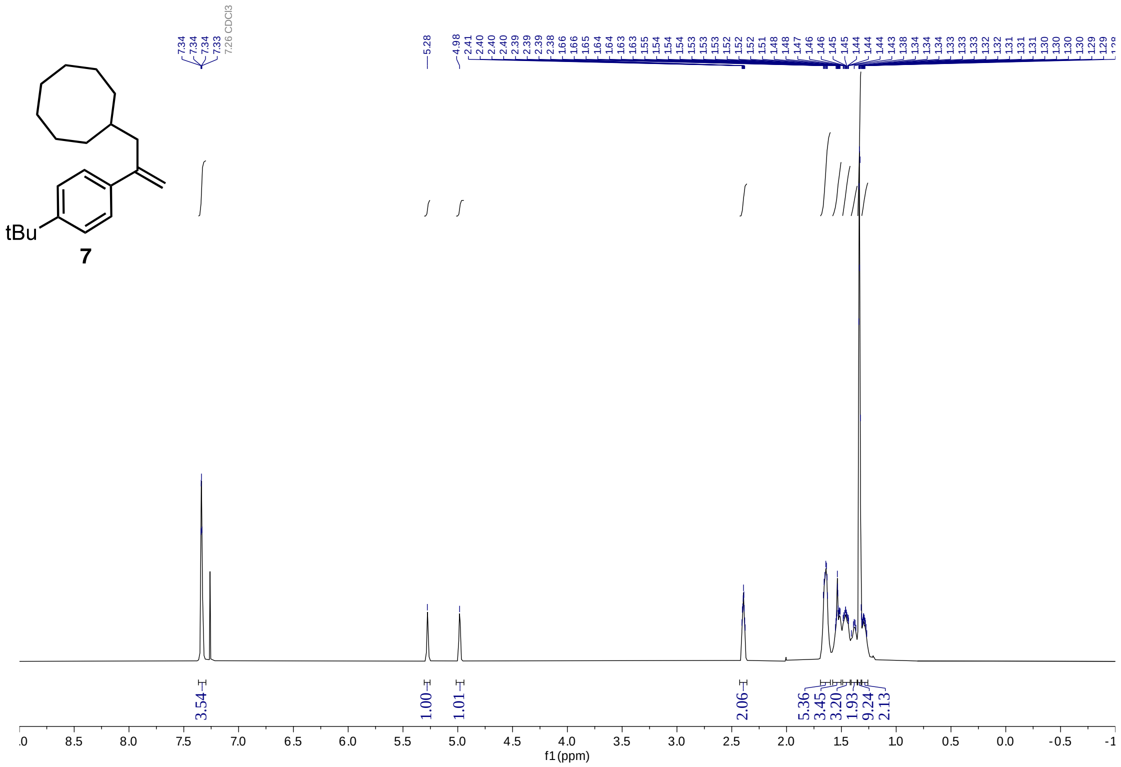

^{13}C NMR (126 MHz, $CDCl_3$)

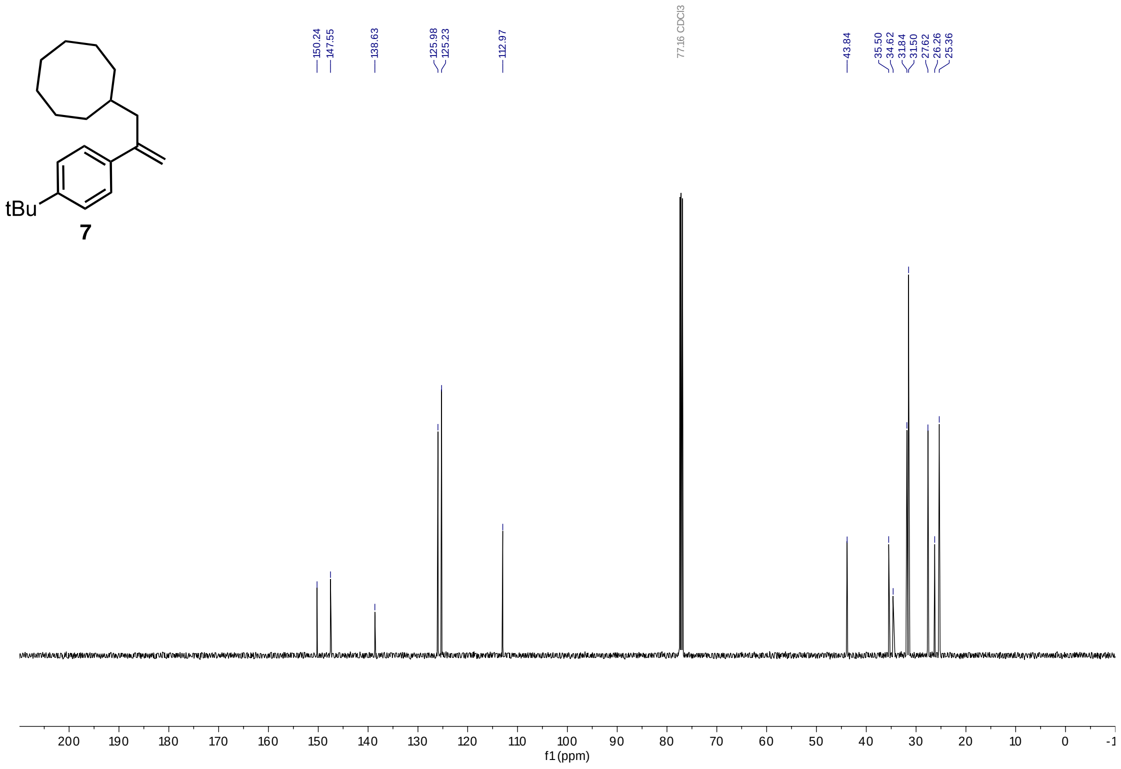

^{1}H NMR (500 MHz, $CDCl_3$)

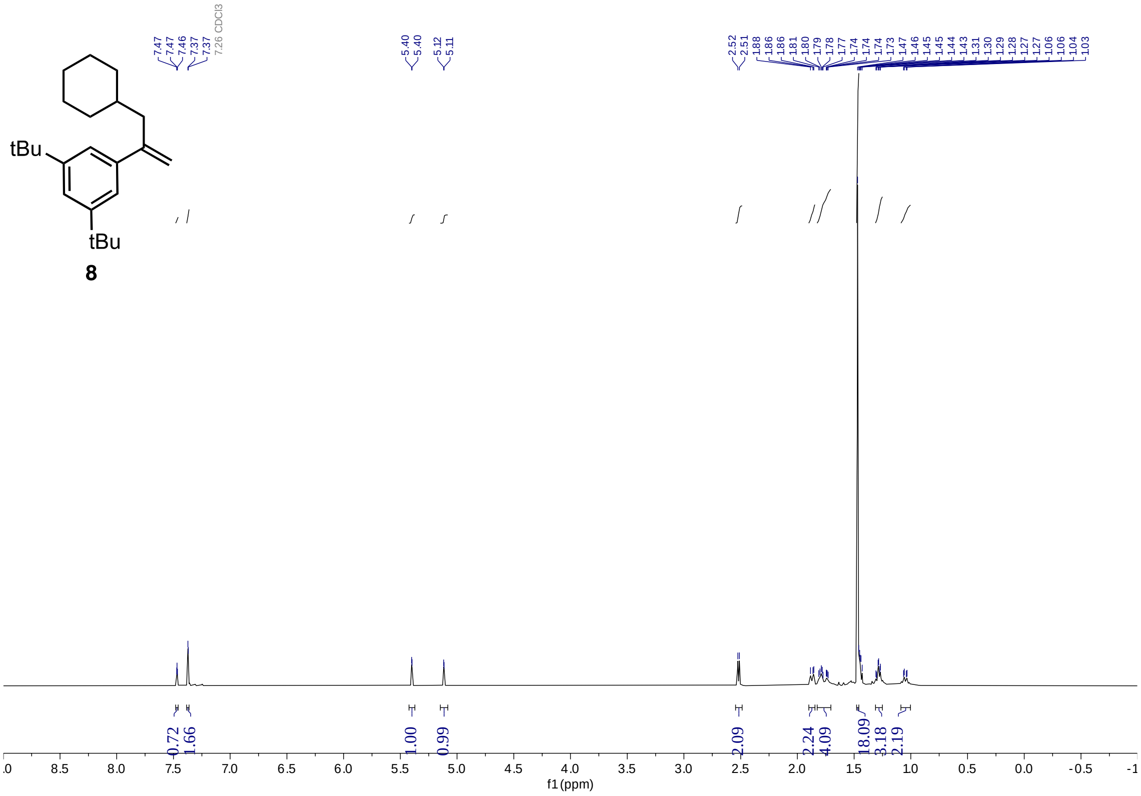

^{13}C NMR (126 MHz, $CDCl_3$)

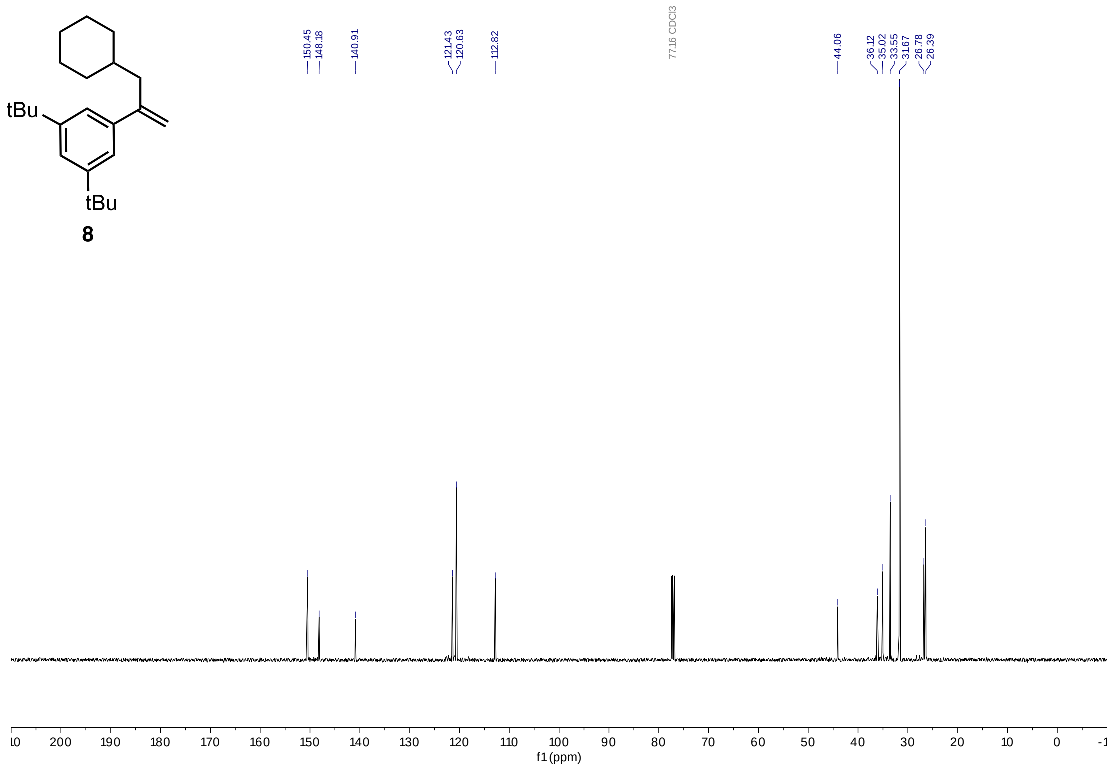

^{1}H NMR (500 MHz, $CDCl_3$)

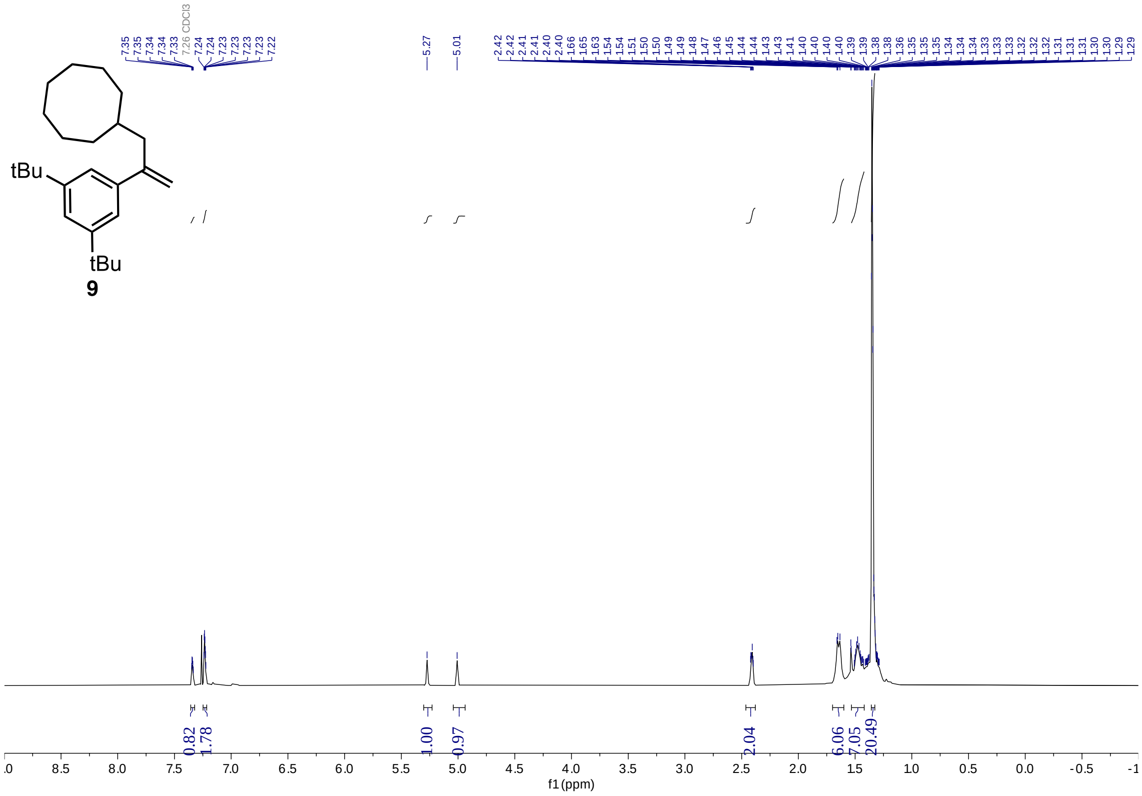

^{13}C NMR (126 MHz, $CDCl_3$)

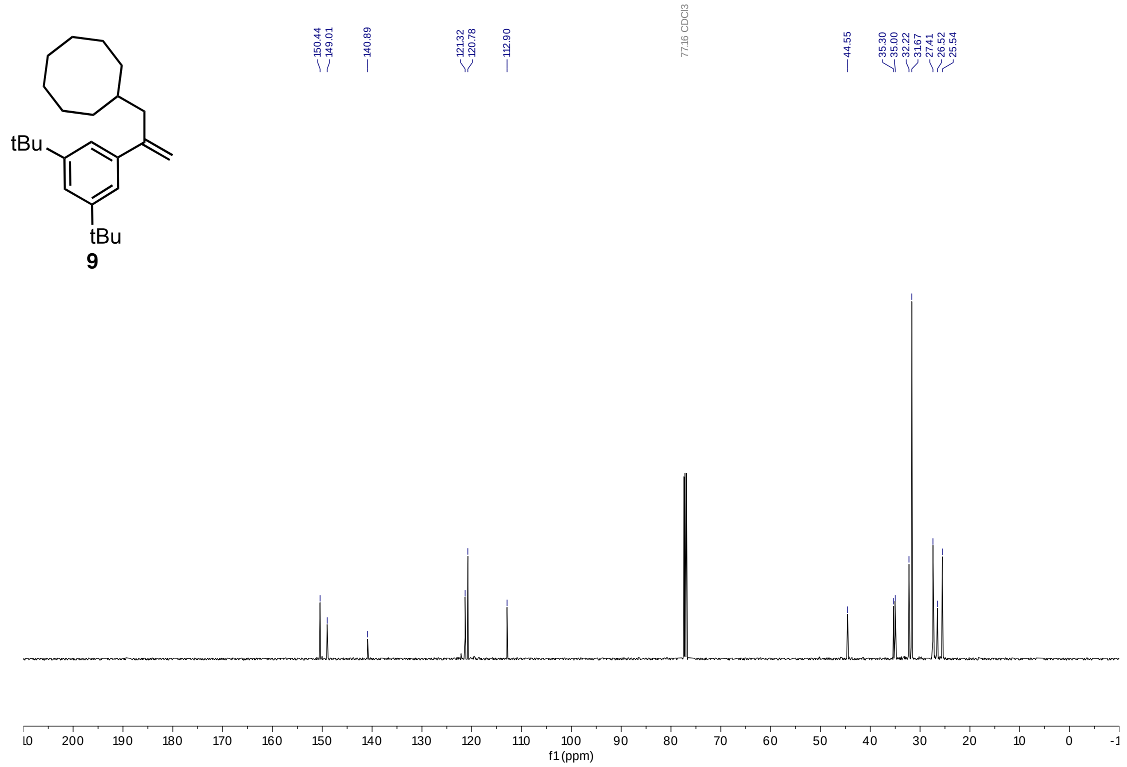

^{1}H NMR (500 MHz, $CDCl_3$)

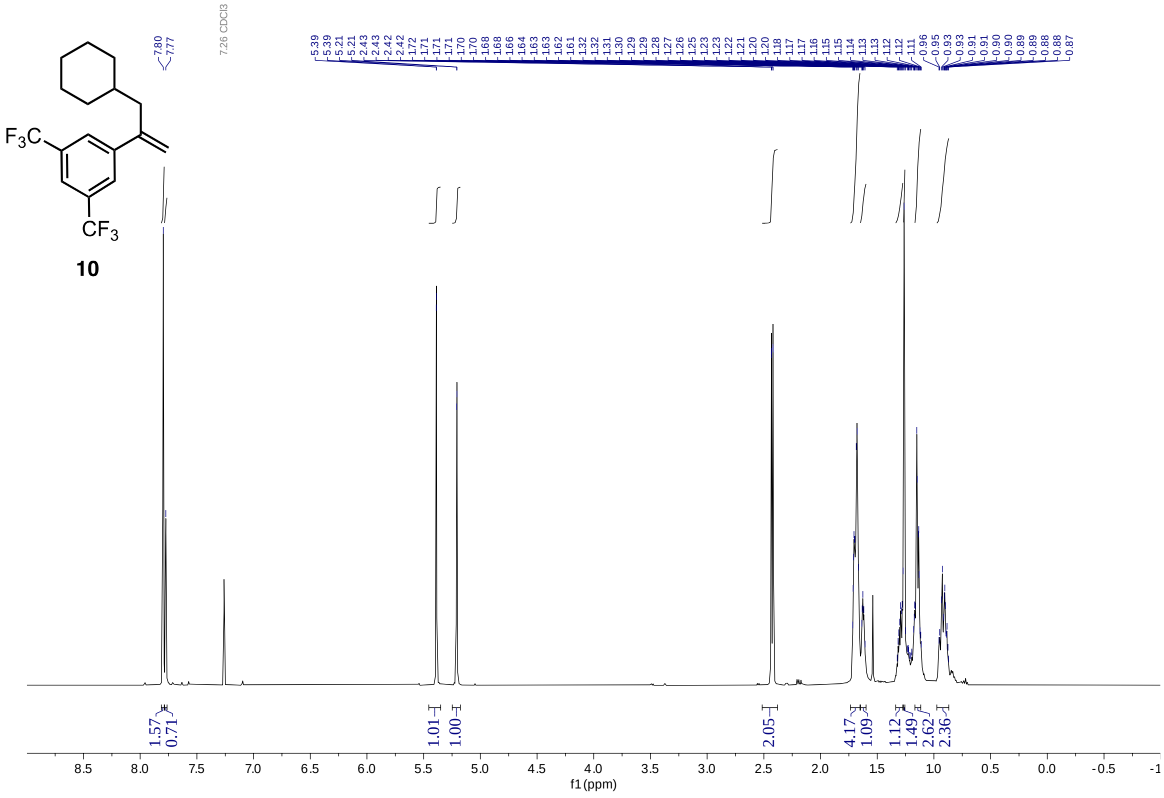

^{13}C NMR (126 MHz, $CDCl_3$)

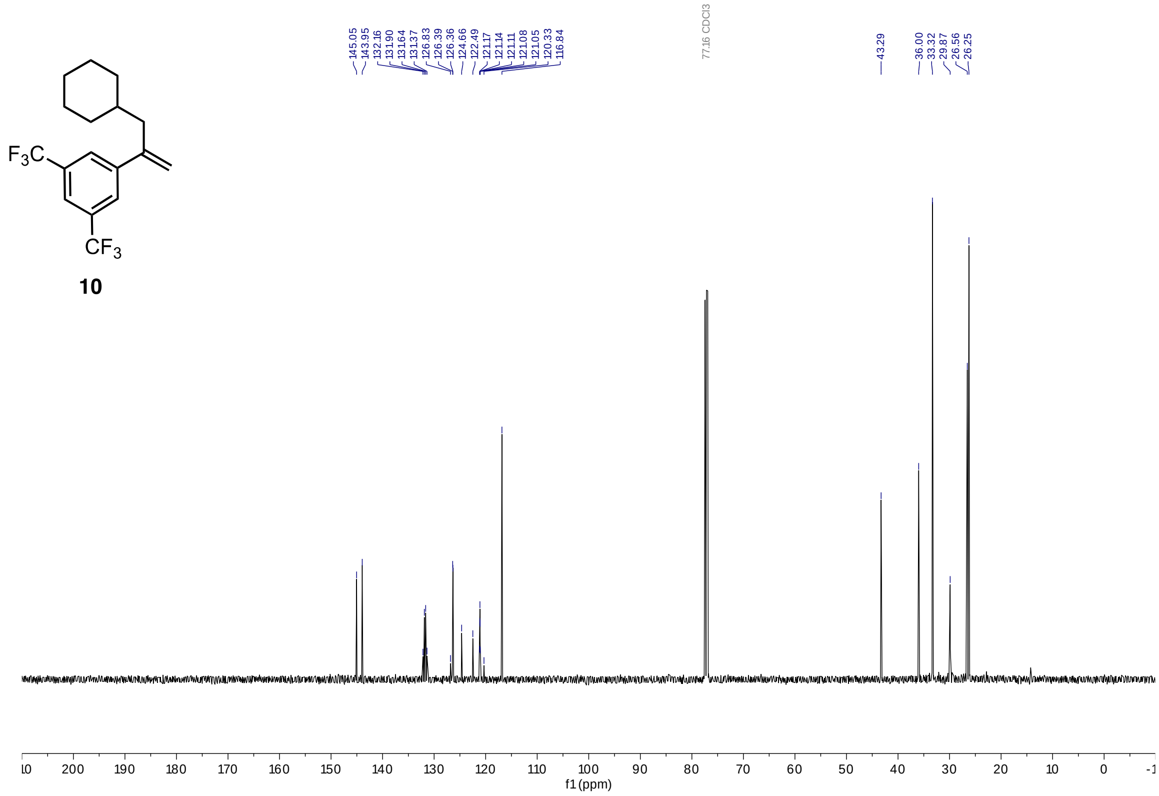

^{19}F NMR (282 MHz, $CDCl_3$)

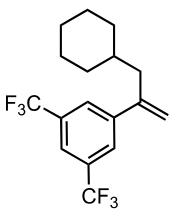

10

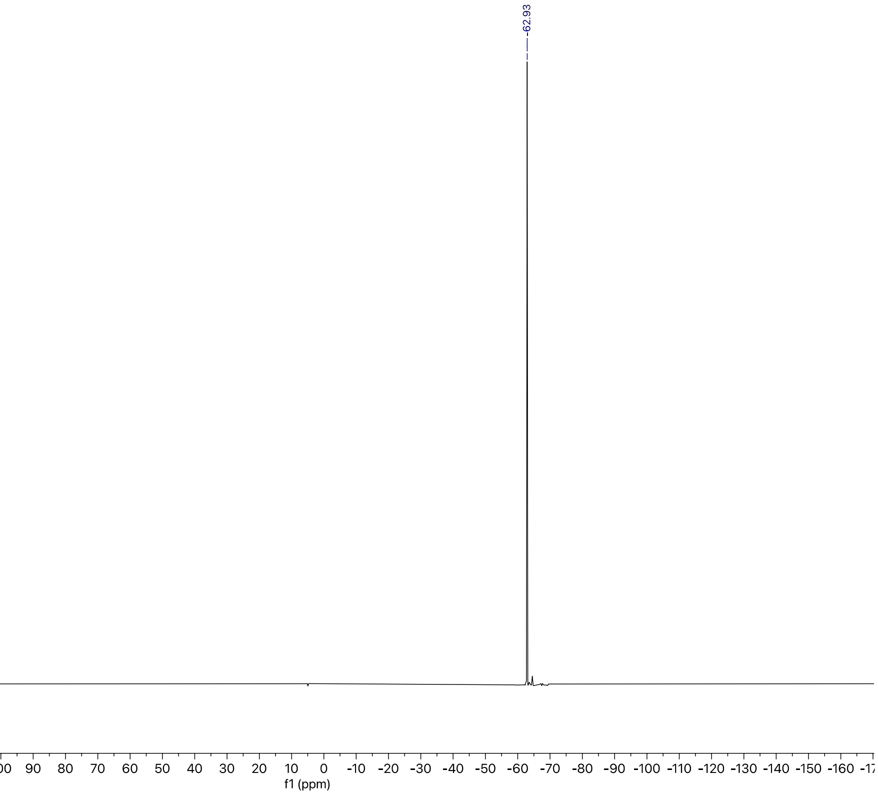

^{1}H NMR (500 MHz, $CDCl_3$)

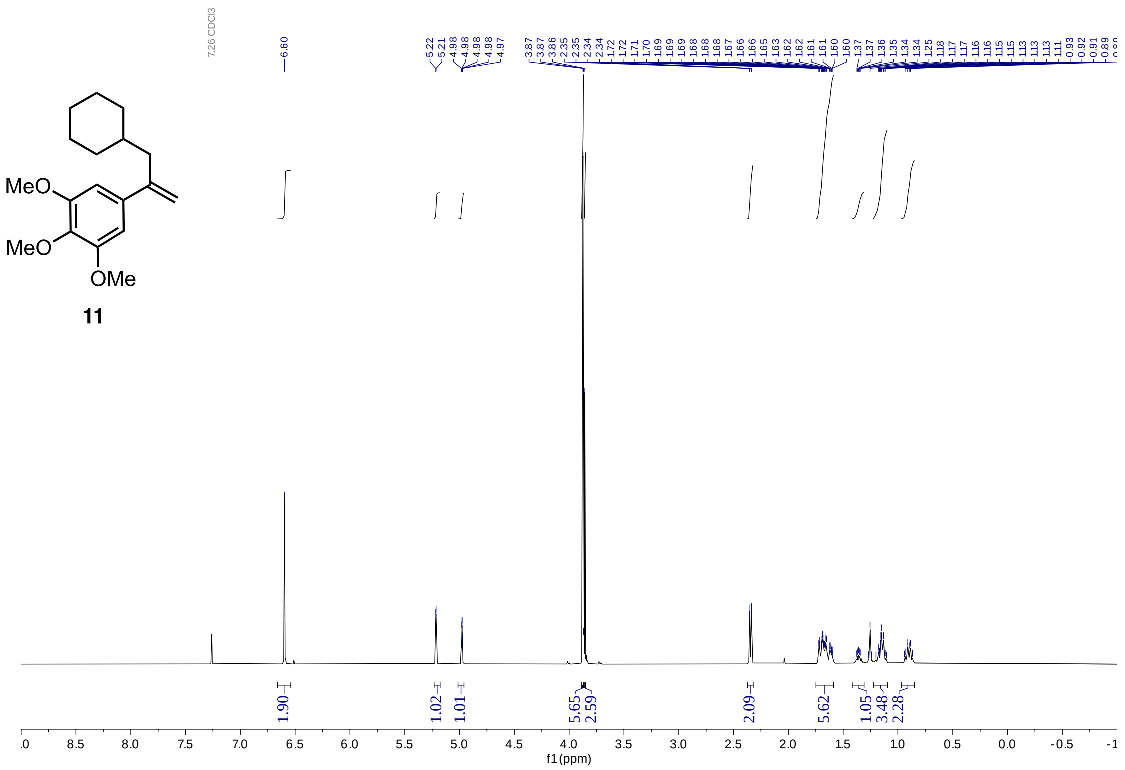

^{13}C NMR (126 MHz, $CDCl_3$)

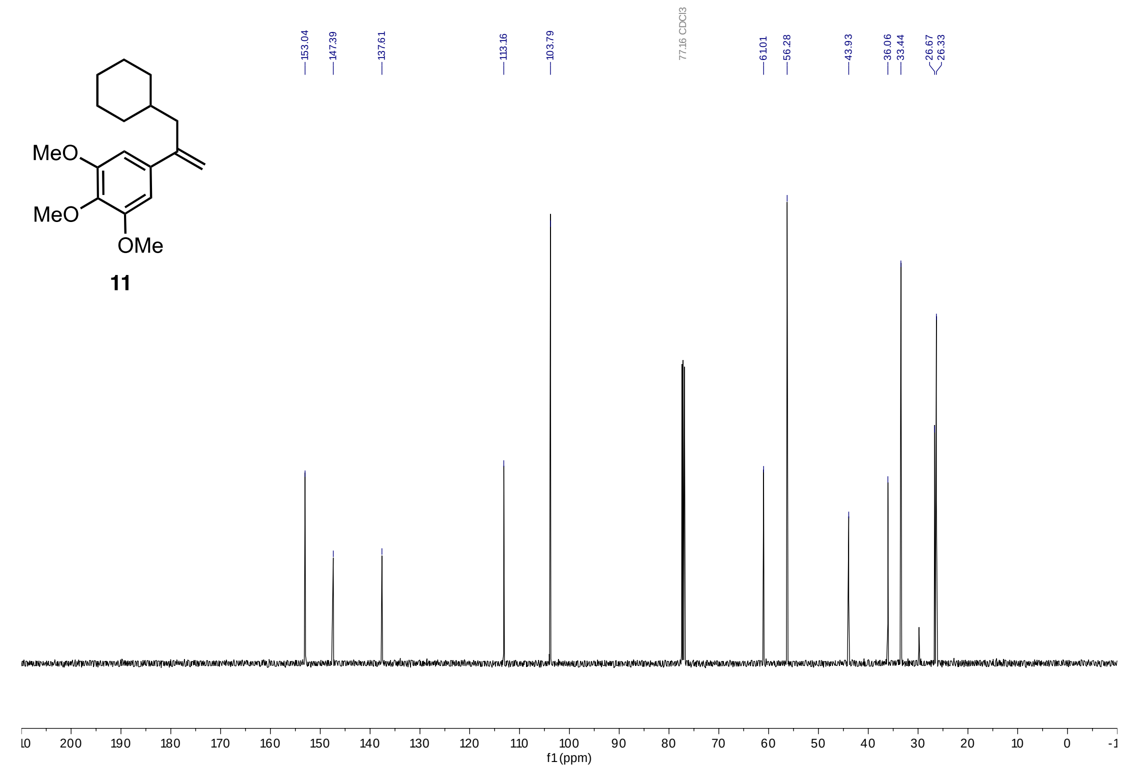

^{1}H NMR (500 MHz, $CDCl_3$)

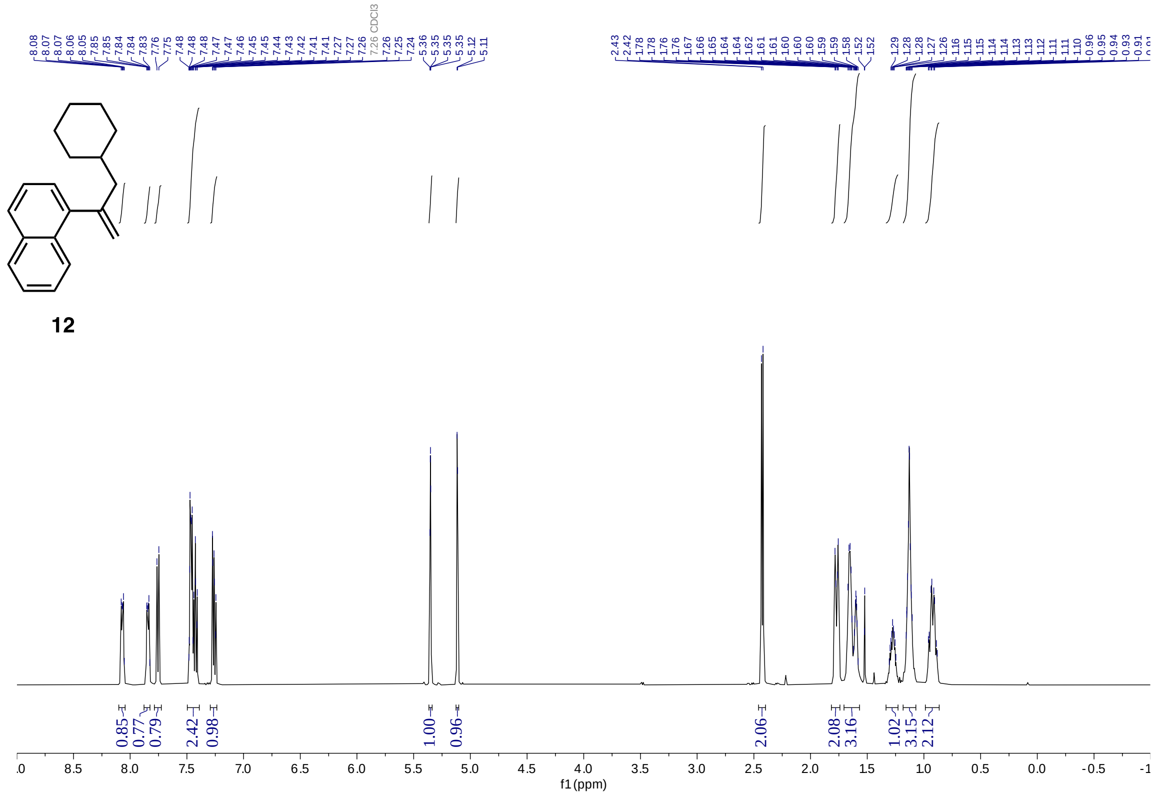

12

^{13}C NMR (126 MHz, $CDCl_3$)

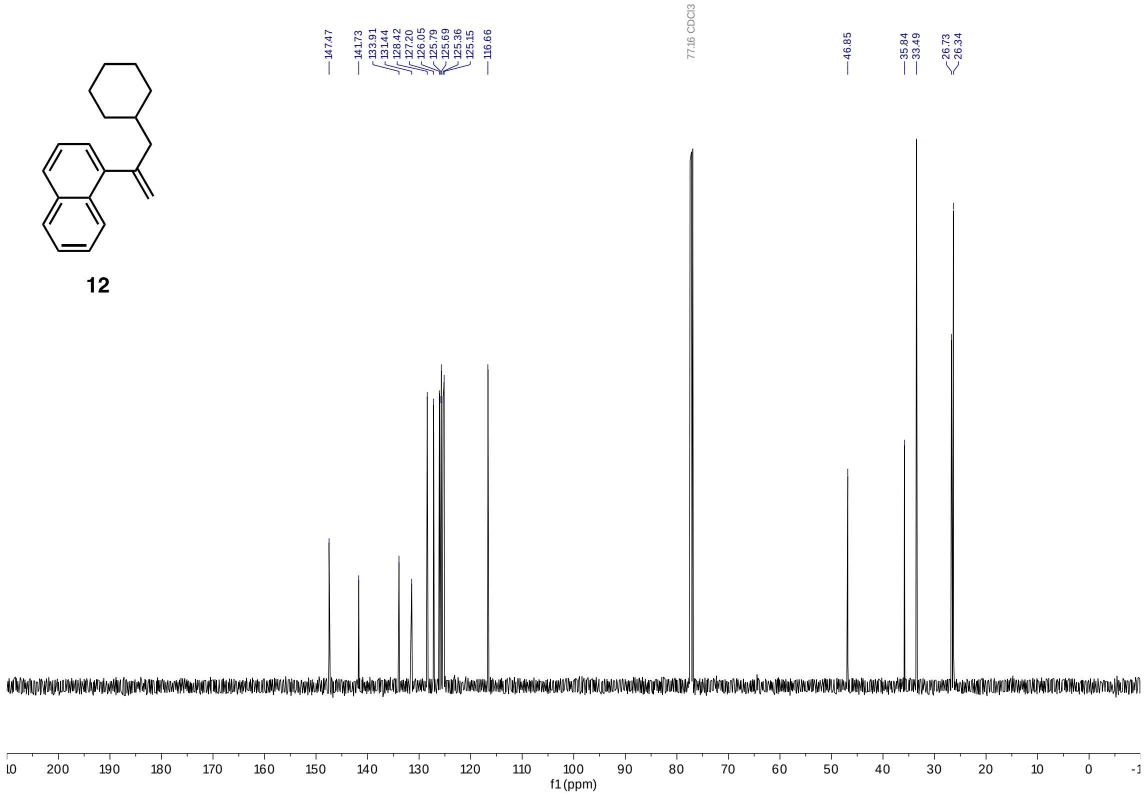

12

^{1}H NMR (500 MHz, $CDCl_3$)

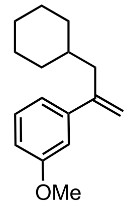

13

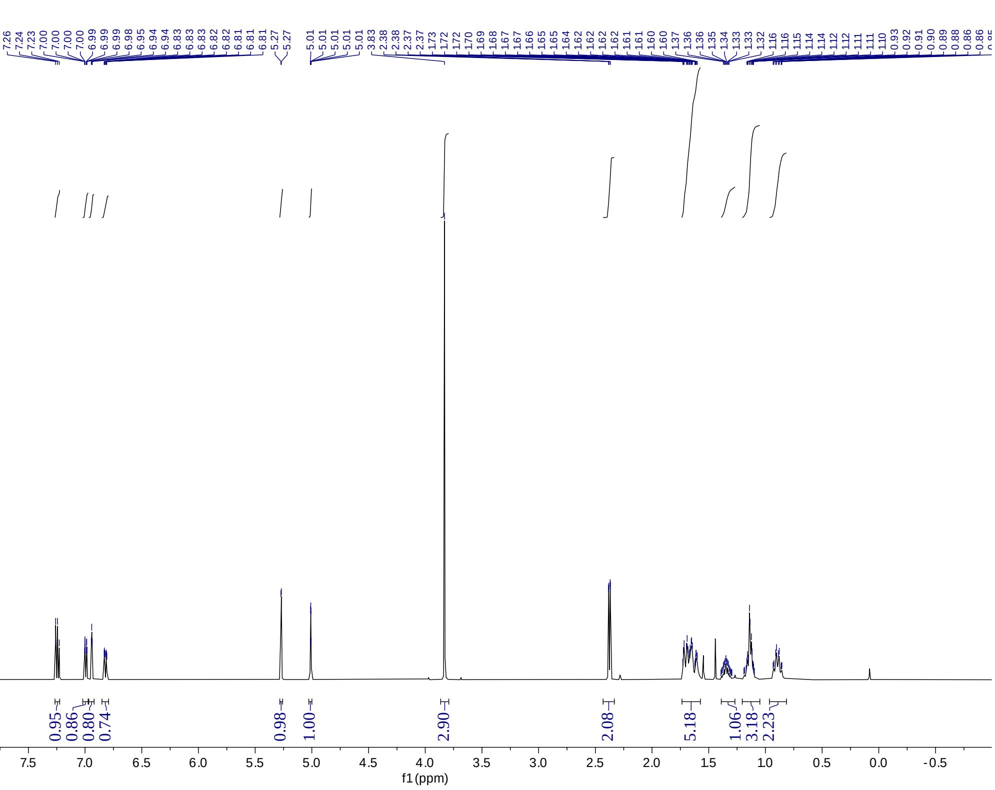

^{13}C NMR (126 MHz, $CDCl_3$)

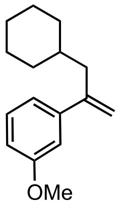

13

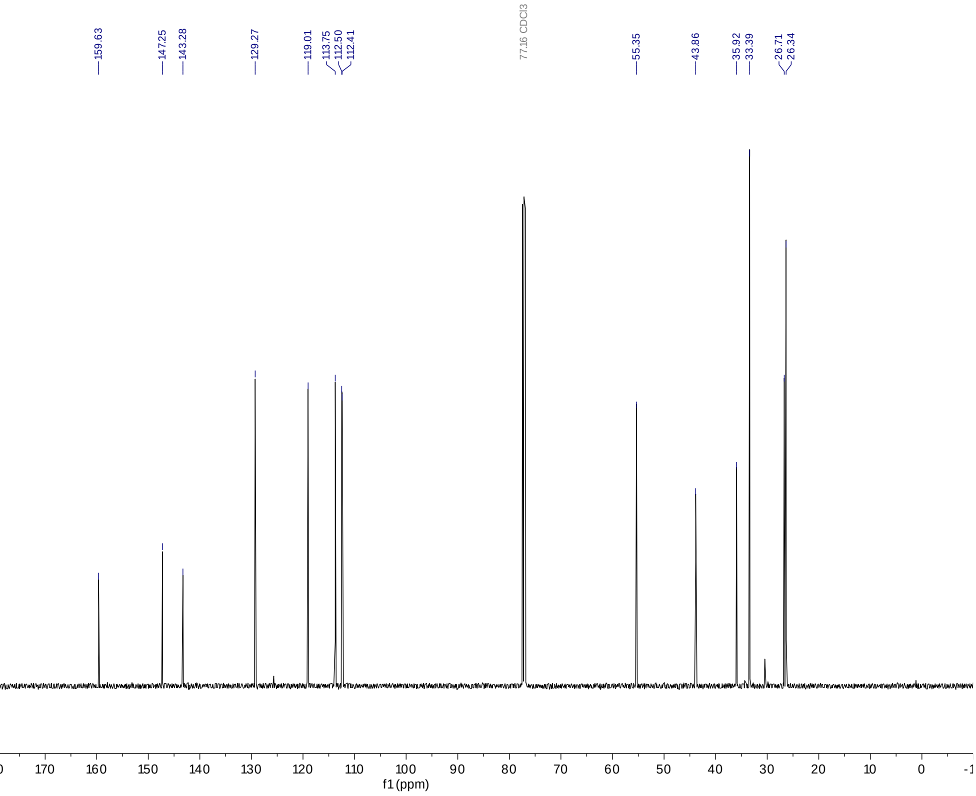

^{1}H NMR (500 MHz, $CDCl_3$)

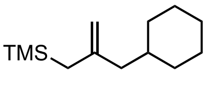

14

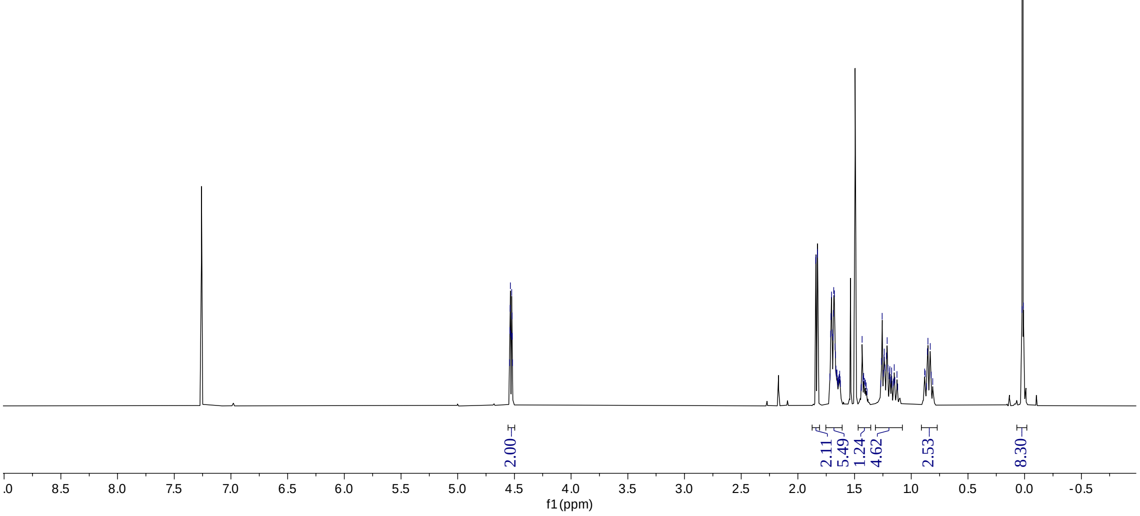

^{13}C NMR (126 MHz, $CDCl_3$)

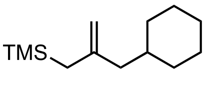

14

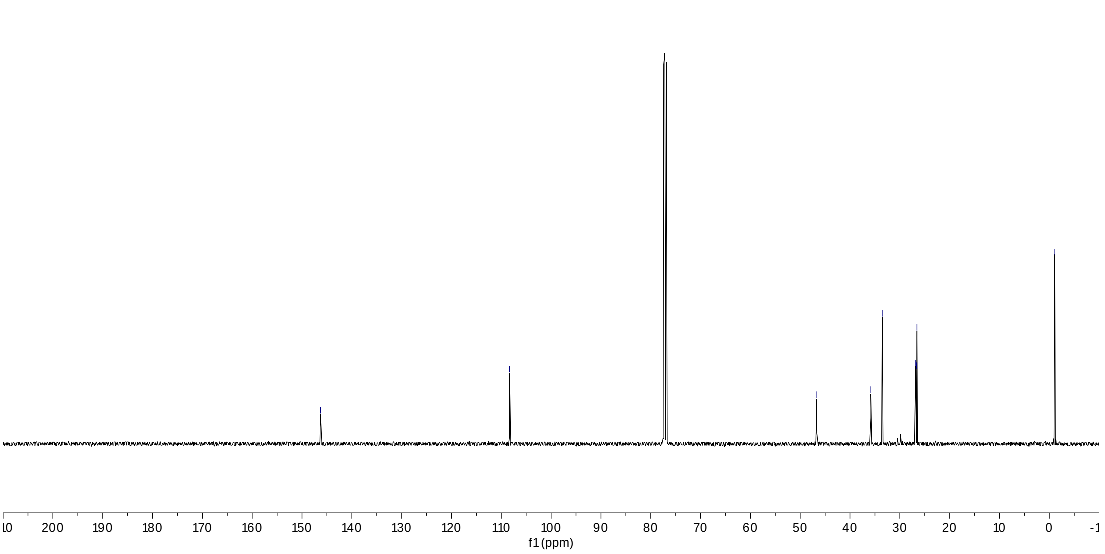

^{1}H NMR (500 MHz, $CDCl_3$)

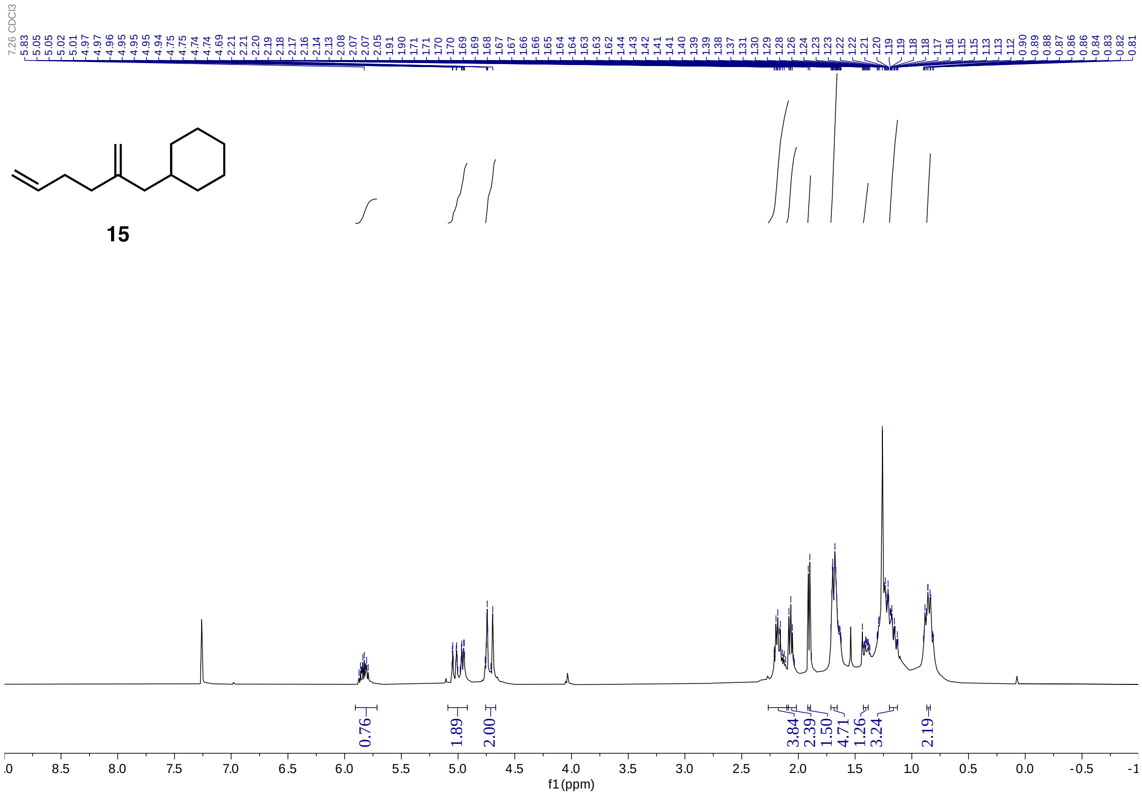

^{13}C NMR (126 MHz, $CDCl_3$)

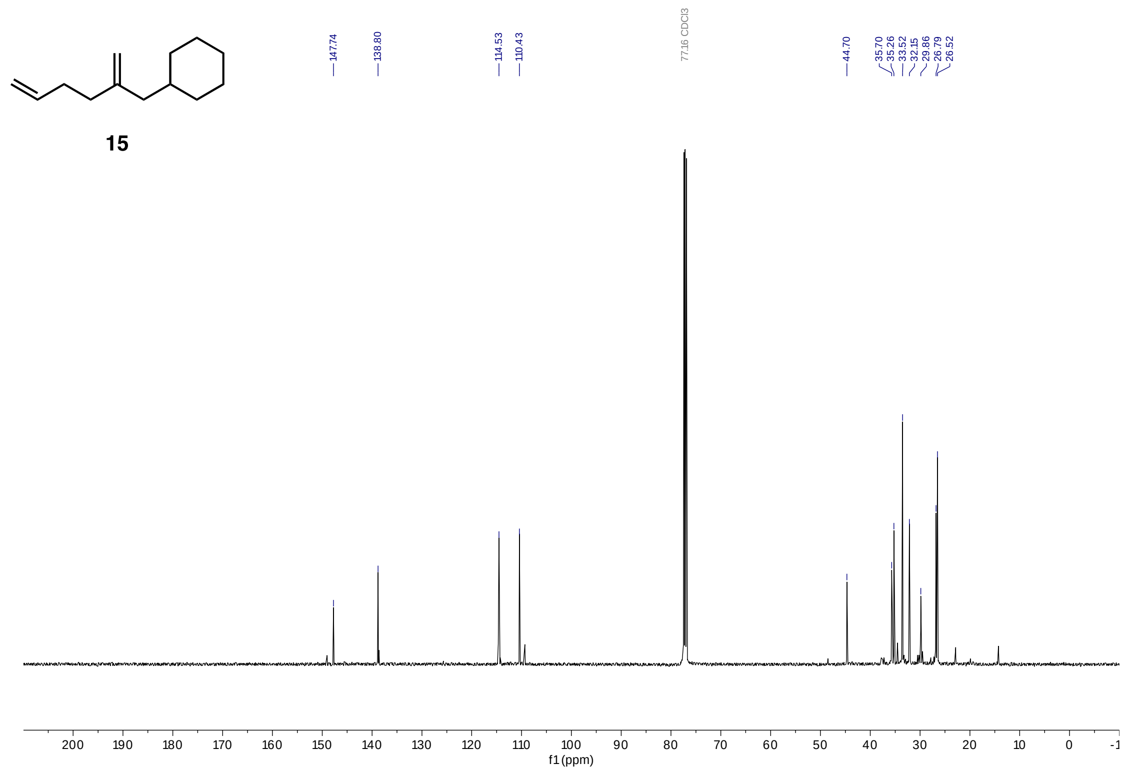

^{1}H NMR (500 MHz, $CDCl_3$)

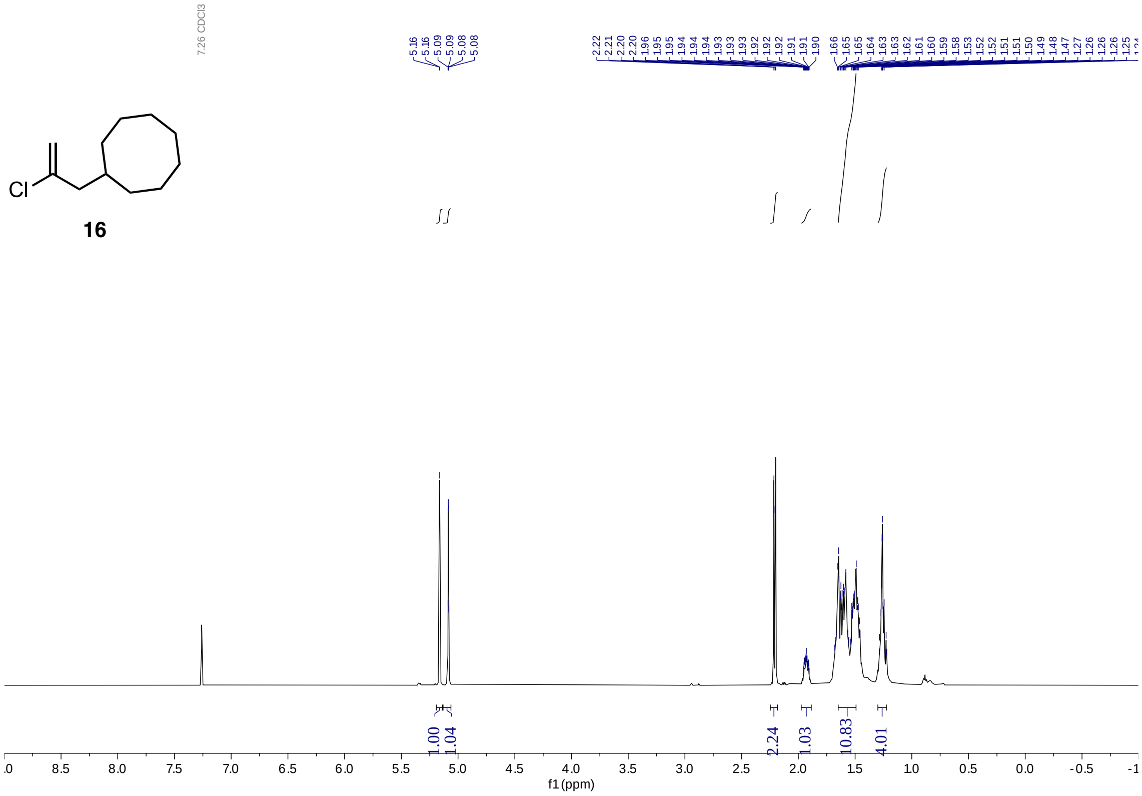

^{13}C NMR (126 MHz, $CDCl_3$)

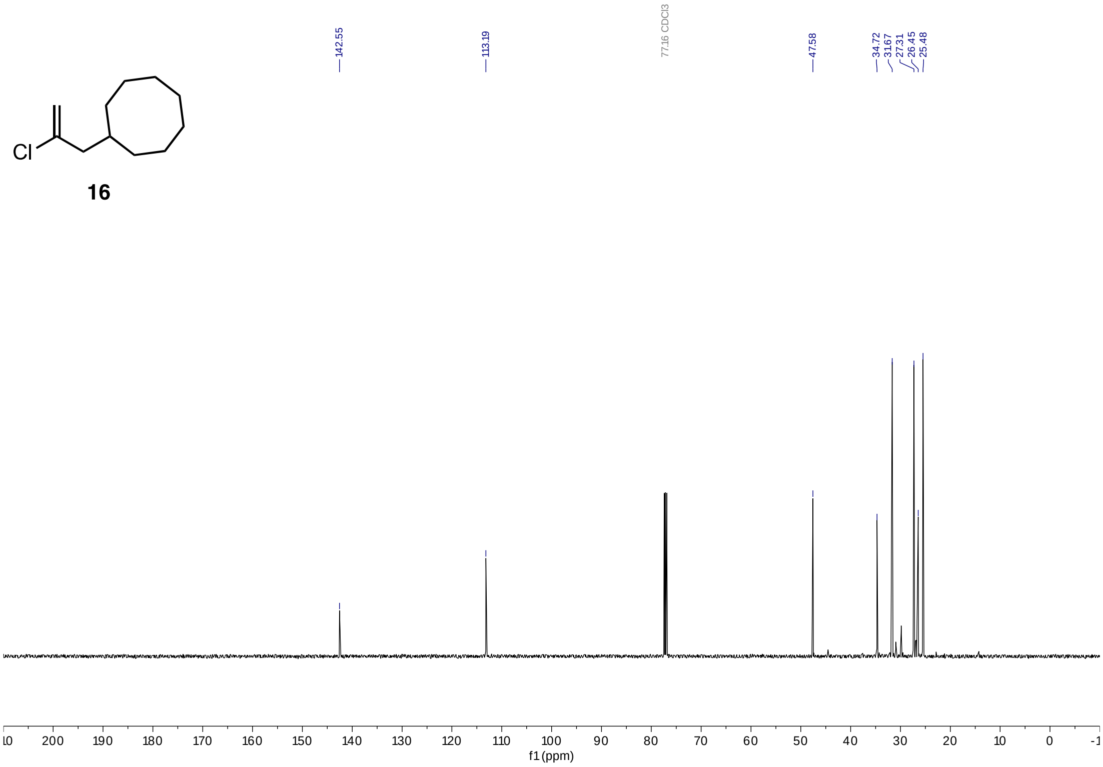

^{1}H NMR (500 MHz, $CDCl_3$)

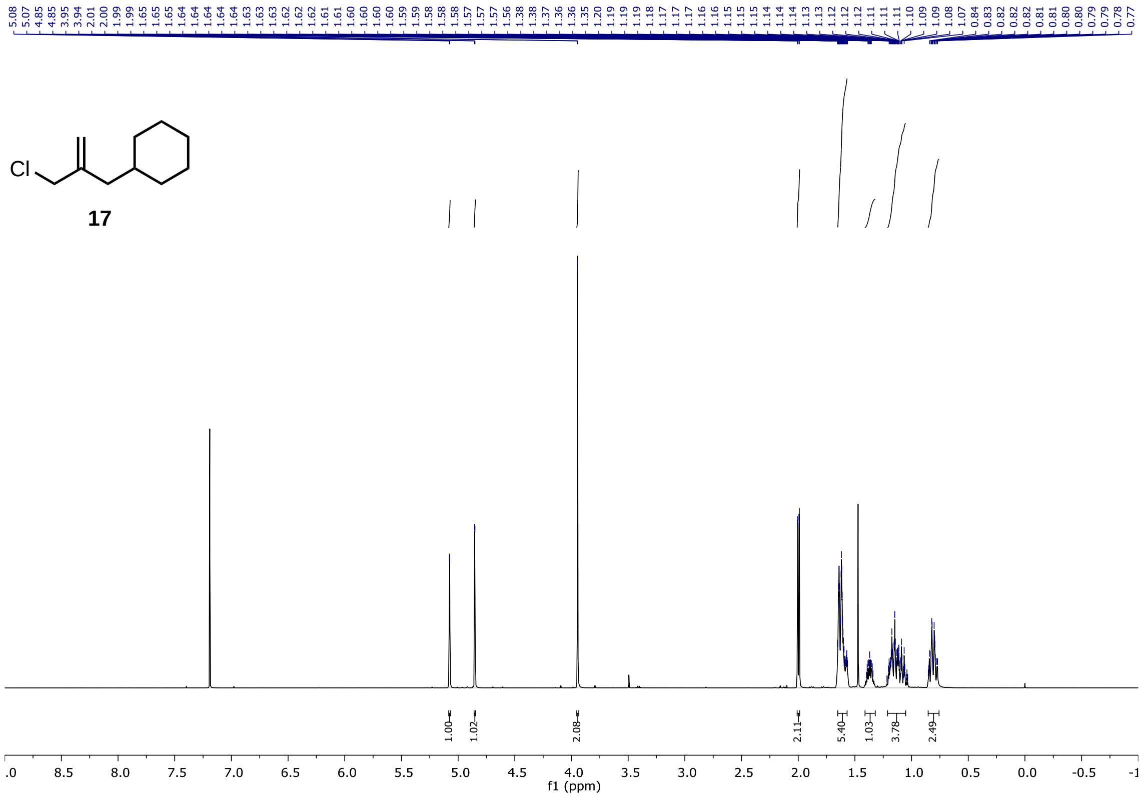

^{13}C NMR (126 MHz, $CDCl_3$)

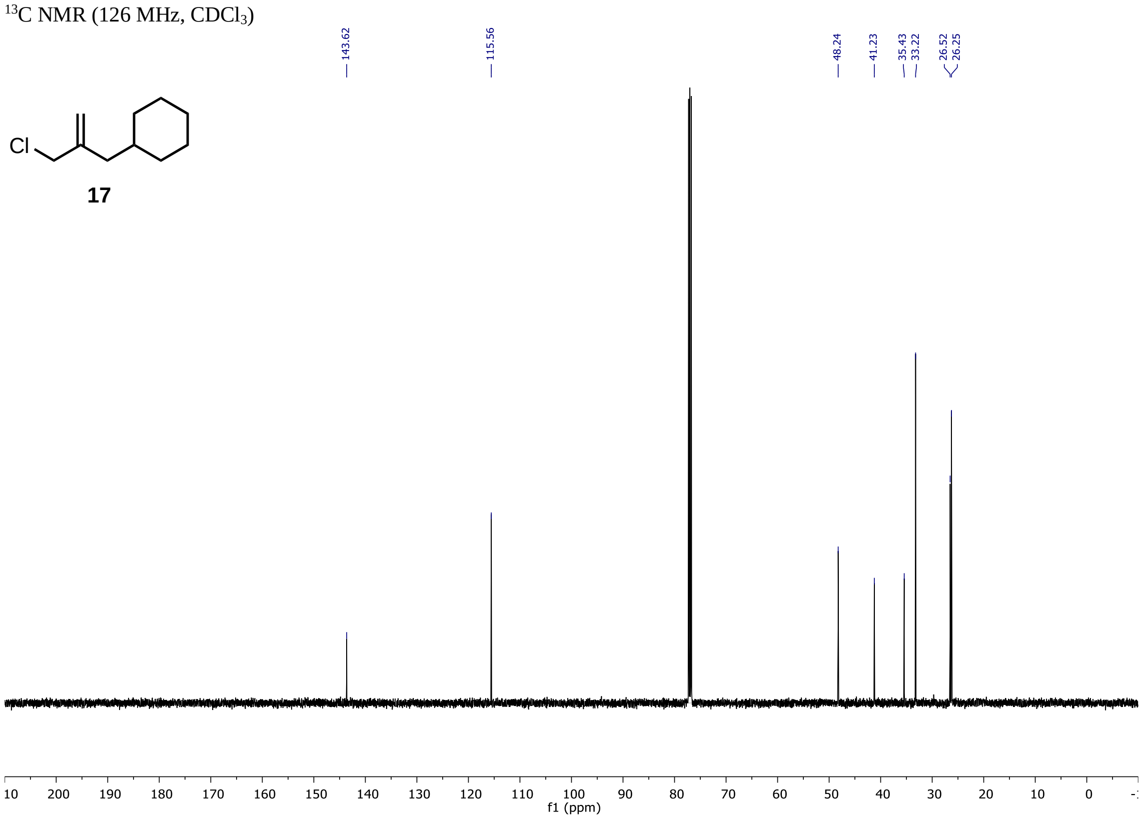

^{1}H NMR (500 MHz, $CDCl_3$)

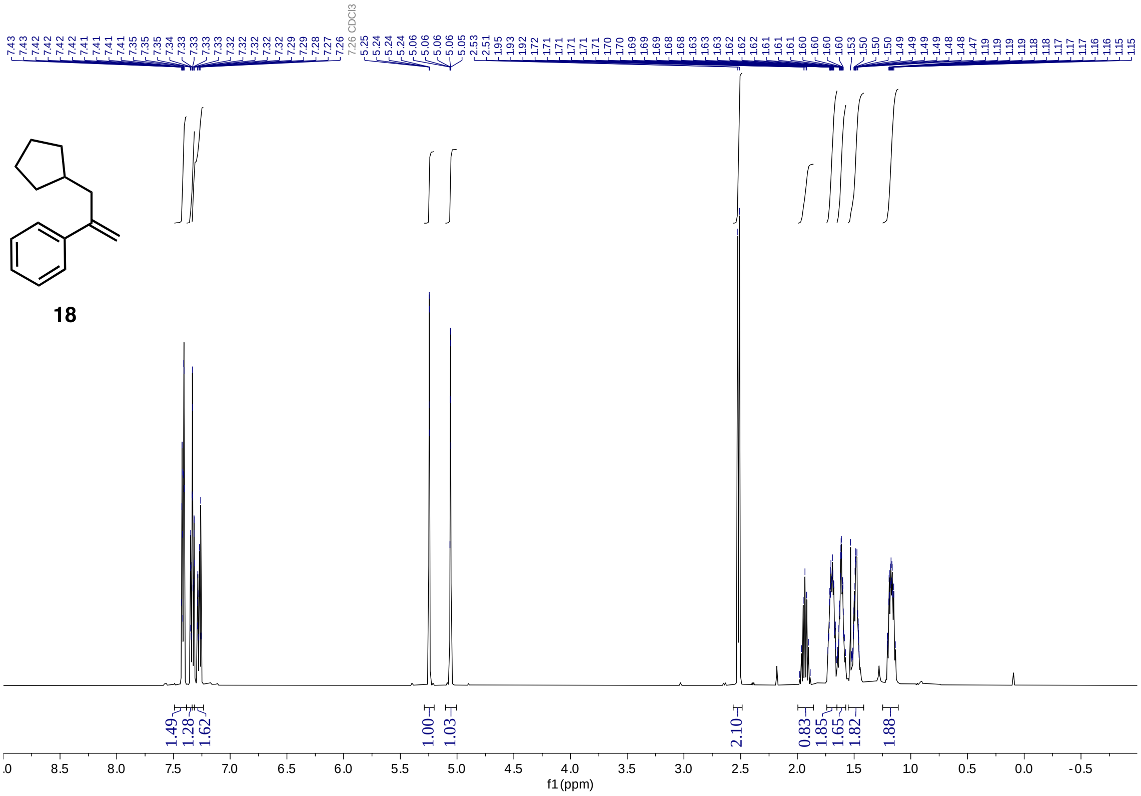

18

^{13}C NMR (126 MHz, $CDCl_3$)

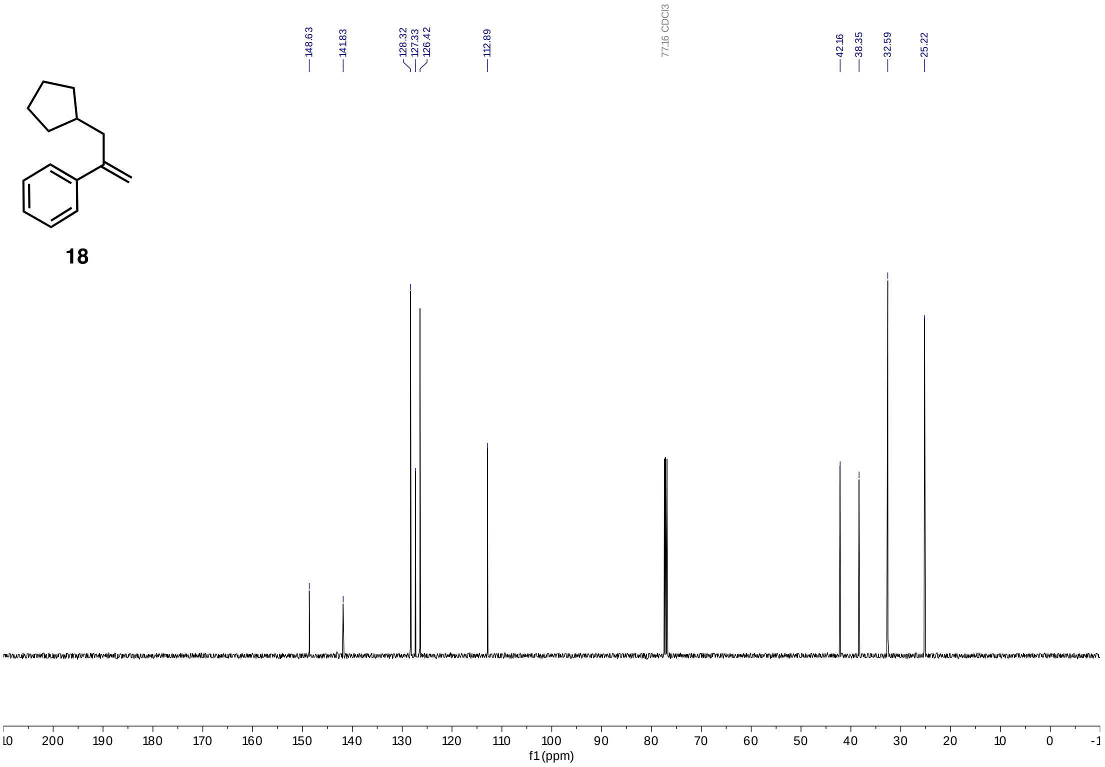

18

^{1}H NMR (500 MHz, $CDCl_3$)

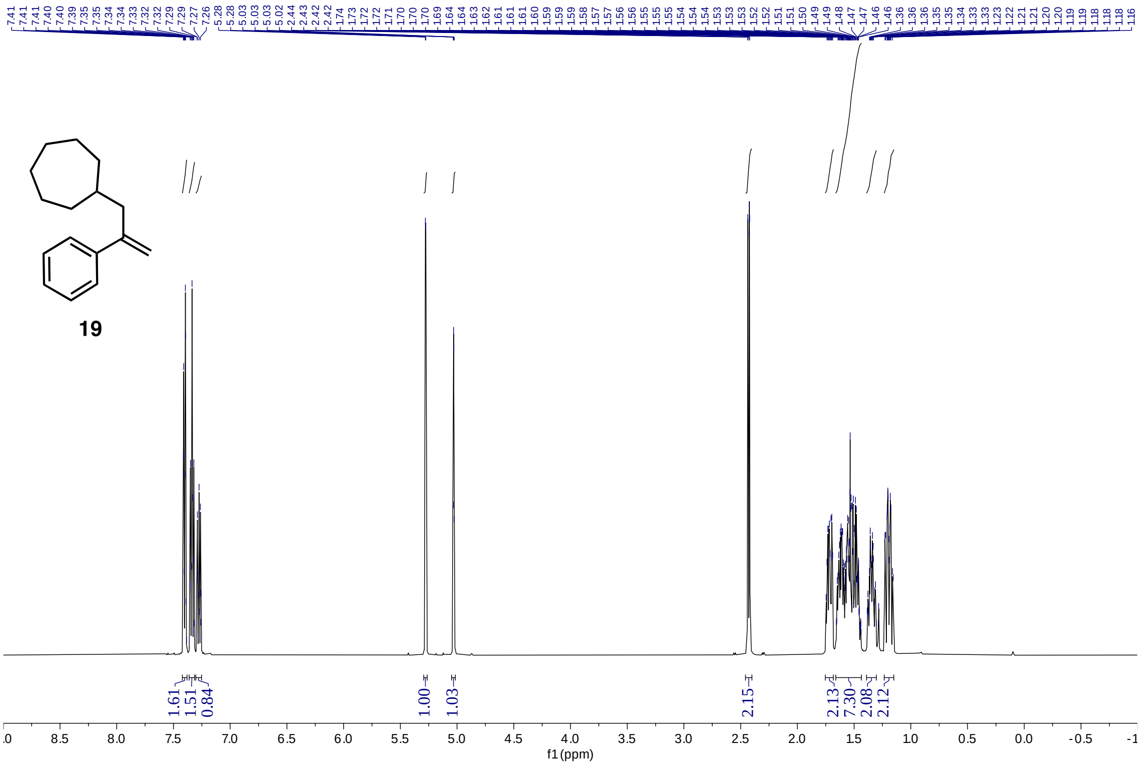

^{13}C NMR (126 MHz, $CDCl_3$)

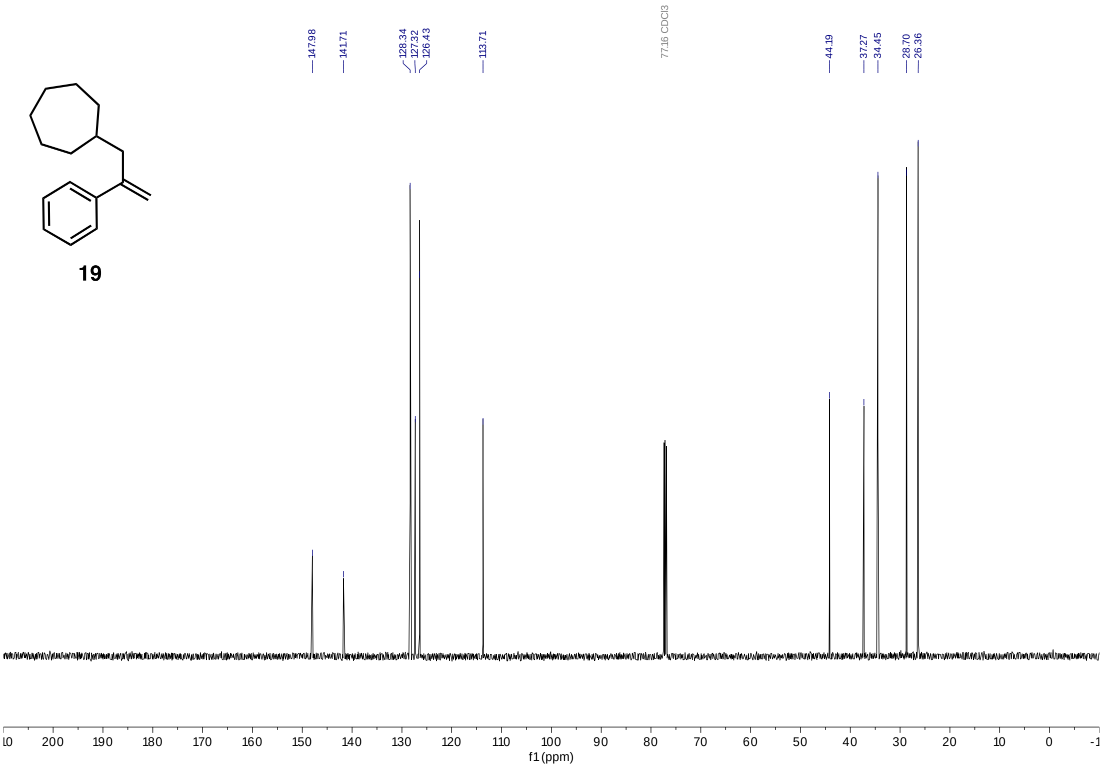

^{1}H NMR (500 MHz, $CDCl_3$)

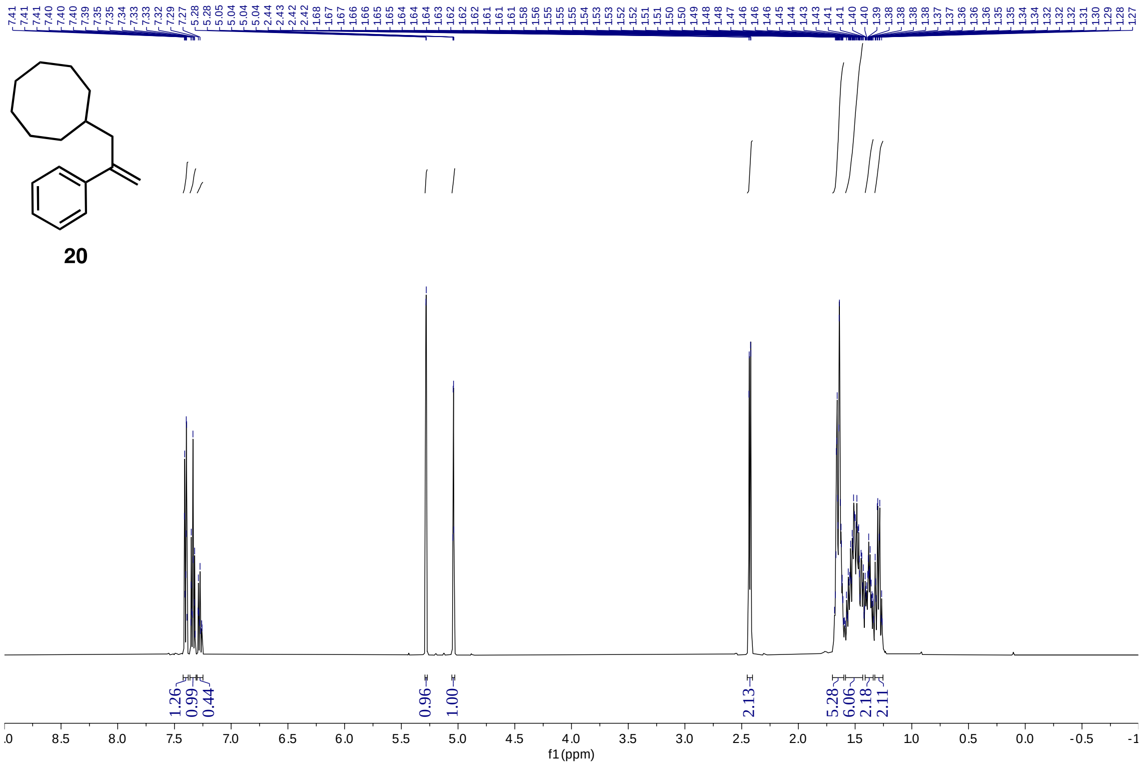

^{13}C NMR (126 MHz, $CDCl_3$)

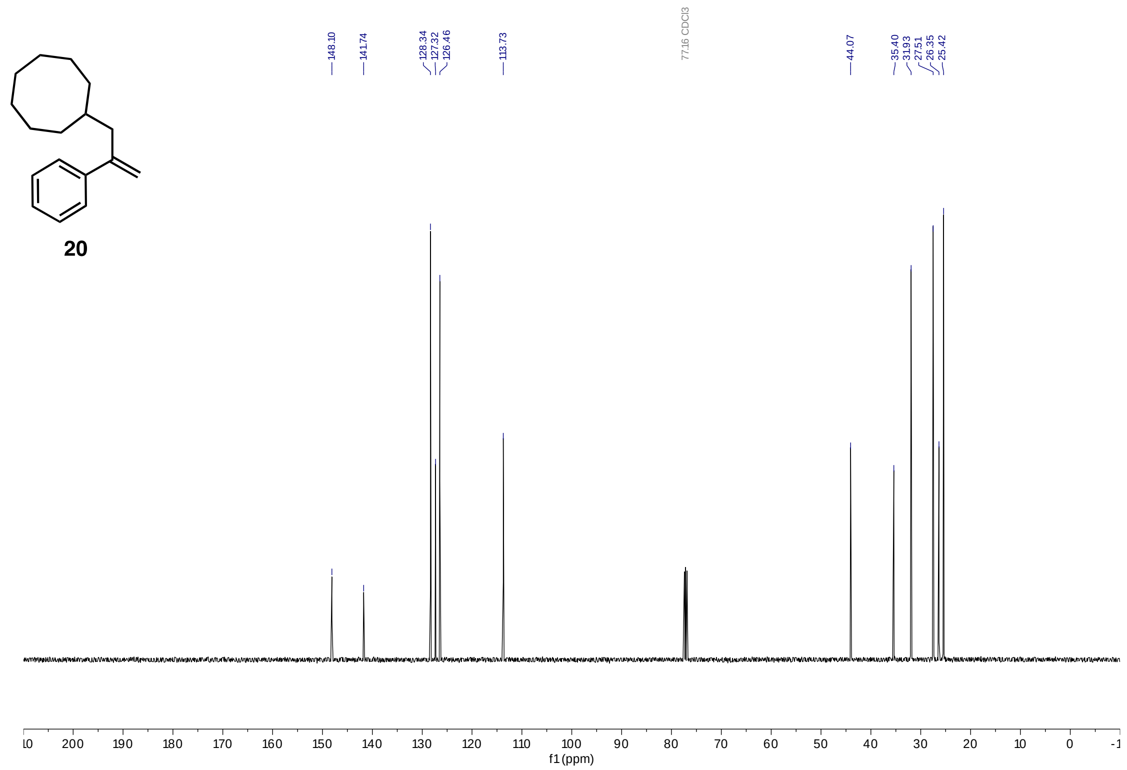

^{1}H NMR (500 MHz, $CDCl_3$)

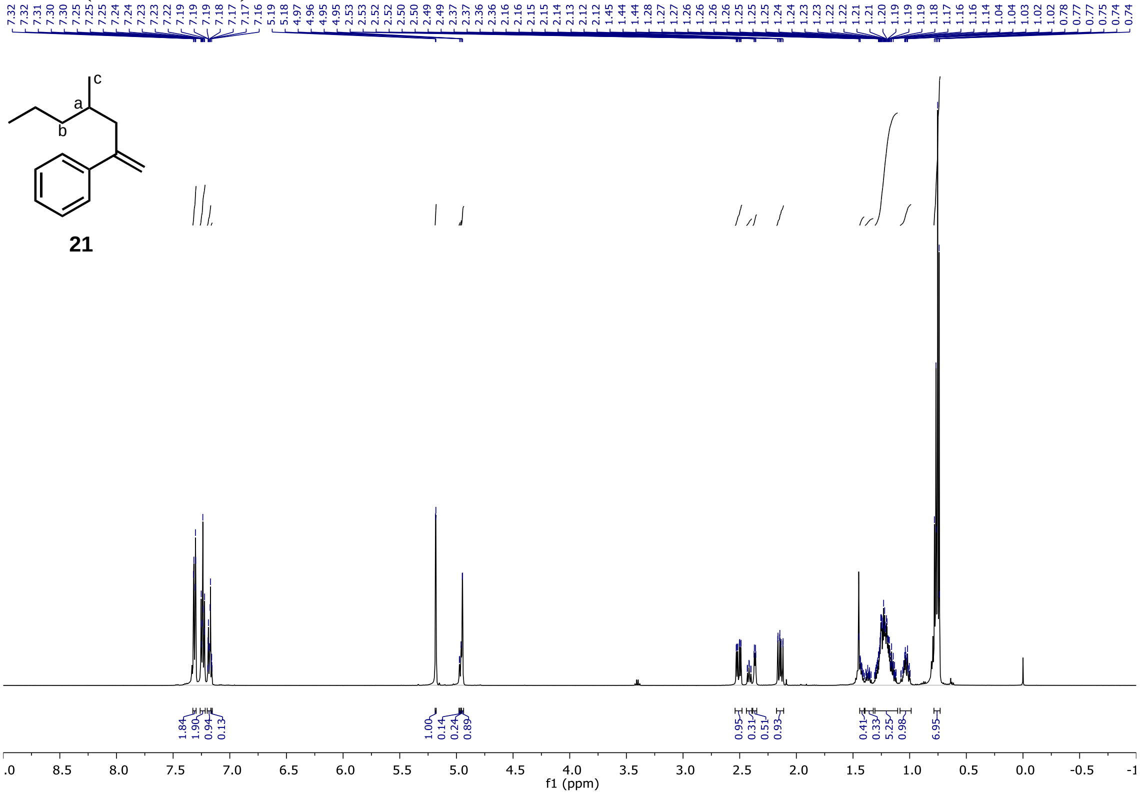

21

^{13}C NMR (126 MHz, $CDCl_3$)

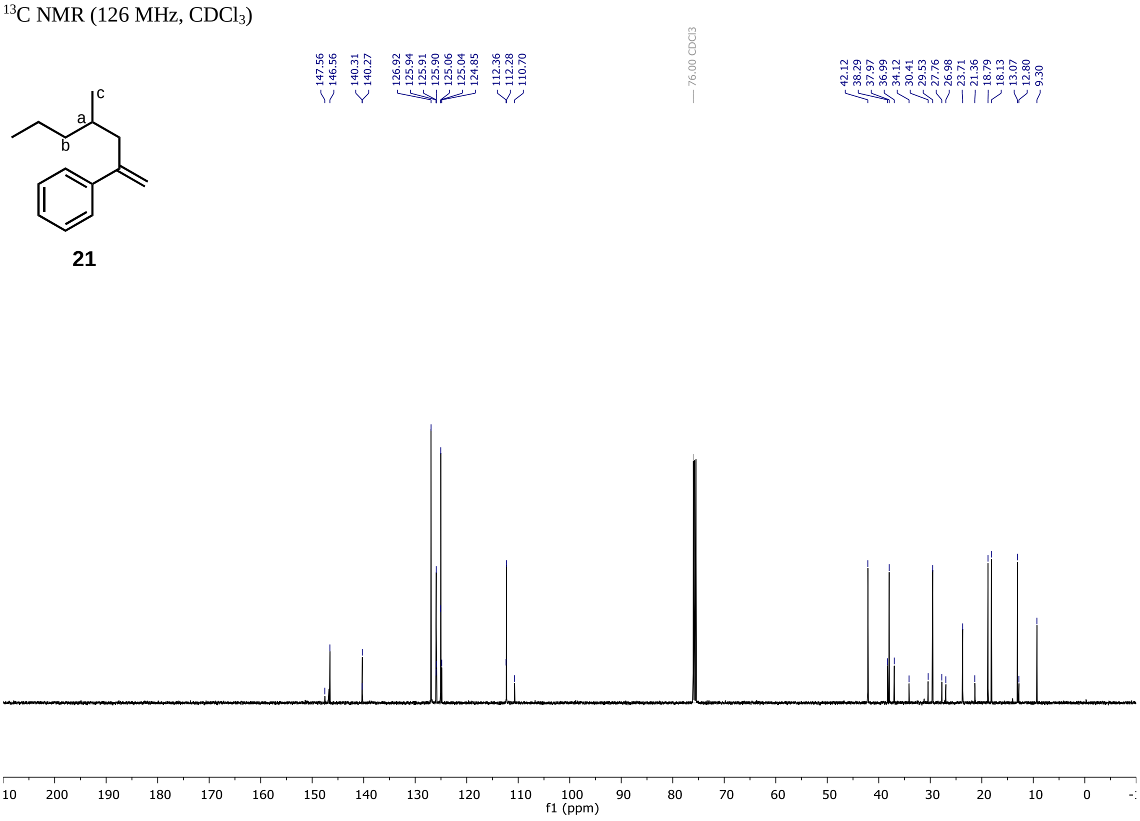

21

^{1}H NMR (500 MHz, $CDCl_3$)

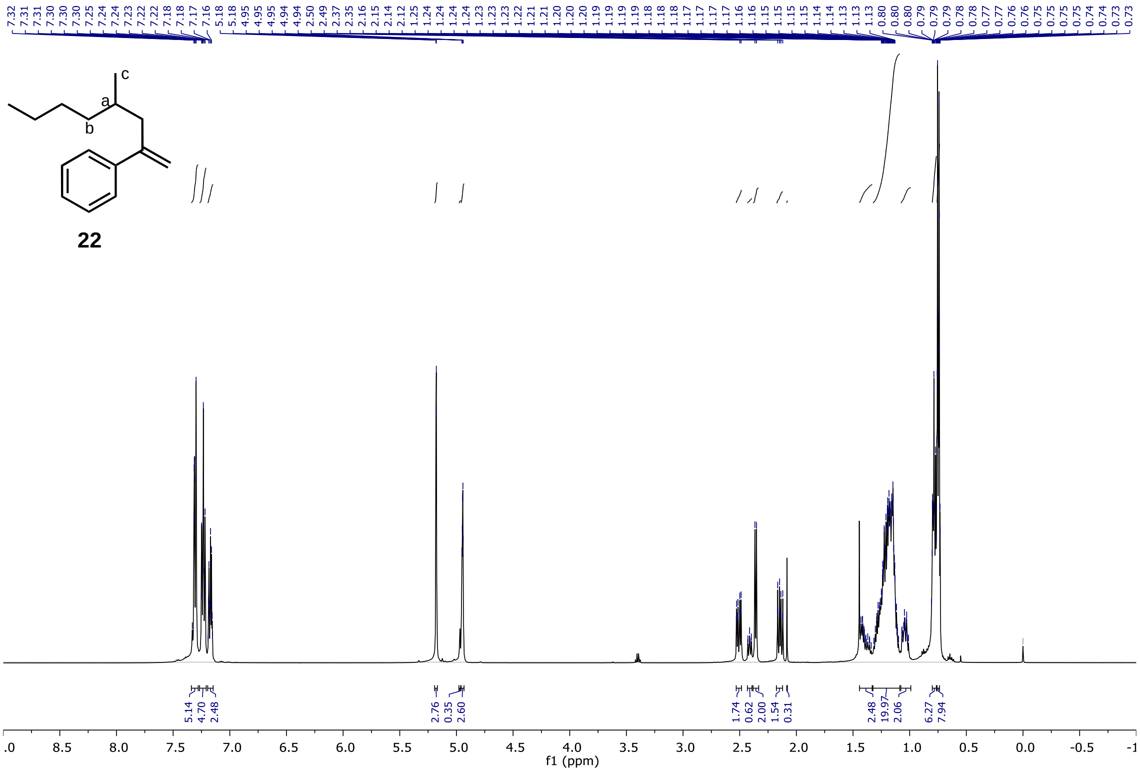

^{13}C NMR (126 MHz, $CDCl_3$)

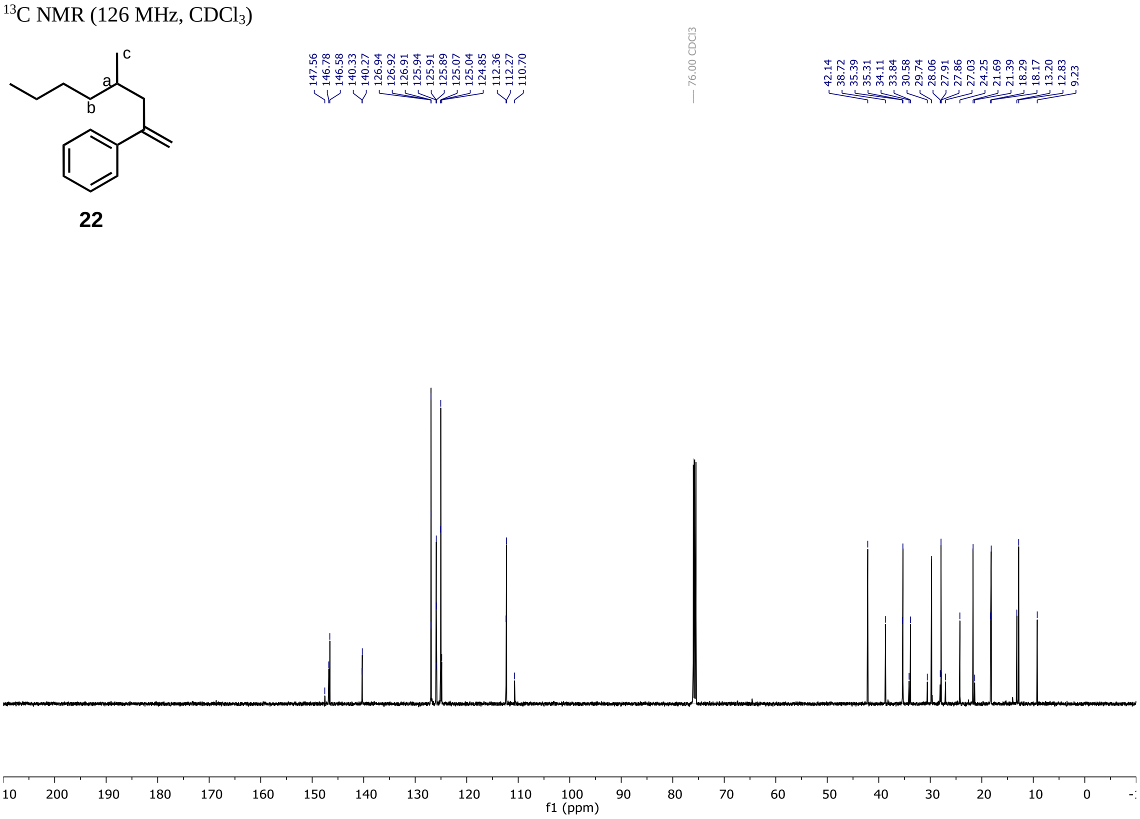

^{1}H NMR (500 MHz, $CDCl_3$)

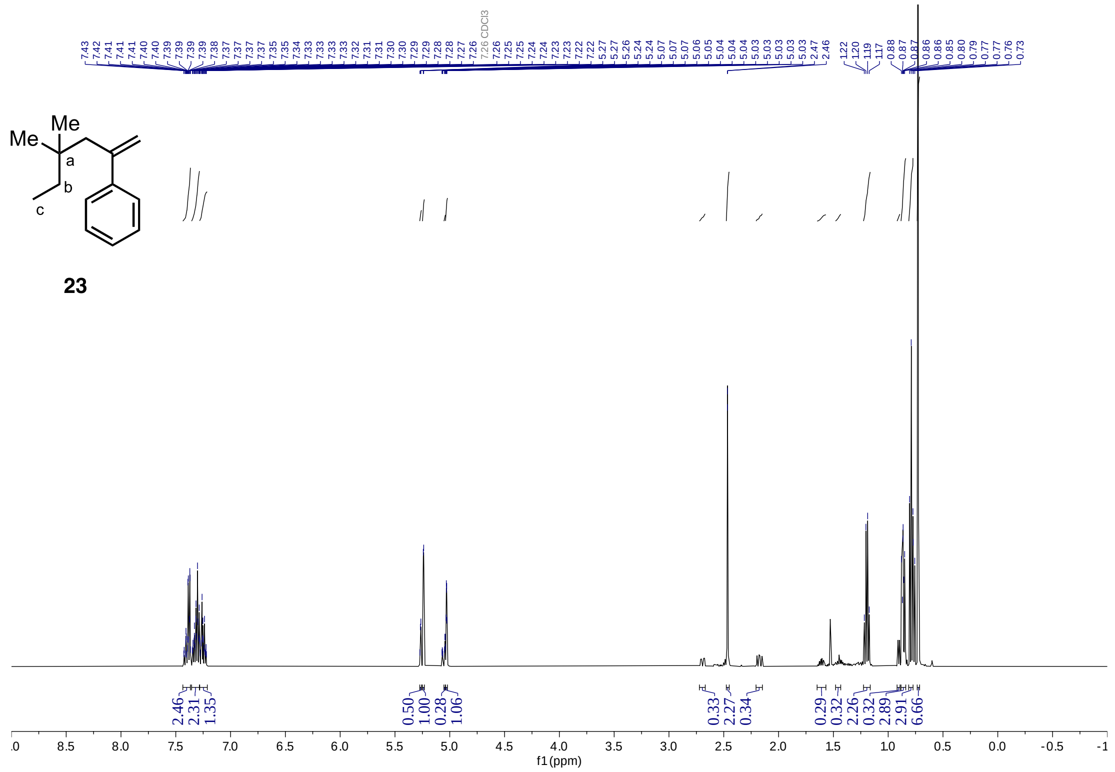

^{13}C NMR (126 MHz, $CDCl_3$)

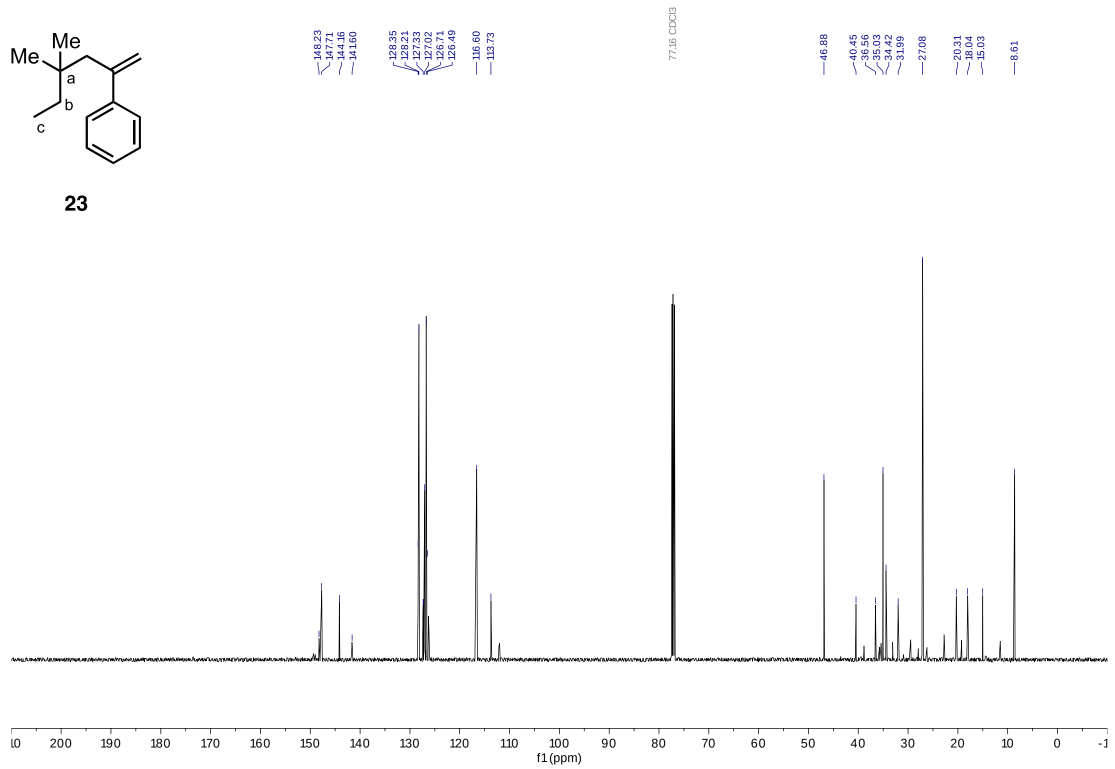

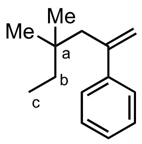

23

COSY (500 MHz, $CDCl_3$)

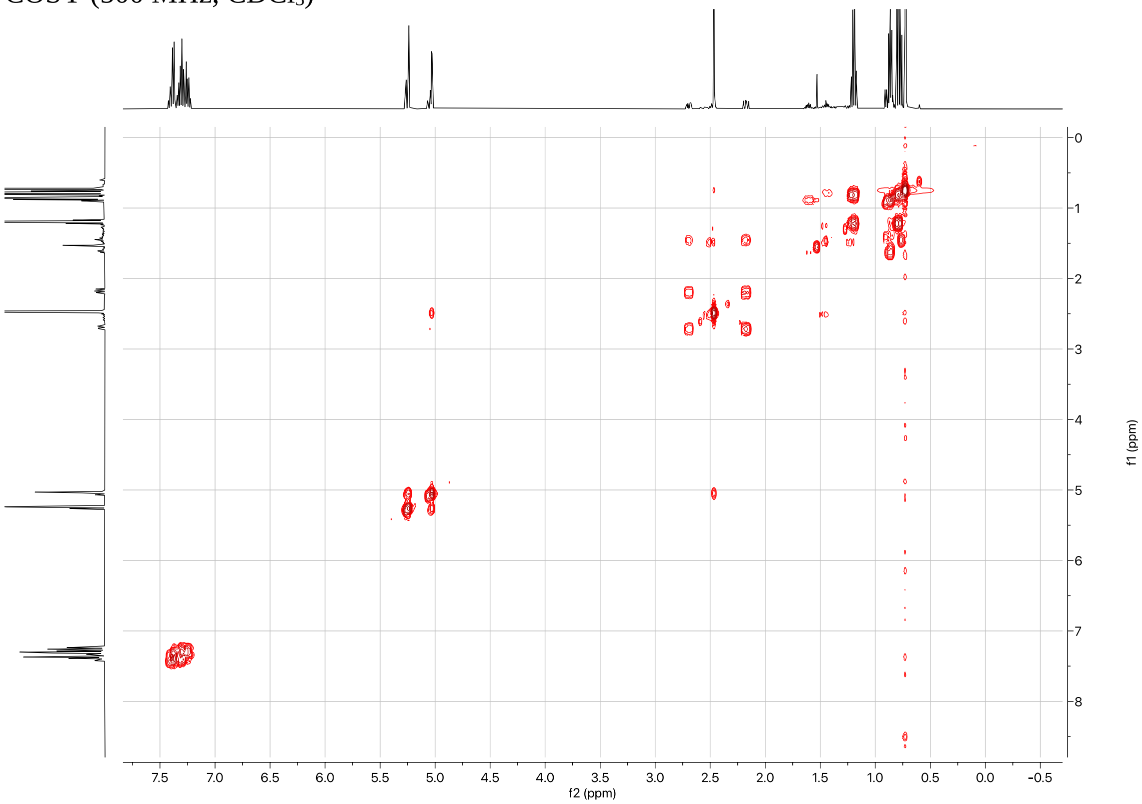

HSQC (500, 126 MHz, $CDCl_3$)

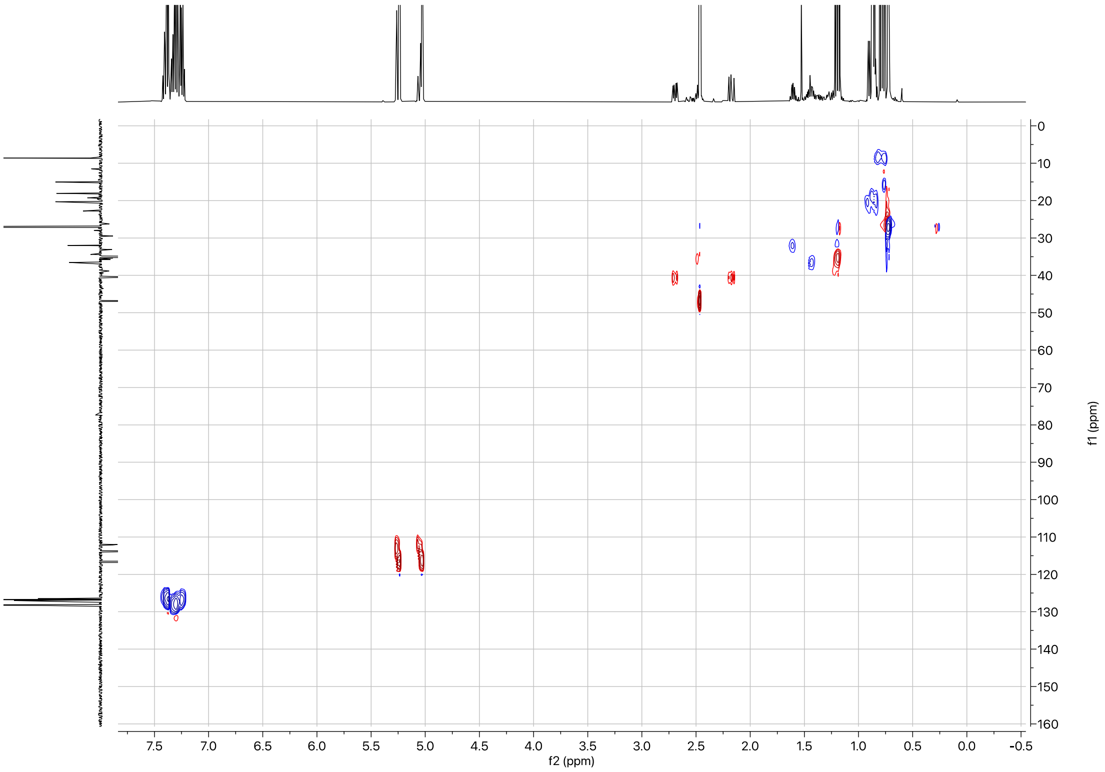

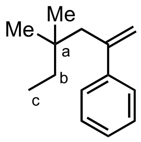

23

HMBC (500, 126 MHz, $CDCl_3$)

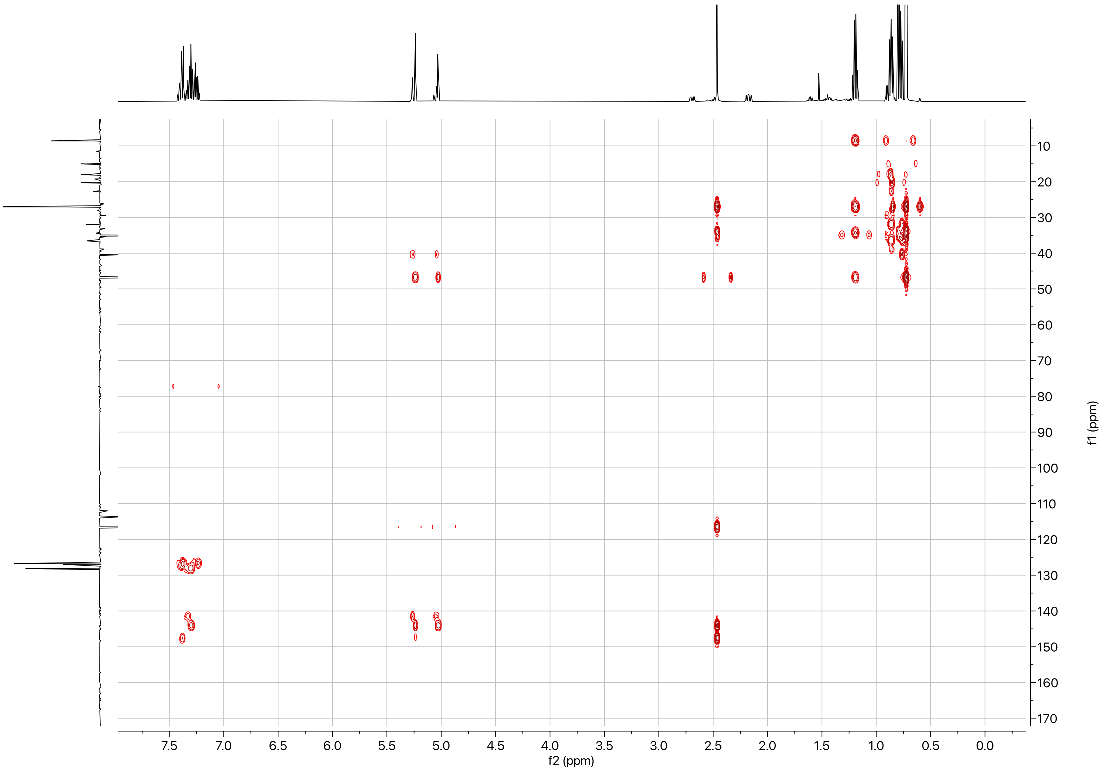

^{1}H NMR (500 MHz, $CDCl_3$)

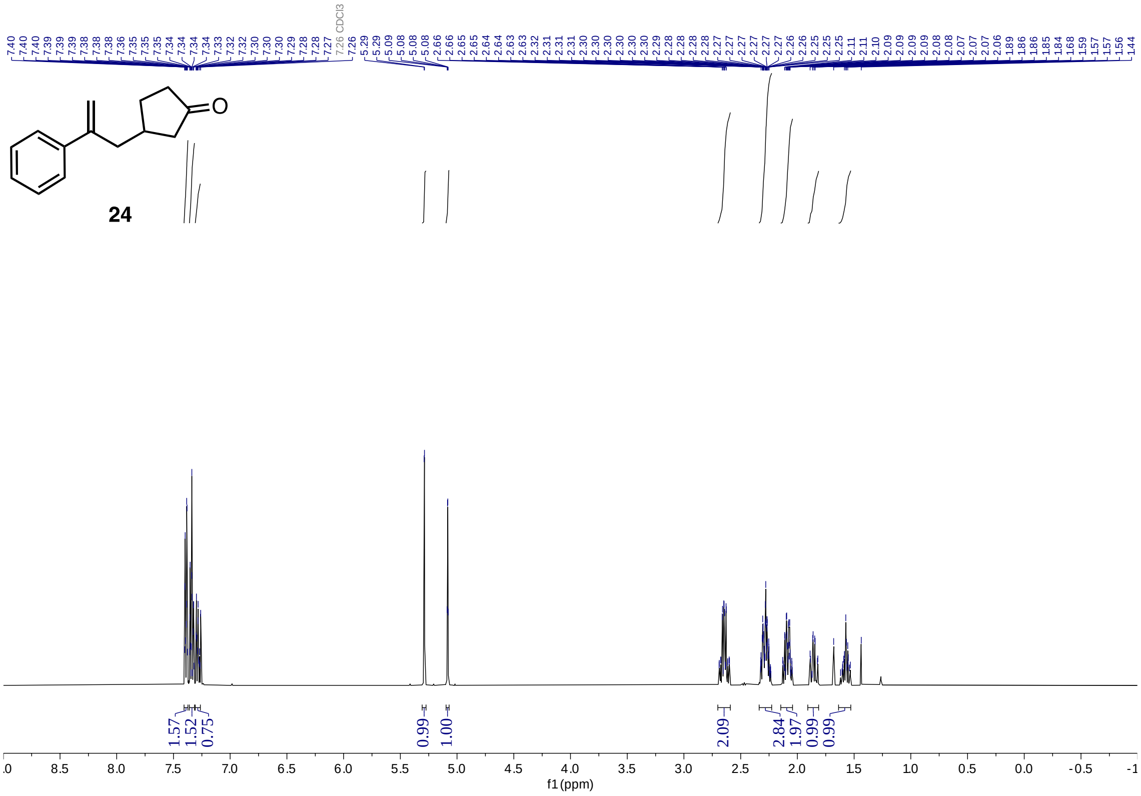

^{13}C NMR (126 MHz, $CDCl_3$)

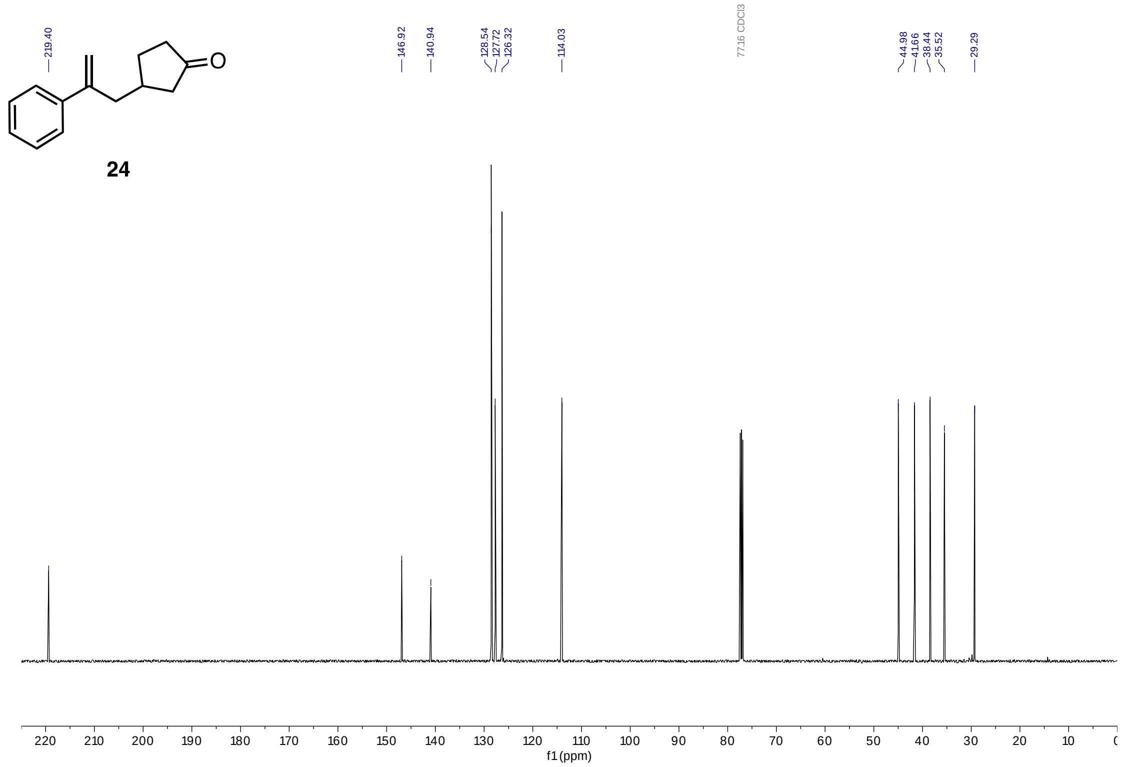

^{1}H NMR (500 MHz, $CDCl_3$)

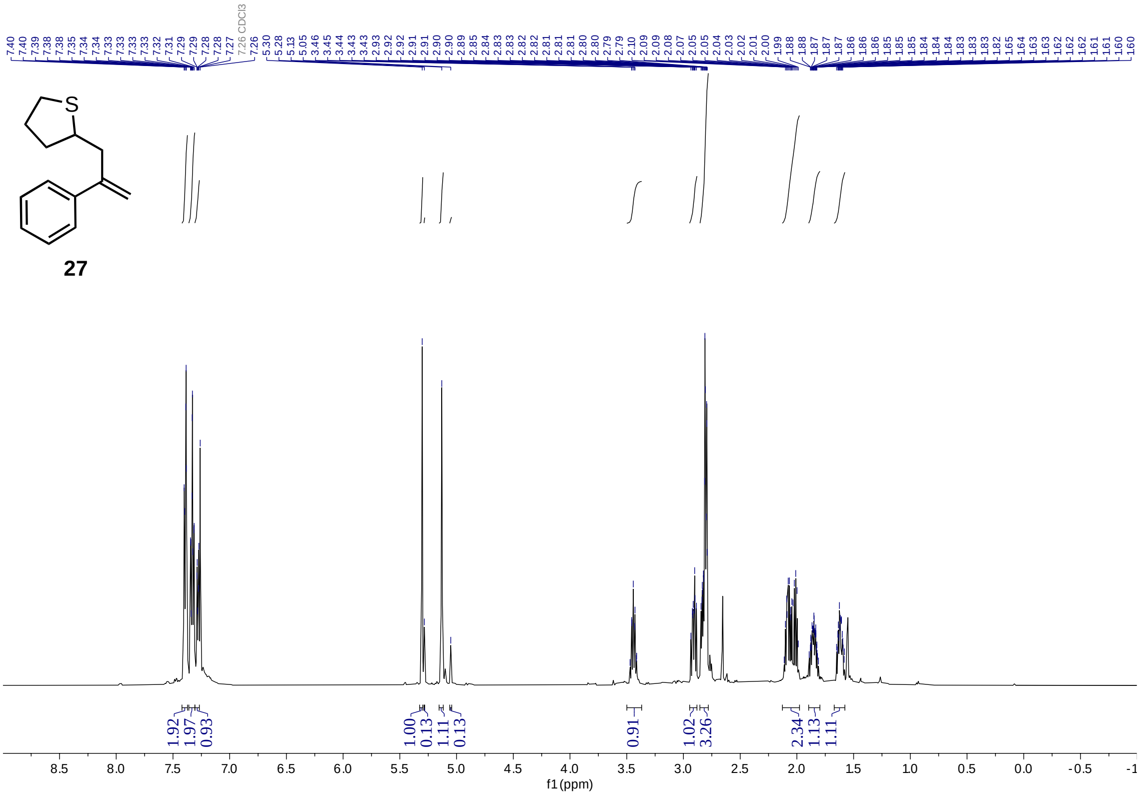

27

^{13}C NMR (126 MHz, $CDCl_3$)

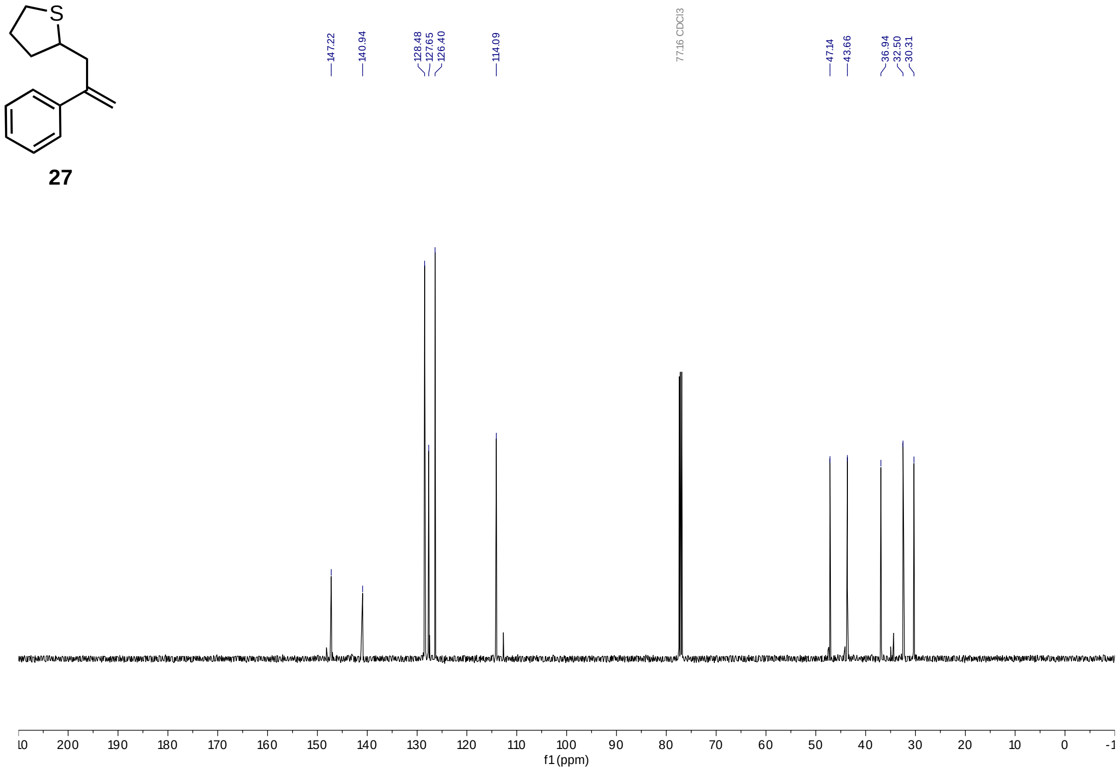

27

^{1}H NMR (500 MHz, $CDCl_3$)

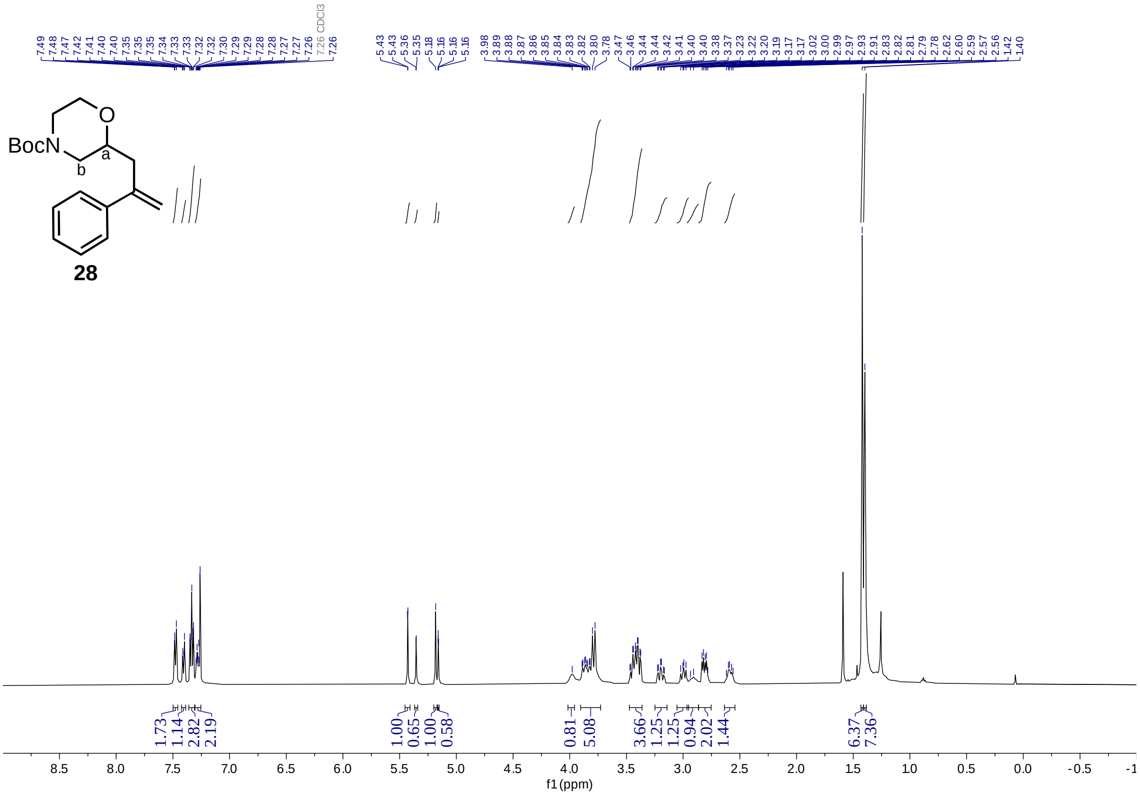

^{13}C NMR (126 MHz, $CDCl_3$)

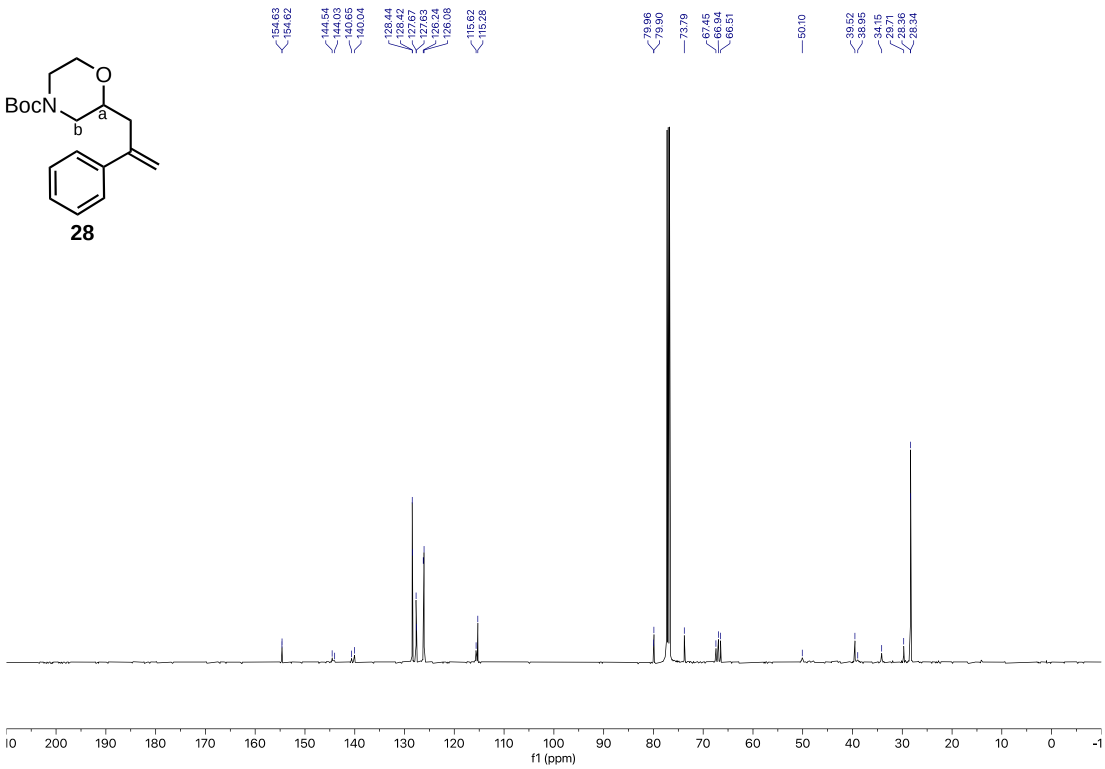

^{1}H NMR (500 MHz, $CDCl_3$)

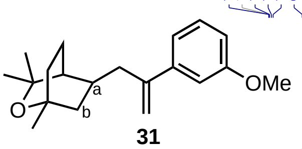

31

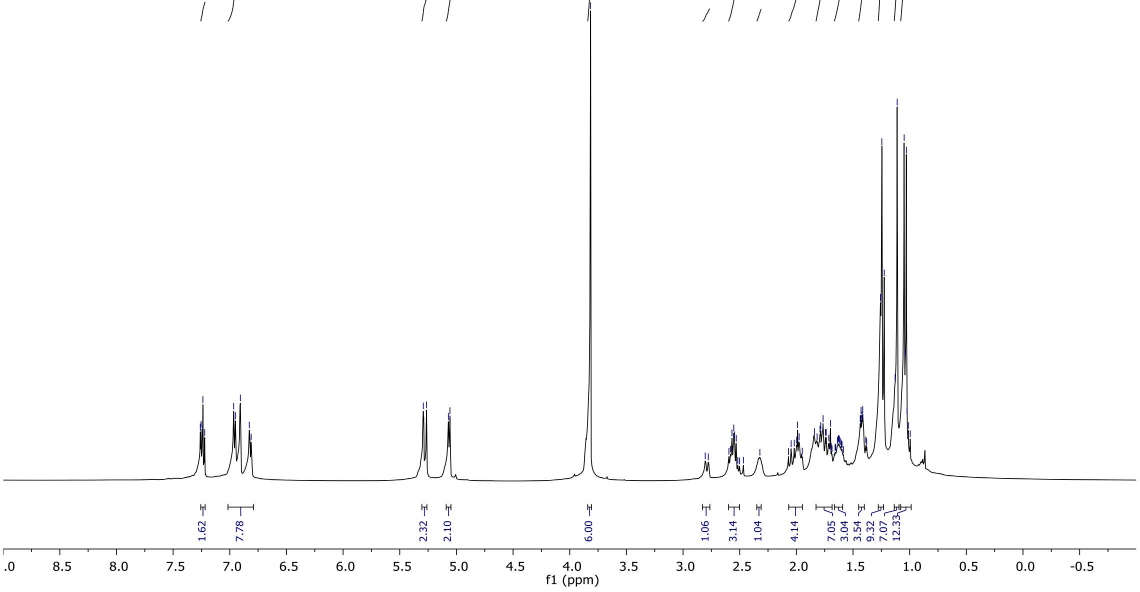

^{13}C NMR (126 MHz, $CDCl_3$)

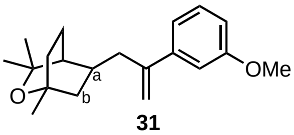

31

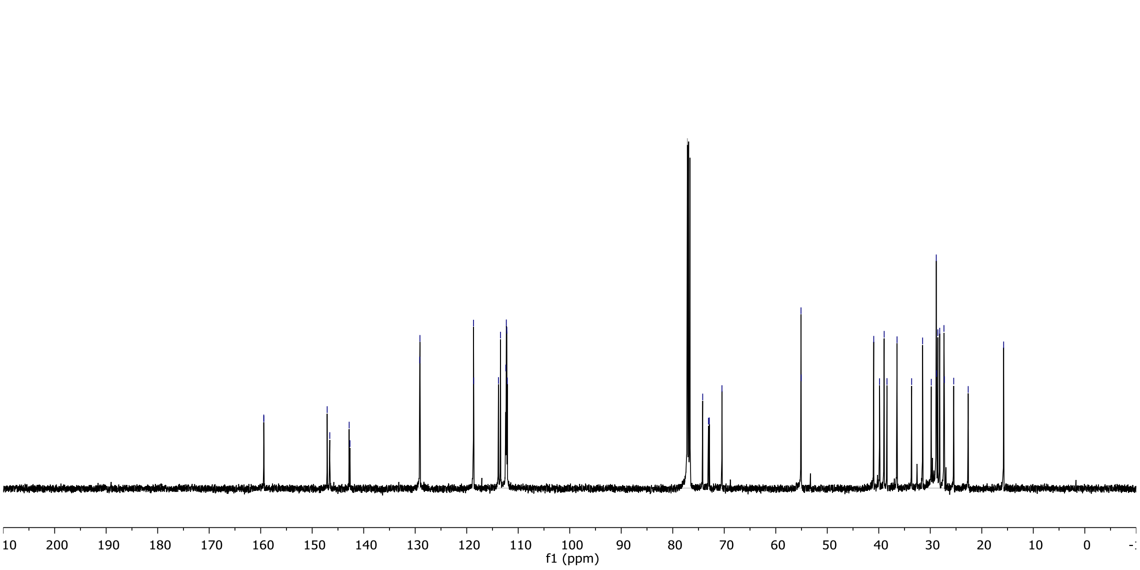

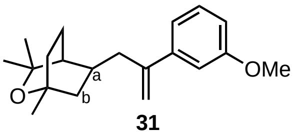

31

COSY (500 MHz, $CDCl_3$)

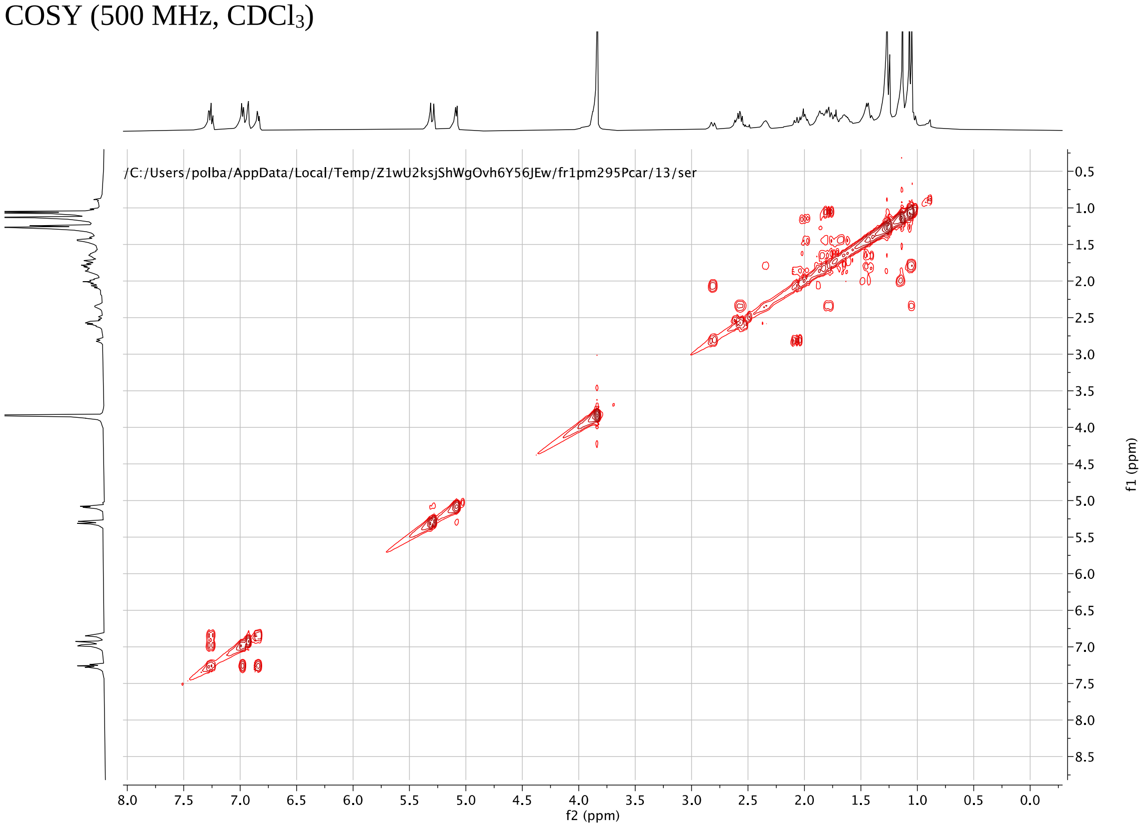

HSQC (500, 126 MHz, $CDCl_3$)

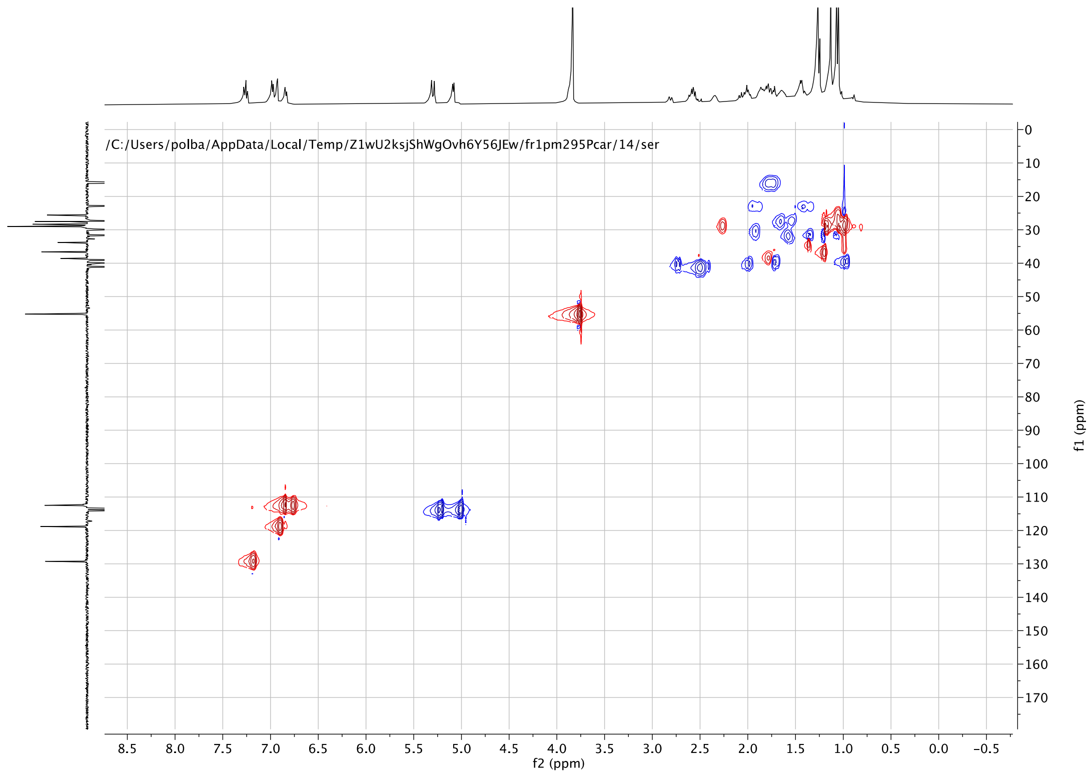

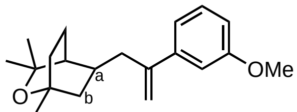

31

HMBC (500, 126 MHz, $CDCl_3$)

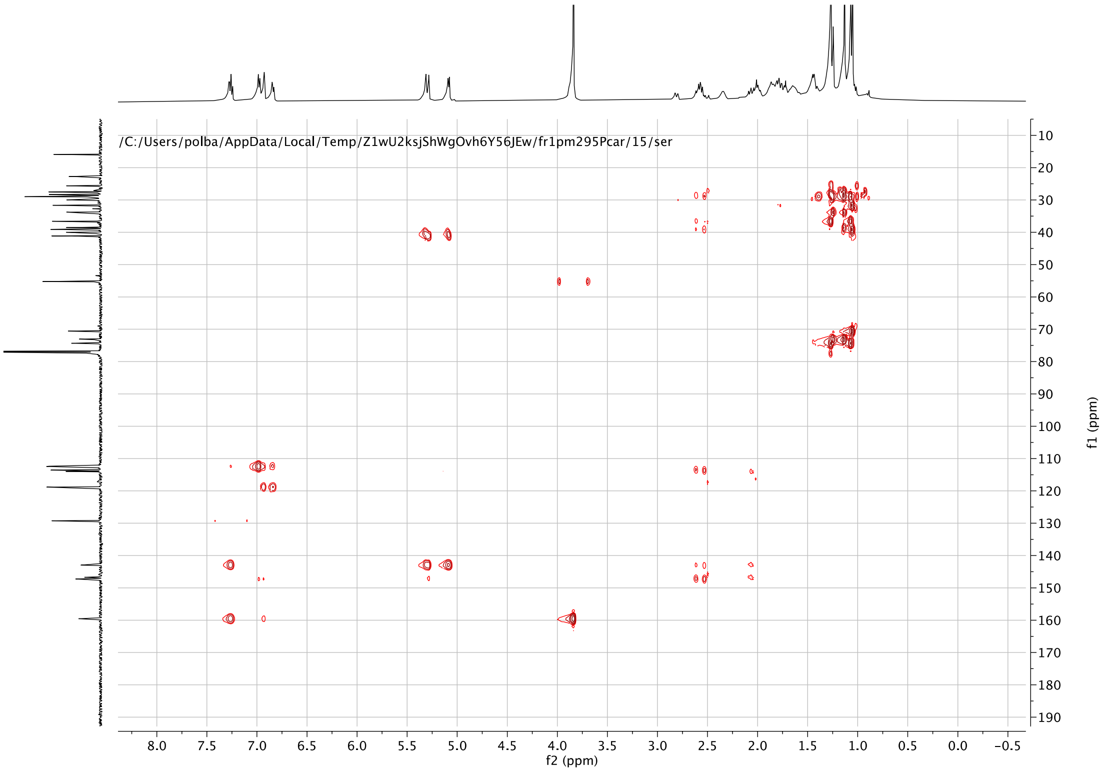

^{1}H NMR (500 MHz, $CDCl_3$)

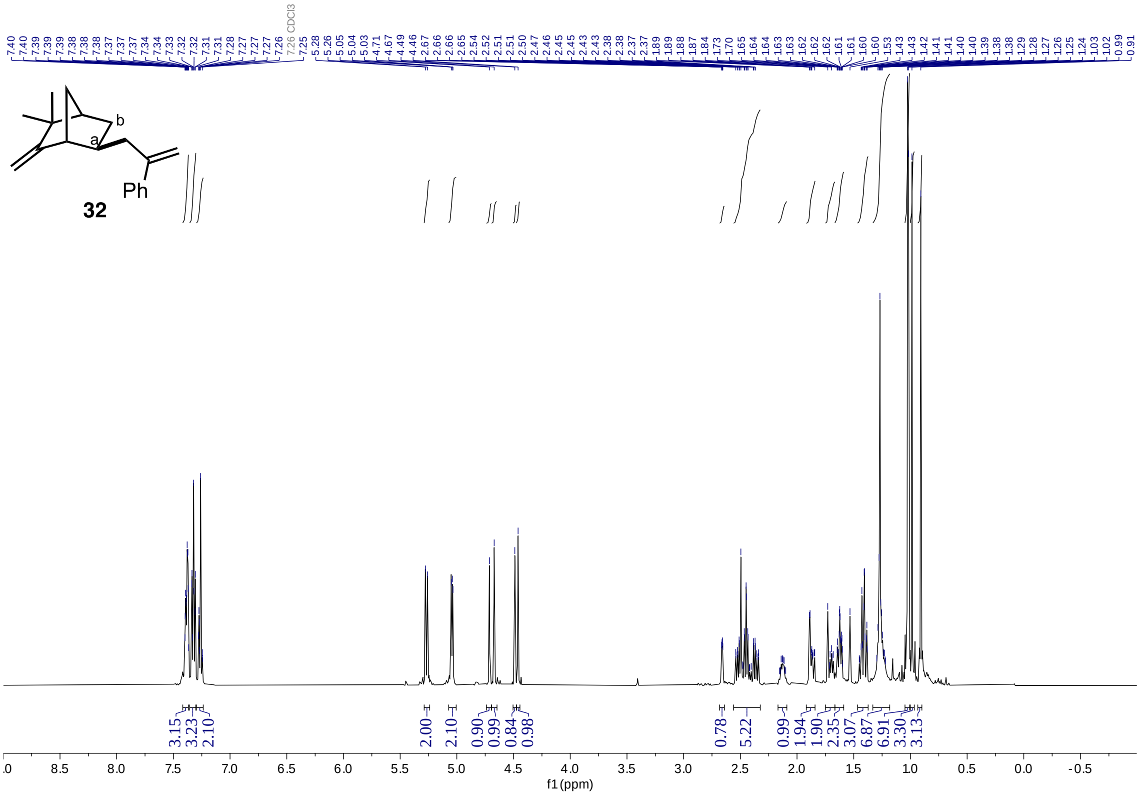

^{13}C NMR (126 MHz, $CDCl_3$)

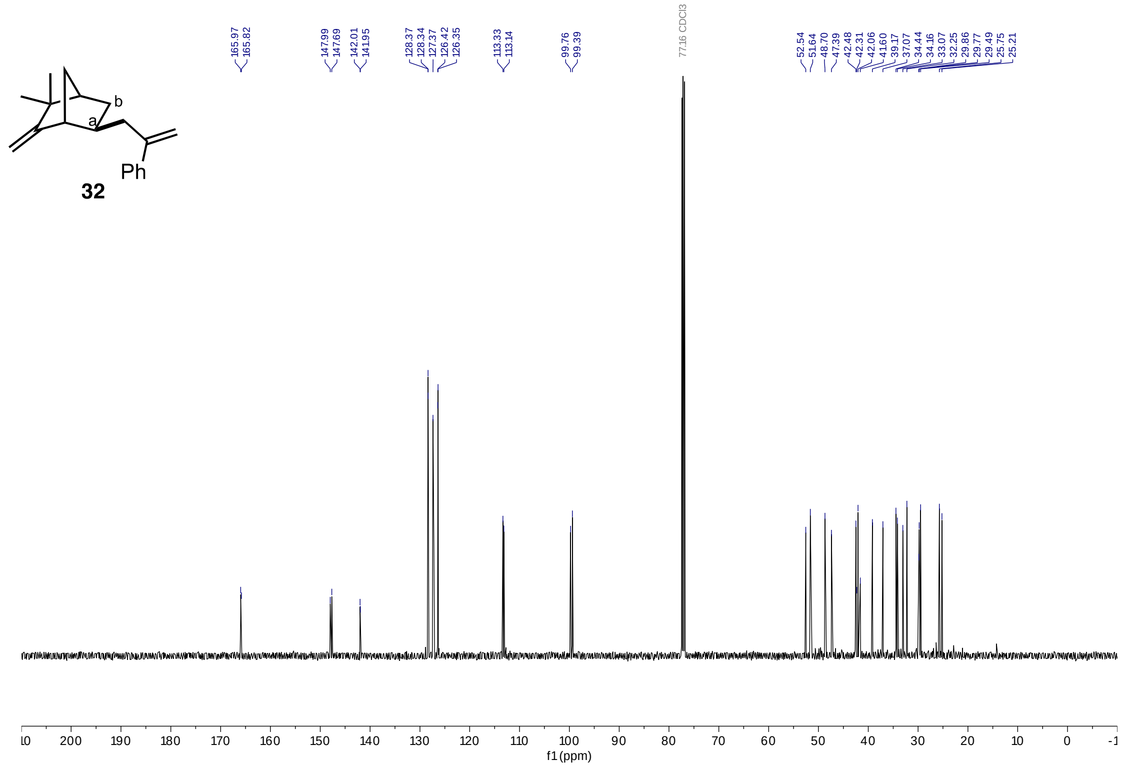

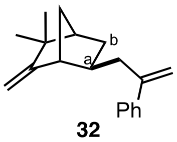

32

COSY (500 MHz, $CDCl_3$)

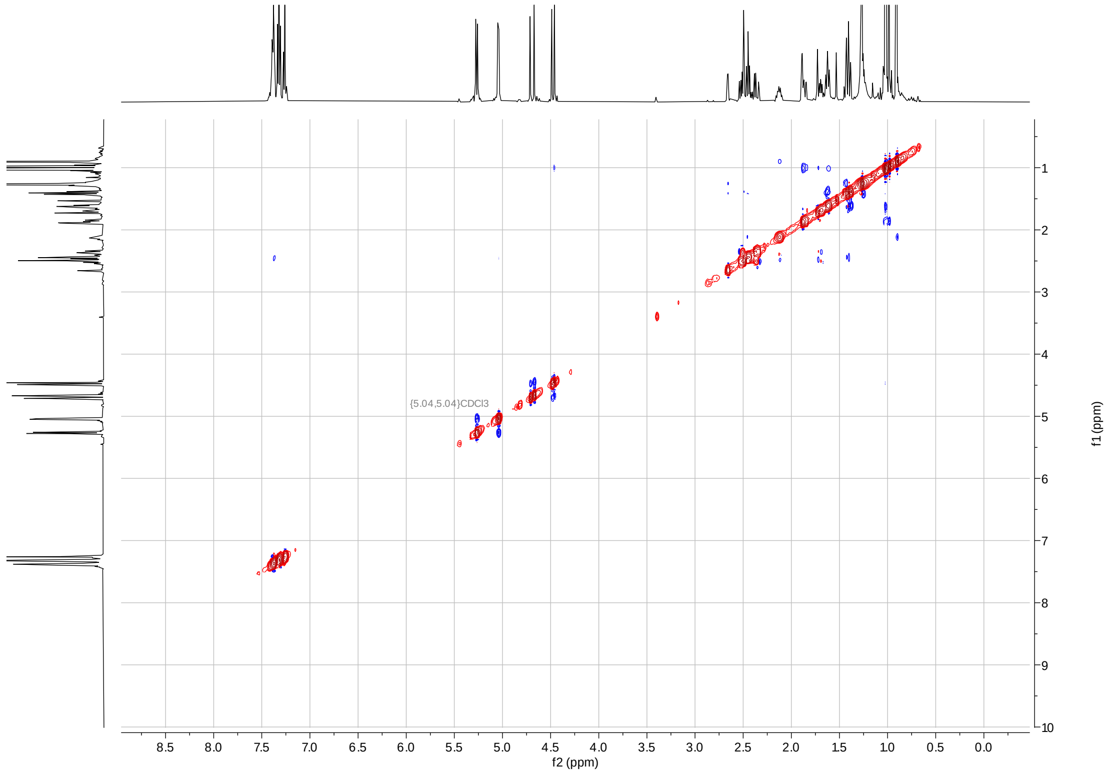

HSQC (500, 126 MHz, $CDCl_3$)

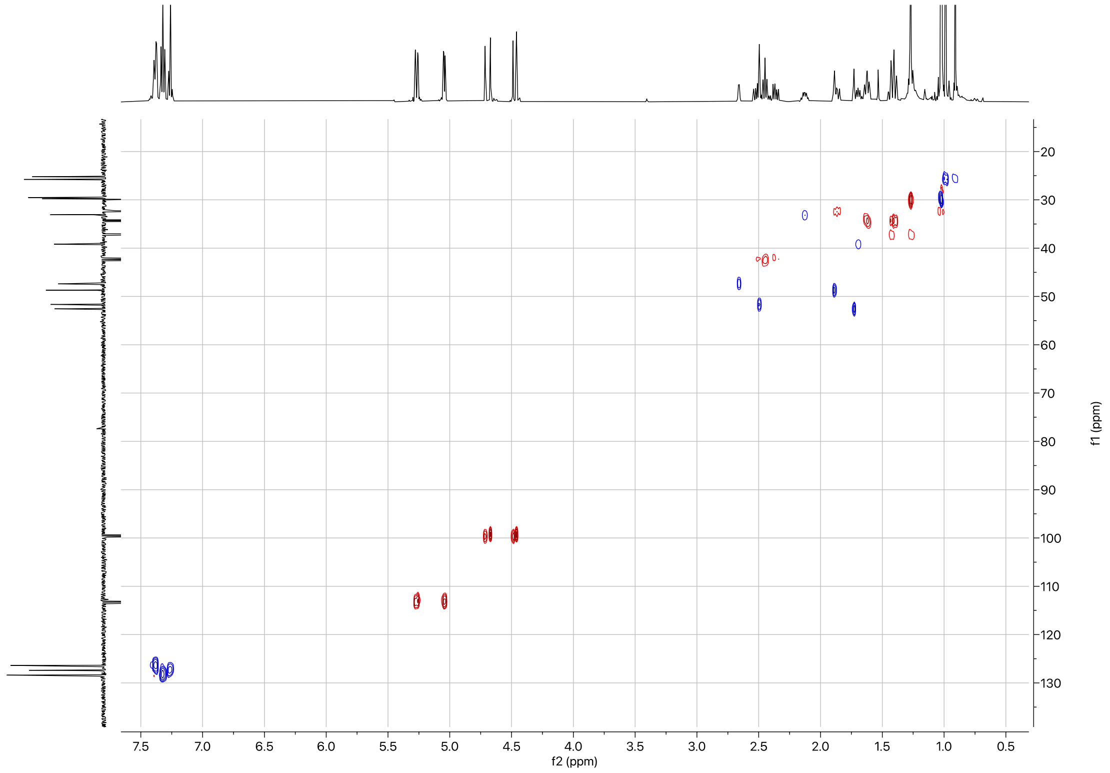

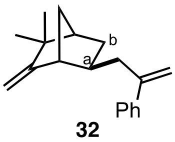

32

HMBC (500, 126 MHz, $CDCl_3$)

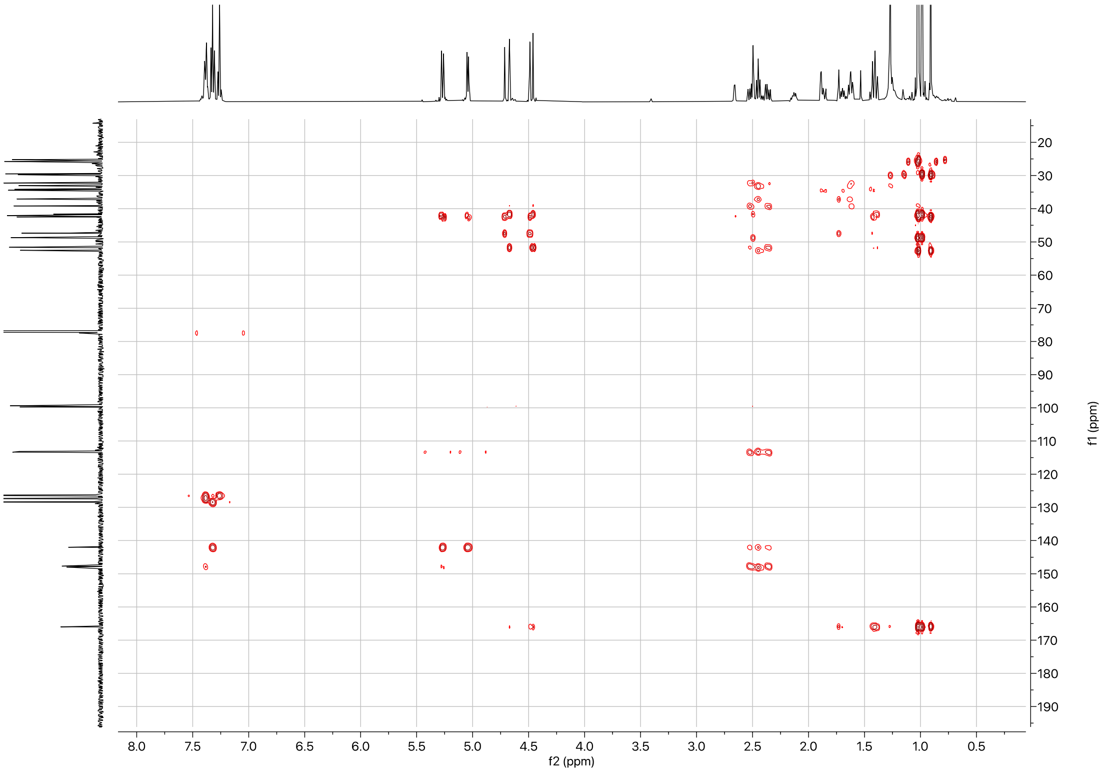

^{1}H NMR (500 MHz, $CDCl_3$)

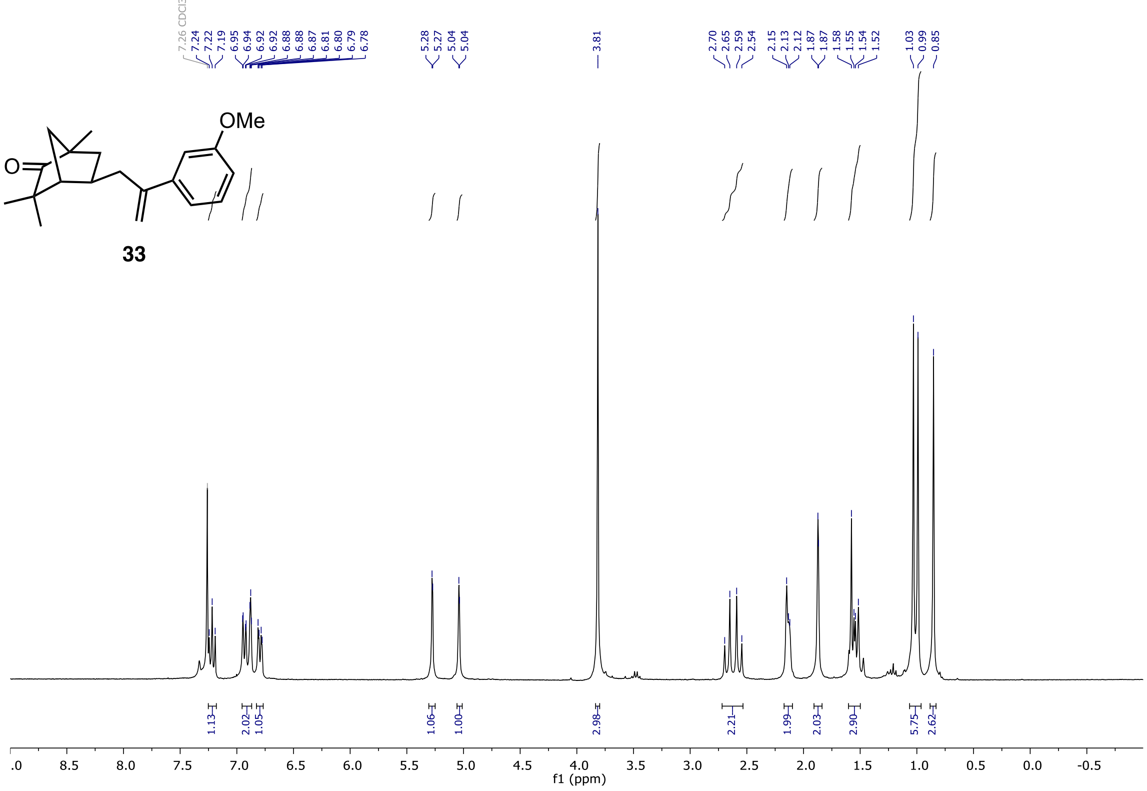

^{13}C NMR (126 MHz, $CDCl_3$)

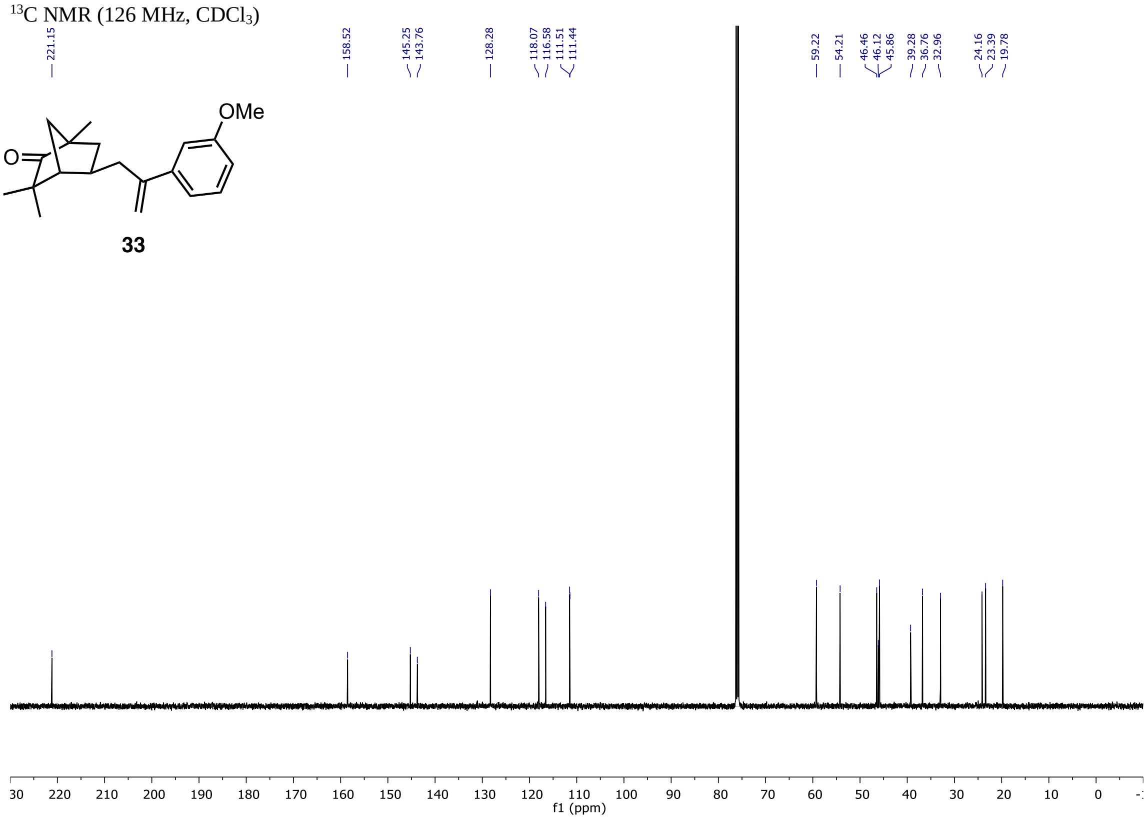

^{1}H NMR (300 MHz, $CDCl_3$)

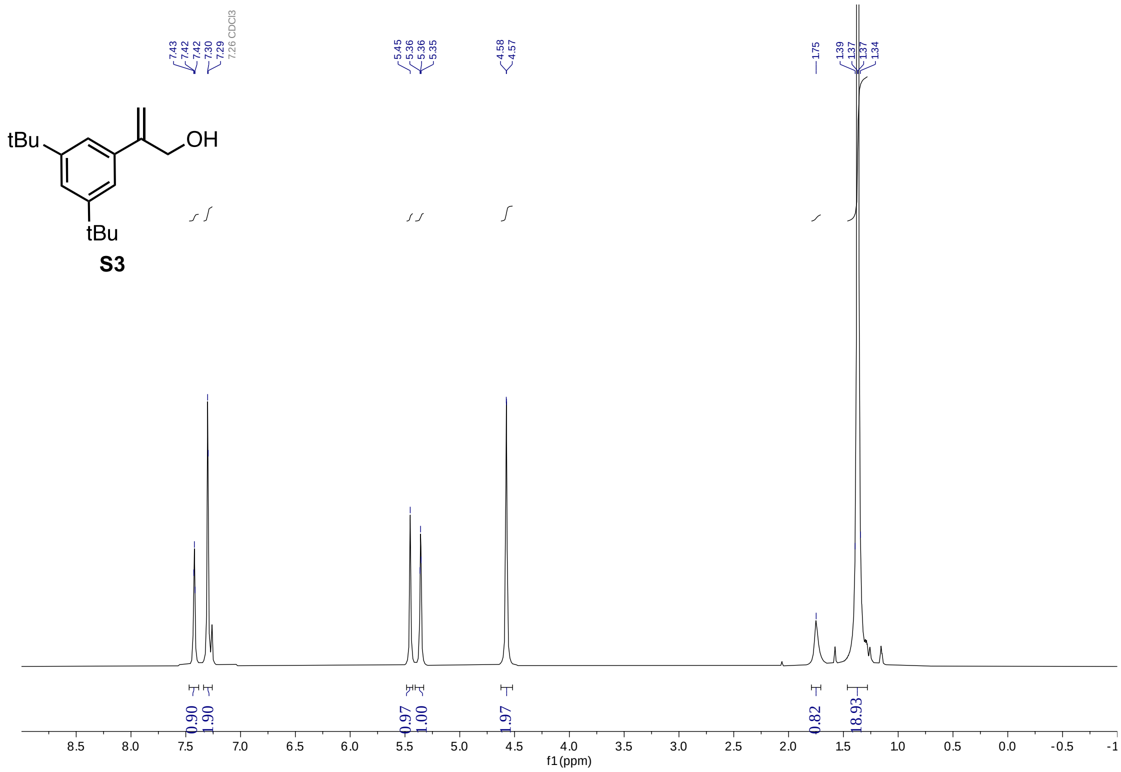

^{13}C NMR (75 MHz, $CDCl_3$)

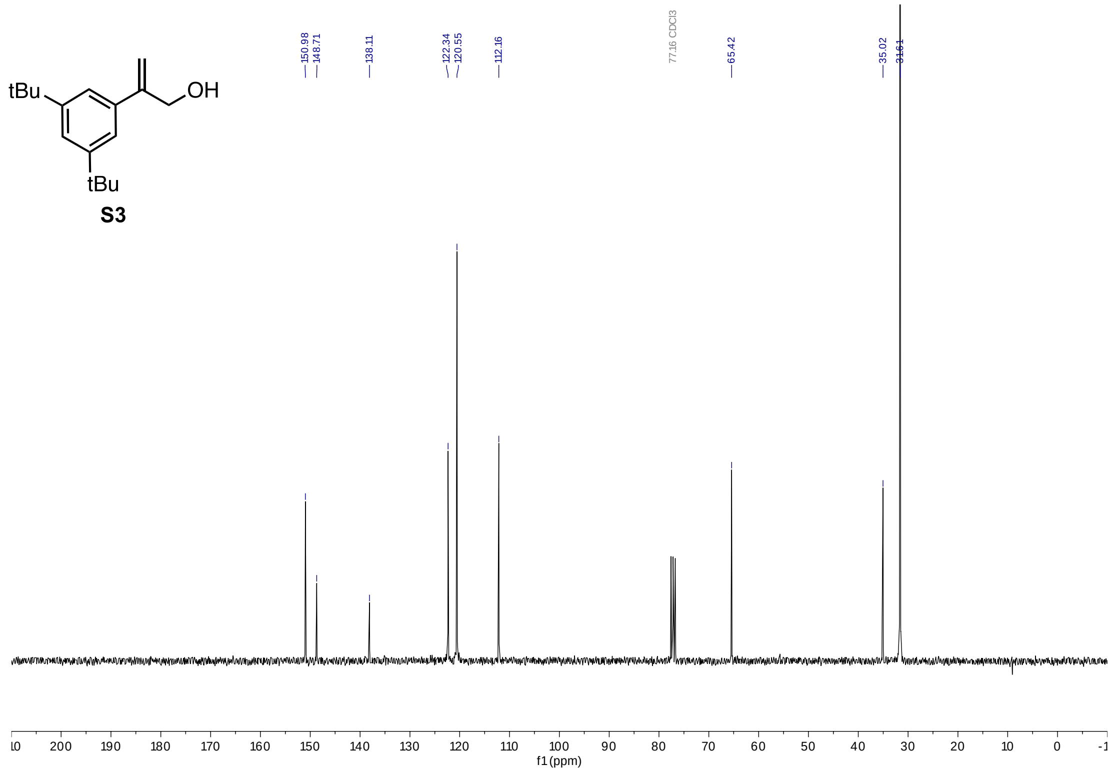

^{1}H NMR (300 MHz, $CDCl_3$)

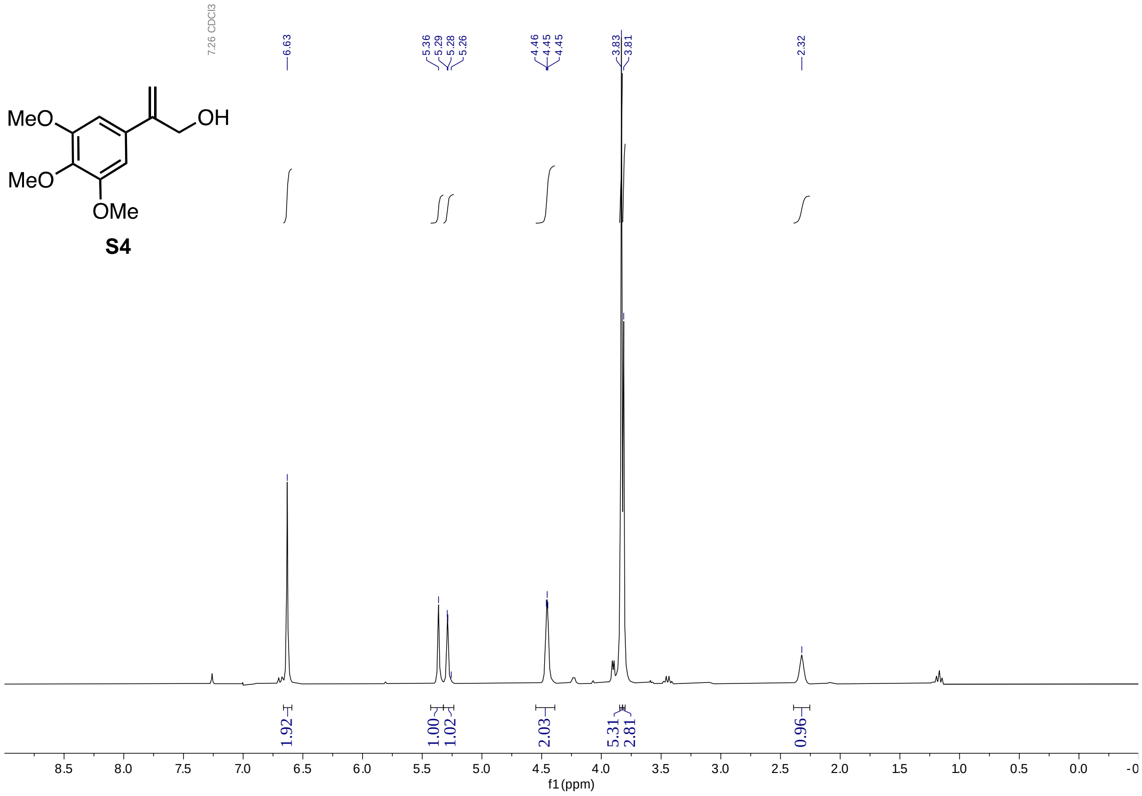

^{13}C NMR (75 MHz, $CDCl_3$)

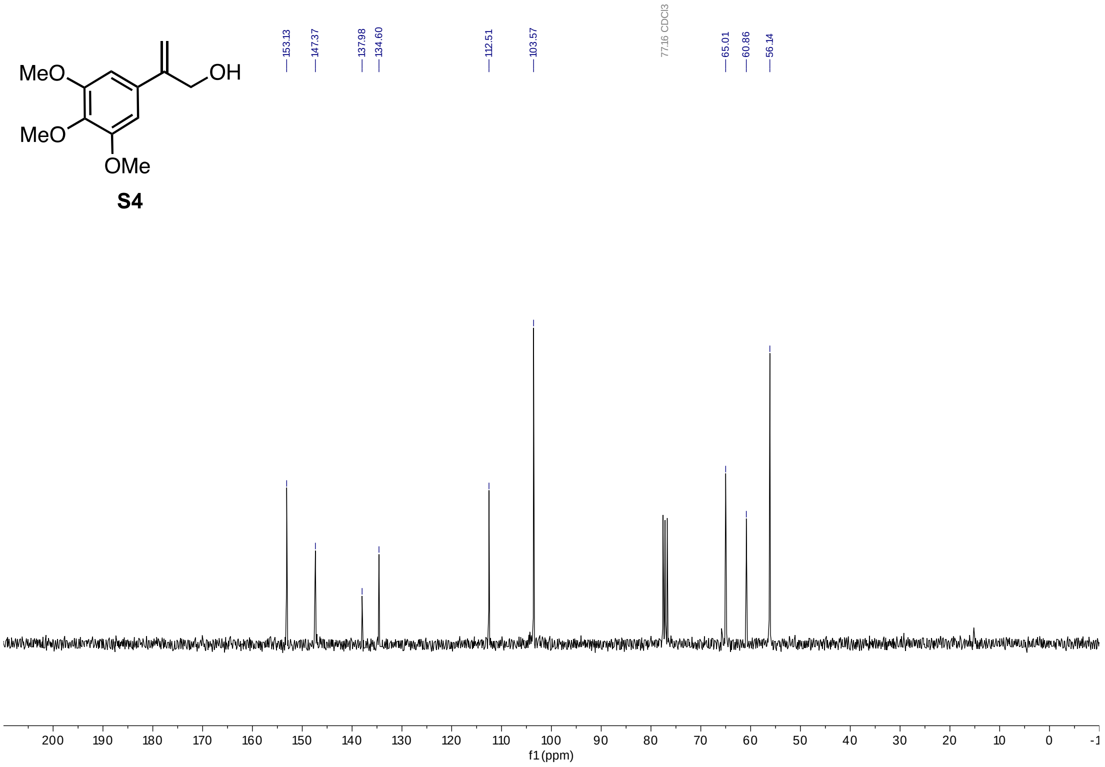

^{1}H NMR (300 MHz, $CDCl_3$)

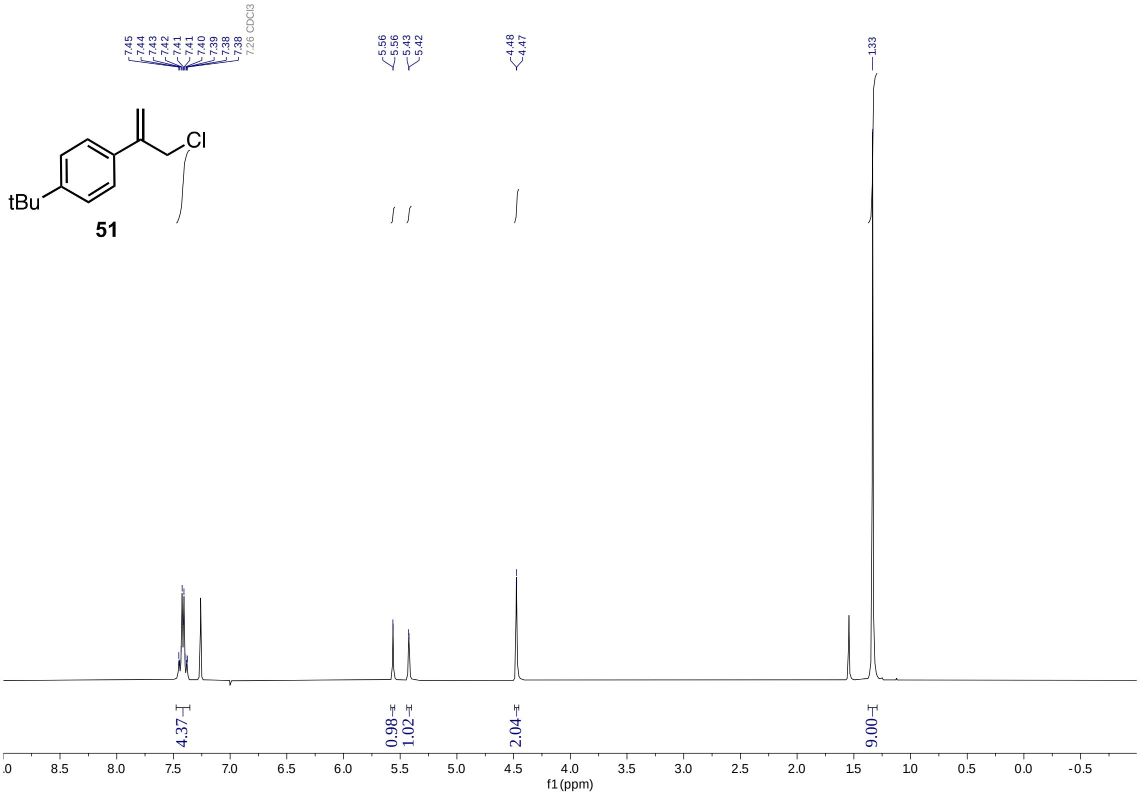

^{13}C NMR (75 MHz, $CDCl_3$)

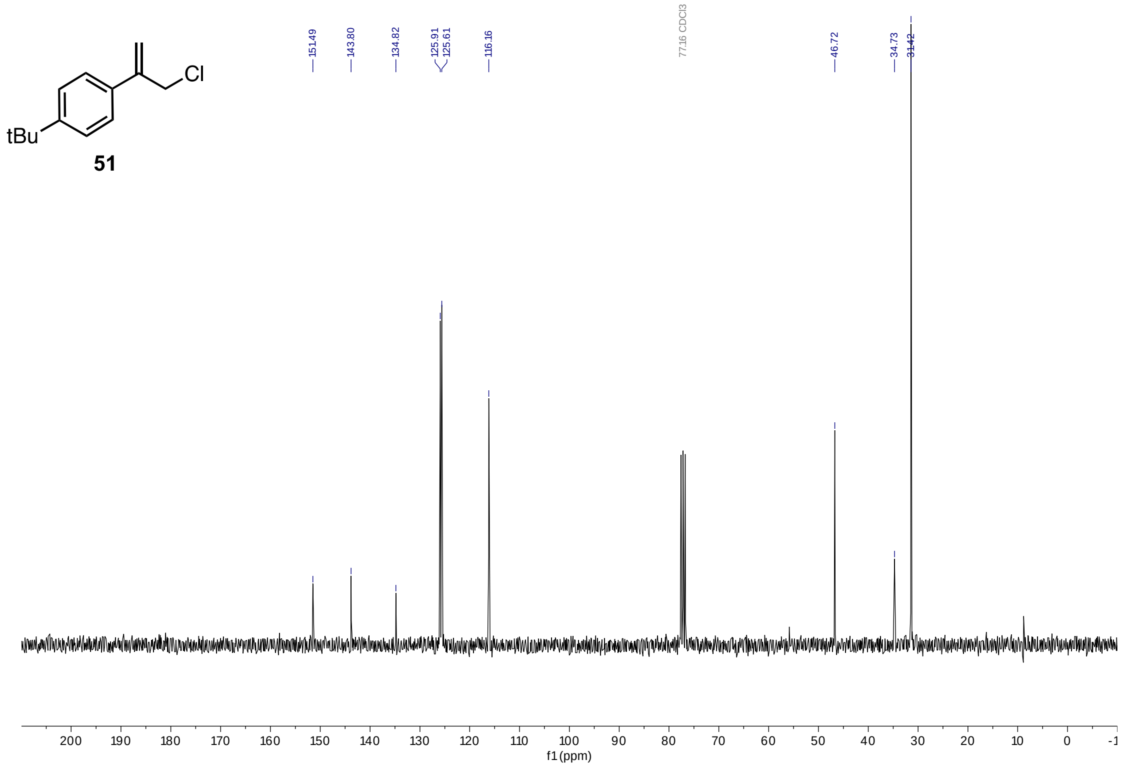

^{1}H NMR (300 MHz, $CDCl_3$)

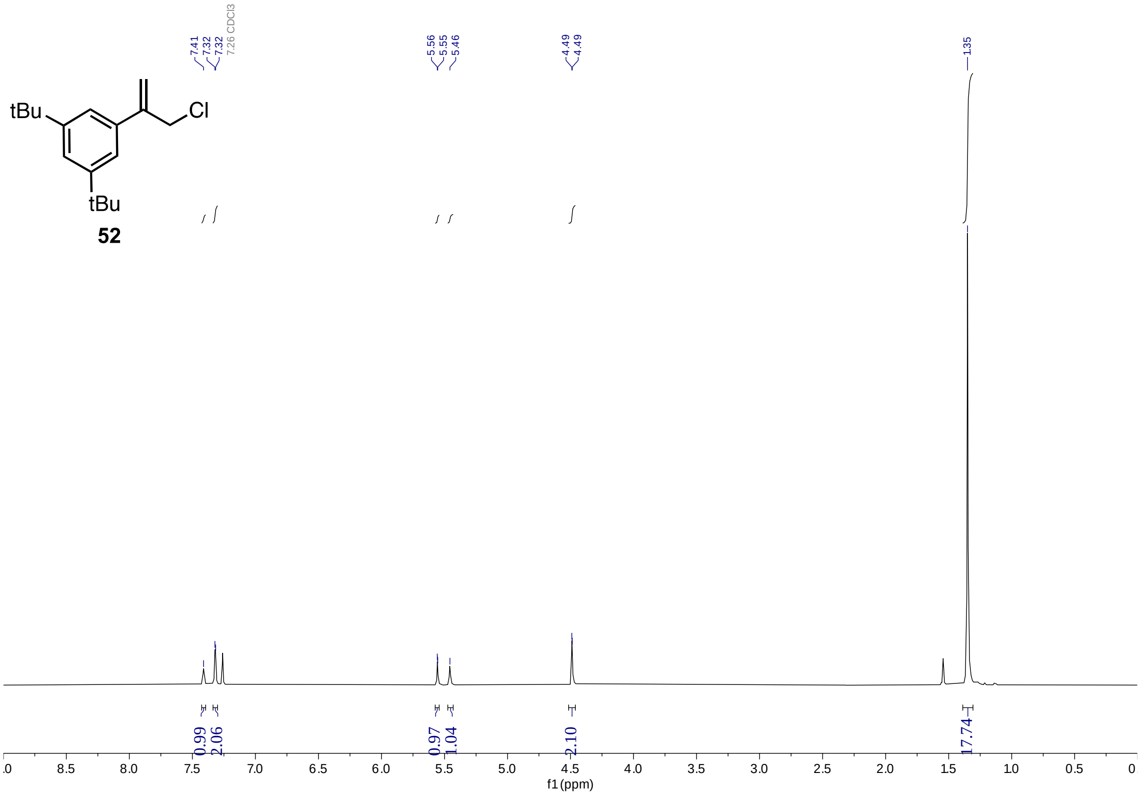

^{13}C NMR (75 MHz, $CDCl_3$)

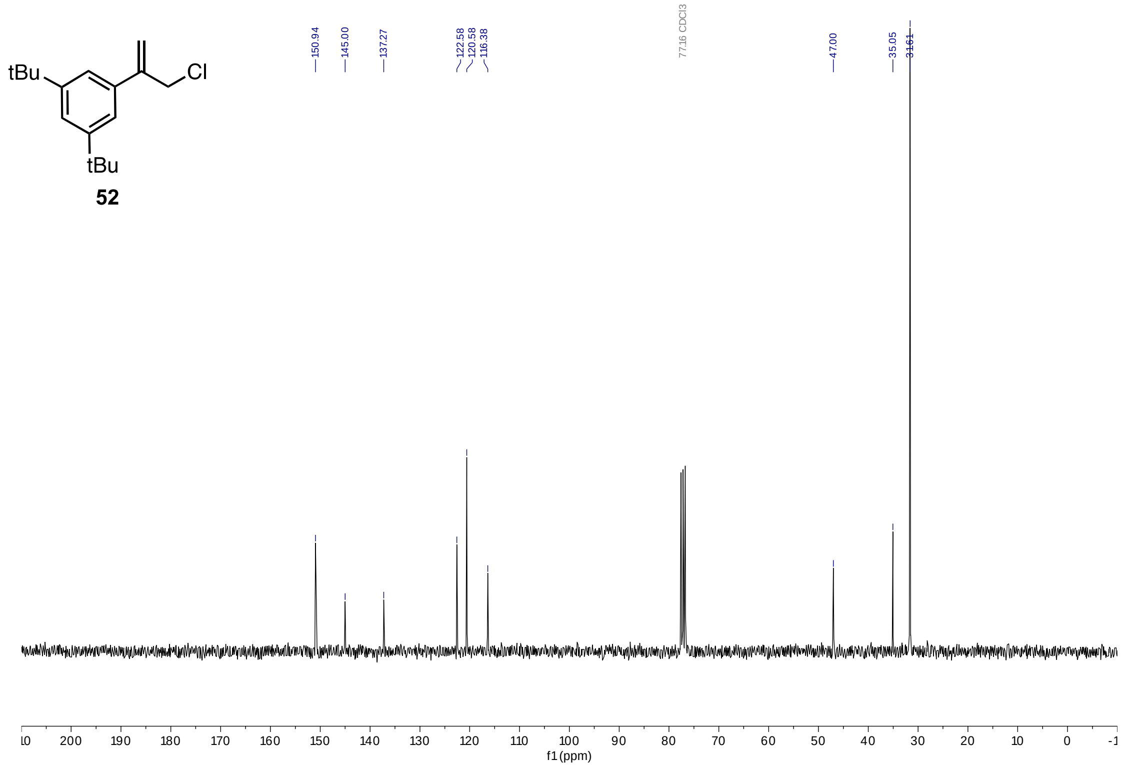

^{1}H NMR (500 MHz, $CDCl_3$)

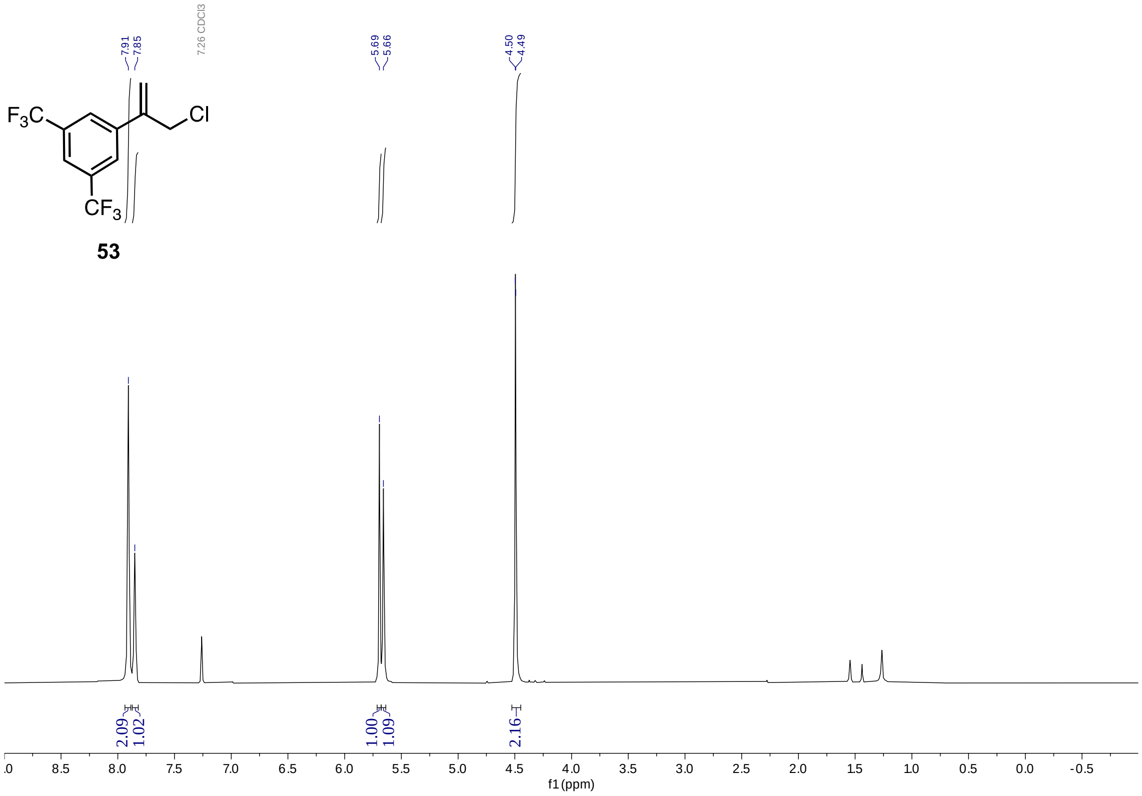

^{13}C NMR (126 MHz, $CDCl_3$)

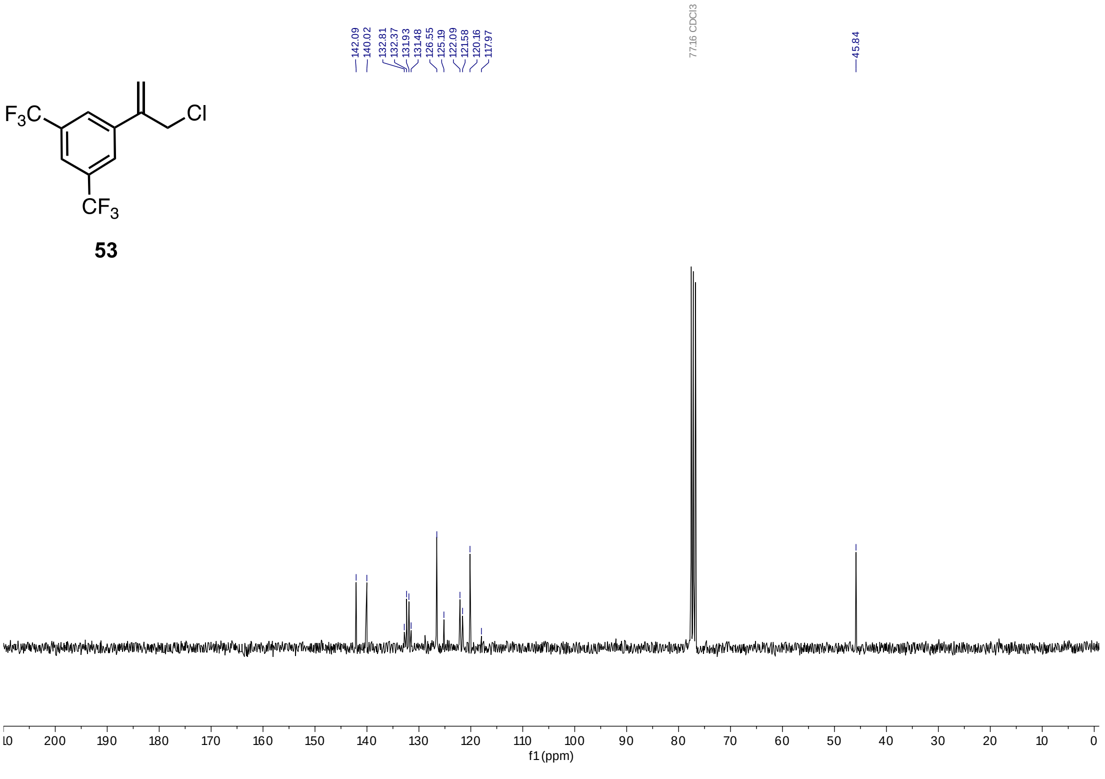

^{19}F NMR (282 MHz, $CDCl_3$)

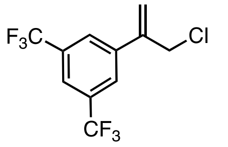

53

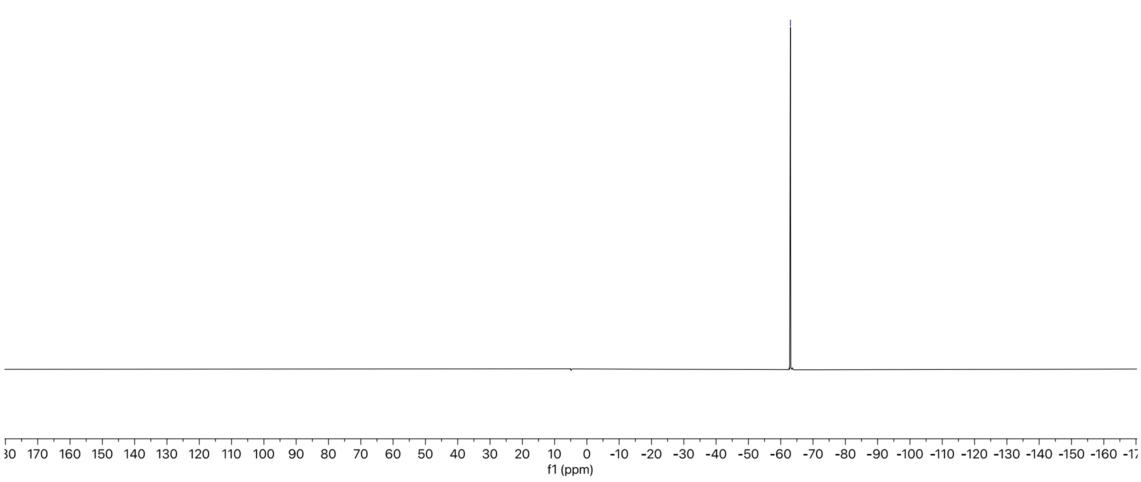

^{1}H NMR (500 MHz, $CDCl_3$)

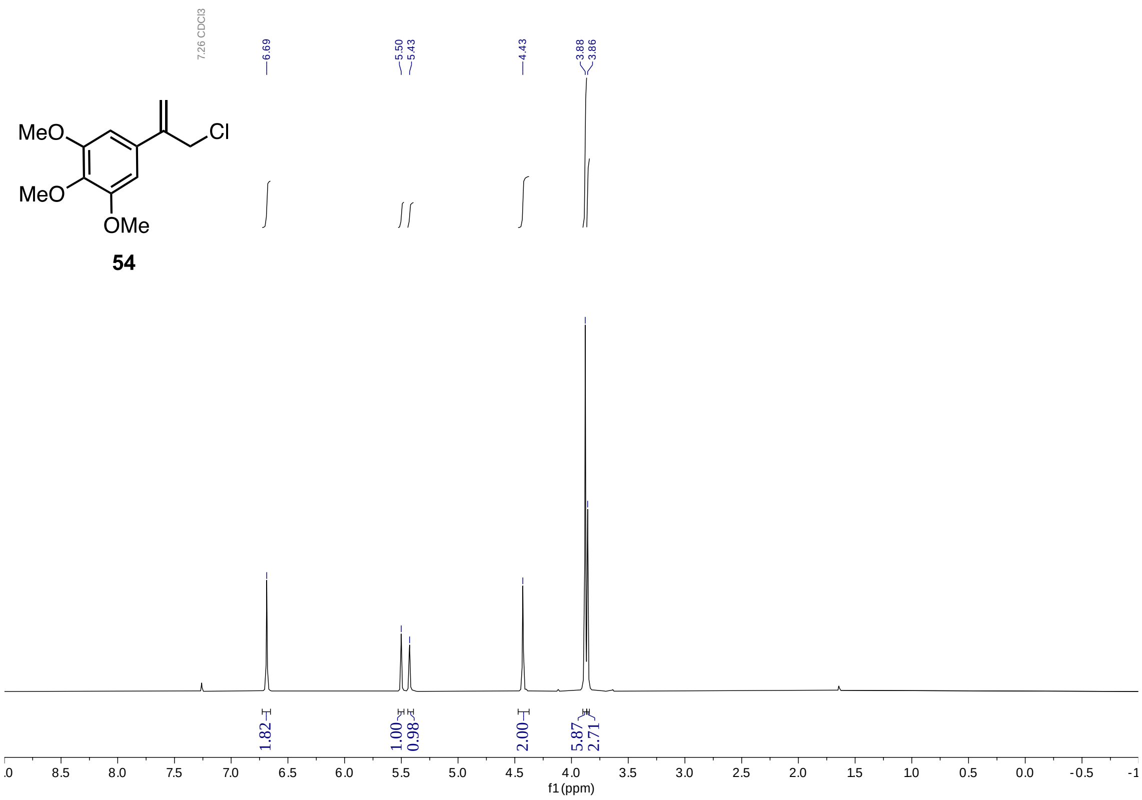

^{13}C NMR (126 MHz, $CDCl_3$)

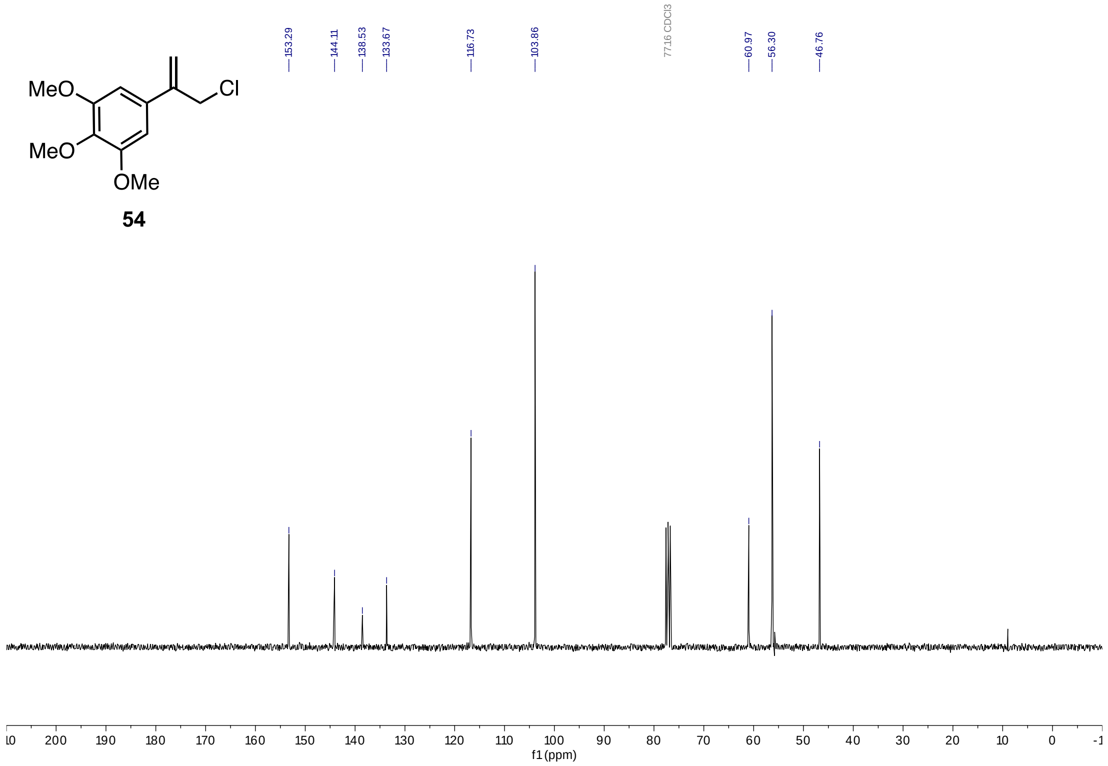

^{1}H NMR (300 MHz, $CDCl_3$)

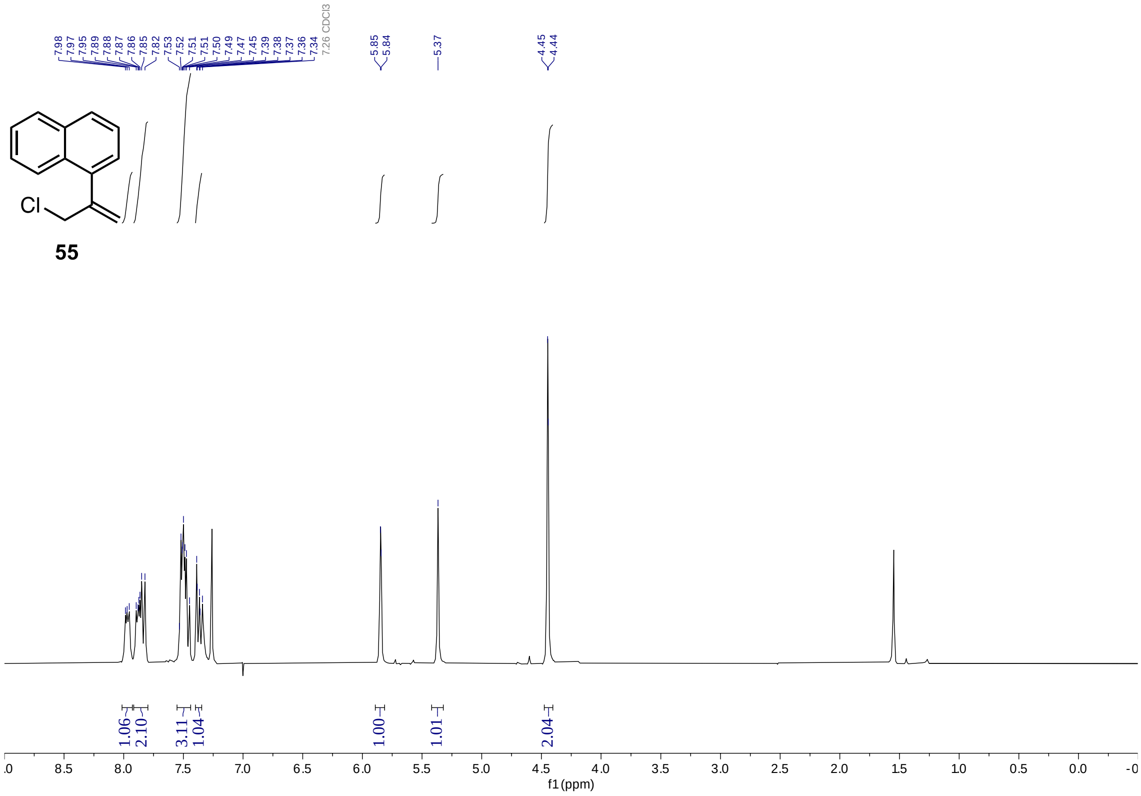

^{13}C NMR (75 MHz, $CDCl_3$)

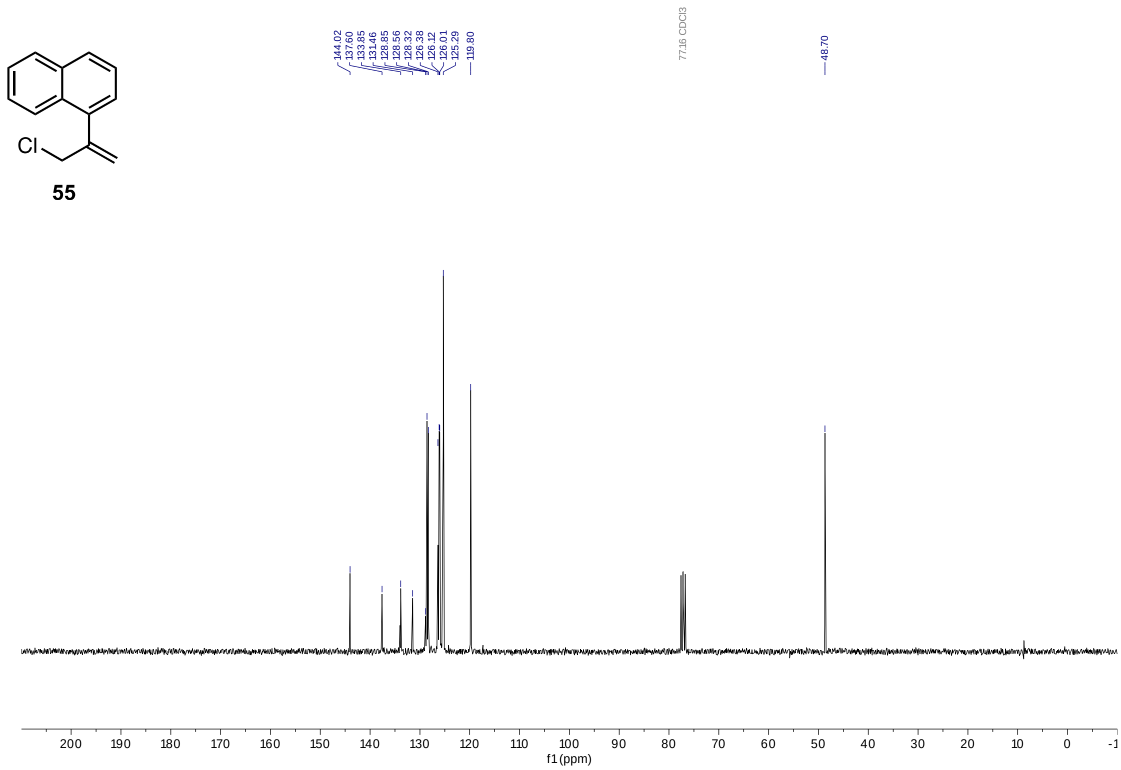

Supplement: Supplementary file 1 — ol2c02887_si_001.pdf [file ol2c02887_si_001.pdf]
